# The periphery of nuclear speckles defines a spatially and temporally regulated compartment of long-lived intron-retained RNAs that resolves during mitosis

**DOI:** 10.1038/s41556-026-02040-5

**Published:** 2026-09-09

**Authors:** Josep Biayna, Artem Baranovskii, Anusha Chaudhuri, Marta Paladin, Berkay Erdem, Luise-Elektra Keller, A. Rasim Barutcu, Stefanie Dimmeler, Annalisa Marsico, Gabrijela Dumbović

**Affiliations:** 1 Goethe University Frankfurt, Center for Molecular Medicine, Institute of Cardiovascular Regeneration, Frankfurt, Germany; 2 Cluster of Excellence Cardio-Pulmonary Institute, Goethe University Frankfurt, Frankfurt, Germany; 3 German Center of Cardiovascular Research (DZHK), partner site Rhein/Main, Frankfurt, Germany; 4 Computational Health Center, Helmholtz Center Munich, Munich, Germany; 5 ScitoVation LLC, Durham, NC USA; 6 Cluster of Excellence SubCellular Architecture of Life, Goethe University Frankfurt, Frankfurt, Germany; 7 Present Address: Berlin Institute for Medical Systems Biology, Max Delbrück Center for Molecular Medicine, Berlin, Germany

**Keywords:** Nuclear speckles, Nuclear organization

## Abstract

RNA localization adds a fundamental layer to gene expression by determining when and where translation-ready mRNAs become available, yet how this timing is coordinated with nuclear architecture and cell-cycle progression remains unclear. Here we identify a subnuclear RNA niche at the nuclear speckle periphery that couples intron retention to cell-cycle-timed RNA release. Using compartment-resolved transcriptional inhibition, sequence-based deep learning and single-molecule and super-resolution RNA imaging in human pluripotent stem cells, we define a class of nuclear RNAs with long-lived retained introns that persist for hours and are enriched in transcripts encoding regulators of genome maintenance and mitosis, including centromere and kinetochore assembly, DNA repair and telomere maintenance. Long-lived retained introns exhibit elevated GC content, predicted structural stability and enrichment for nuclear speckle-associated RNA-binding proteins. In interphase, these RNAs localize to a distinct nuclear speckle-peripheral RNA niche in a spatial arrangement conserved across cell types. During mitotic remodelling, they undergo coordinated, kinase-dependent splicing and are released into the cytoplasm of early G1 daughter cells. Together, these findings link *cis*-encoded intronic features, subnuclear organization and mitotic remodelling to temporal control of RNA fate.

## Abstract (web summary)

Biayna, Baranovskii and colleagues report that intron-retained RNAs derived from cell cycle- and genome maintenance-related genes are pooled at the periphery of nuclear speckles and undergo synchronous splicing at mitosis, thereby contributing to their temporal regulation.

## Main

Spatial and temporal RNA control governs when and where mRNAs become accessible for translation, shaping development, cell polarization and stress responses, yet many transcriptomic approaches lack the resolution needed to determine where RNAs reside, how long they persist there or how localization relates to function^1–9^. RNA mislocalization contributes to disease development, making these principles clinically relevant^10–14^.

Persistence of an intron in otherwise mature RNA, known as intron retention (IR), is linked to nuclear RNA retention, transcriptome diversification and rapid gene activation in response to external cues, with implications in differentiation, stress adaptation and disease development^8,15–25^. Retained introns (RIs) are functionally diverse and can occur in cytoplasmic transcripts that are translated or degraded, or whose RIs direct RNA localization; in unstable transcripts cleared through RNA-surveillance pathways; and in nuclear transcripts whose RIs can be removed in response to specific cues, a class termed detained introns^8,15,17,18,21,26–35^. Detained introns established nuclear IR as a regulated RNA state rather than inefficient splicing. However, it remains unclear whether nuclear IR comprises kinetically distinct populations with different residence times and regulatory behaviours, how these populations are spatially organized, whether specific microenvironments coordinate their timed release, and how physiological transitions involving large-scale nuclear reorganization, such as mitosis, couple to their regulation.

Among nuclear membraneless organelles^36^, nuclear speckles are enriched in splicing and mRNA processing factors and have been proposed to act as hubs for RNA processing, nuclear mRNA retention and export licensing^37–42^. They contain concentrated serine and arginine-rich (SR) proteins and other splicing regulators whose phosphorylation controls their partitioning to nuclear speckles and splicing activity^43^. CDC-like kinases (CLKs) and dual-specificity tyrosine-phosphorylation-regulated kinase 3 (DYRK3) regulate phosphorylation-dependent remodelling of nuclear speckles and their associated splicing factors^44–47^. Thus, nuclear speckles represent dynamic, kinase-regulated environments in which RNA processing, retention and release are spatially coordinated.

Splicing can occur co- and post-transcriptionally^48–57^. Nascent transcripts from strongly induced genes localize near nuclear speckles^58^, and highly active transcription sites can generate enlarged nuclear speckle-like domains, supporting coupling between transcriptional activity and nuclear speckles^59^. Recent proximity-labelling studies showed that intron-retained (IR-) RNAs frequently associate with nuclear lamina or nuclear speckle markers^60^; however, these approaches lack the resolution to define the precise spatial relationship of IR-RNAs with nuclear speckles or to distinguish condensate association from signals arising from transcription sites, often near nuclear speckles^61–64^.

Here, we combine compartment-resolved transcriptional inhibition, sequence-based deep learning and RNA imaging in human induced pluripotent stem (hiPS) cells and primary human umbilical vein endothelial cells (HUVECs) to define the kinetic and spatial organization of IR-RNAs. We identify a class of nuclear IR-RNAs with long-lived IR (LL-IR-RNAs) in hiPS cells that exhibits elevated IR levels and enrichment in genome maintenance and mitosis-linked pathways. High-resolution single-molecule RNA (smRNA) imaging revealed that poly(A)^+^ IR-RNAs occupy a distinct spatial niche compared with bulk poly(A)^+^ RNAs: whereas global poly(A)^+^ RNAs accumulate within nuclear speckles^38,41,42^, IR-RNAs are enriched at the nuclear speckle periphery, with LL-IR-RNAs more proximal to and distributed around multiple nuclear speckles, defining a juxta-speckle RNA niche conserved across cell types.

Sequence-based deep learning distinguished retained from efficiently spliced introns and long-lived from short-lived RIs, revealing that intron longevity is associated with elevated GC content, structured RNA elements, nuclear speckle-associated RNA-binding proteins (RBPs) and proximity, indicating that IR-RNAs reside in a splicing-permissive environment where *cis*-inhibitory features may delay intron removal. LL-IR-RNAs undergo coordinated, kinase-dependent intron removal in mitosis and populate early G1 daughter cells as cytoplasmic spliced transcripts, consistent with prolonged intron detention followed by a major mitotic splicing wave. Together, our data link *cis*-encoded intronic features, nuclear organization and mitotic remodelling to the temporal control of RNA fate, suggesting that nuclear storage and mitotic release of RNAs encoding genome maintenance and cell cycle regulators can promote their inheritance into early G1 daughter cells.

## Results

### IR associates with distinct nuclear localization patterns in hiPS cells

**Fig. 1:**
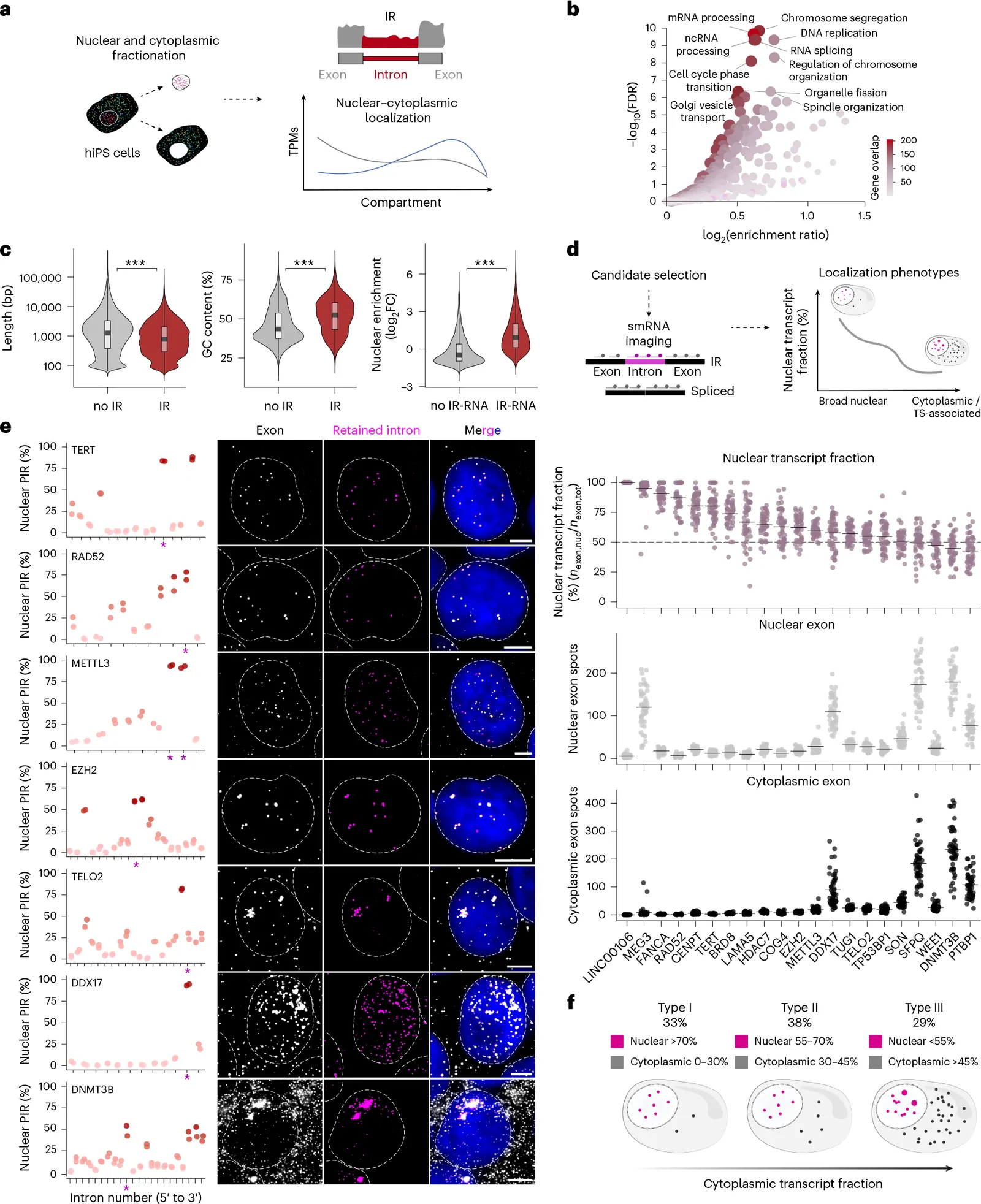
IR is associated with distinct nuclear RNA localization patterns in hiPS cells. **a**, Schematic of the experimental design. **b**, GO analysis of genes with IR (nuclear PIR ≥30%). **c**, Violin plots showing length (left) and GC content (middle) of RIs (IR; *n* = 12,019 introns) and spliced introns (no IR; *n* = 73,704 introns). Intron length is displayed on a log_10_ scale. Right: nuclear enrichment (log_2_(TPM_nuc_/TPM_cyto_)) of host RNAs containing at least one RI (IR-RNA; *n* = 5,686 transcripts) or only spliced introns (no IR-RNA; *n* = 4,468 transcripts). Midline, median; box limits, first and third quartiles; whiskers, most extreme values within 1.5× interquartile range (IQR) of the hinges. ***P ≤ 0.001, two-sided Welch’s t-test. All comparisons, *P* < 2.2 × 10^−16^. **d**, Schematic of the smRNA FISH experimental and analysis workflow. Co-localized exon and intron signals were classified as IR-transcripts, whereas exon-only signals were classified as spliced transcripts. TS, transcription site. **e**, Left: dot plot showing nuclear PIR of individual introns for the indicated genes, calculated using vast-tools from nuclear poly(A)^+^ RNA-seq (*n* = 2). Introns selected for smRNA FISH are marked with an asterisk. Middle: representative smRNA FISH maximum intensity projections. Exon, white; intron, magenta; nucleus, blue; scale bar, 2 μm. Right: smRNA FISH quantification of nuclear transcript fraction (%) (*n*_exon,nuc_ /*n*_exon,total_), nuclear exon spots and cytoplasmic exon spots (*n* = 50 cells per gene, except *TERT* (*n* = 55), two independent stainings). Dots, individual cells; horizontal lines, means. Additional representative images are shown in Extended Data Fig. 1b. **f**, Schematic of observed spatial distribution patterns of IR-RNAs. Arrow indicates increasing cytoplasmic transcript fraction. Source data

**Extended Data Fig. 1:**
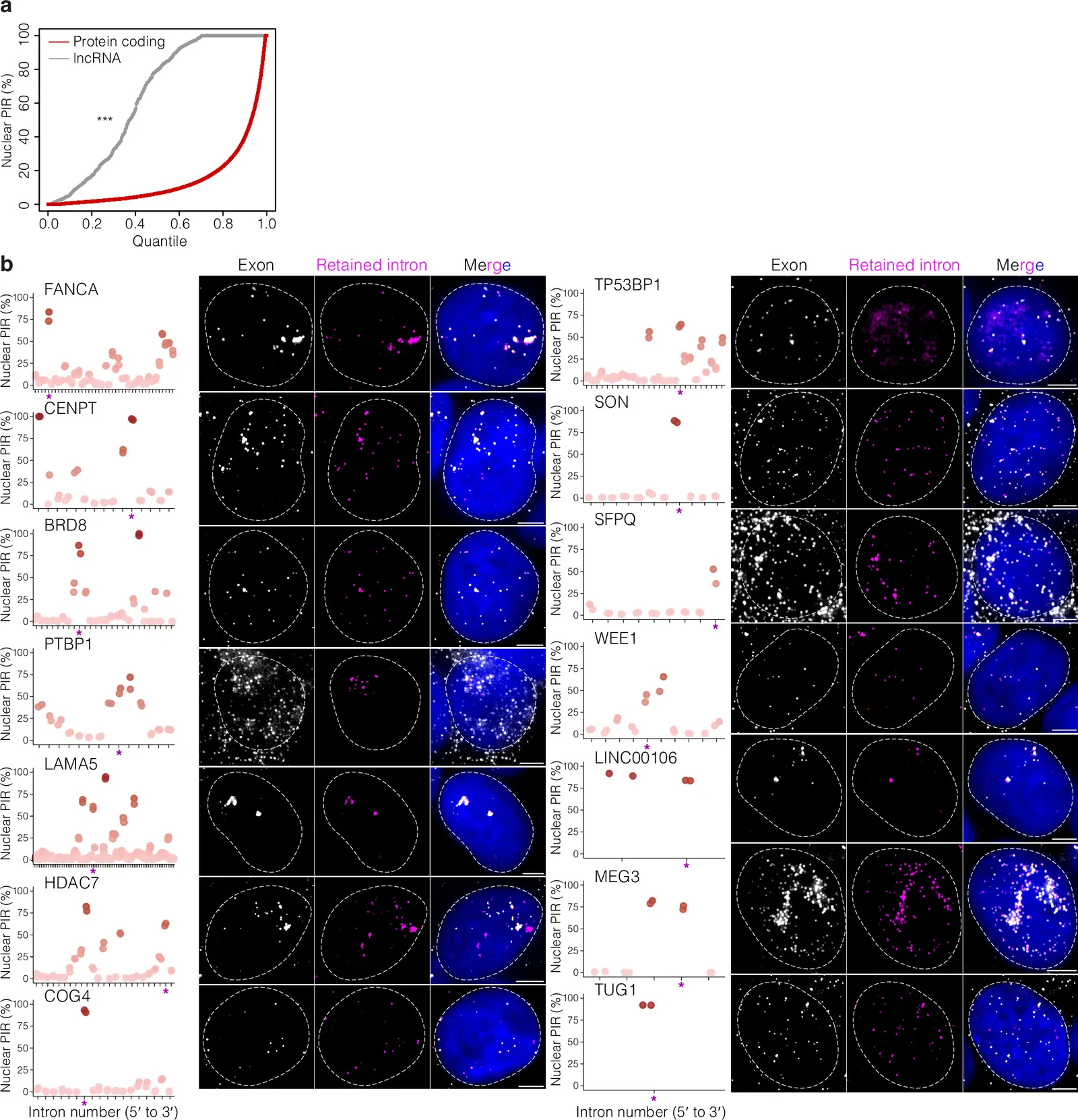
IR associates with distinct nuclear RNA localization patterns in hiPSCs. **a**, Cumulative distribution of nuclear PIR for coding (red) and lncRNA (gray) genes. ***P ≤ 0.001, evaluated by Kolmogorov-Smirnov test. *P* < 2.2 × 10^−16^. **b**, Dot plot showing nuclear PIR of individual introns for indicated genes (*n* = 2) obtained by vast-tools from poly(A)^+^ nuclear RNA-seq. Introns selected for smRNA FISH are indicated with purple asterisks. On the right, representative smRNA FISH maximum intensity projections. Exon, white; intron, magenta; nucleus, blue, outlined with a dashed line; scale bar, 2 µm. Source numerical data are available in source data. Source Data

To examine the association of IR with RNA compartmentalization in hiPS cells, we biochemically fractionated cells into nuclear and cytoplasmic fractions, performed poly(A)^+^ RNA sequencing (RNA-seq) and quantified IR (Methods; Fig. 1a). As expected, long noncoding RNAs (lncRNAs) exhibited higher nuclear percentage of IR (PIR) than mRNAs^16,17,23,65^ (Extended Data Fig. 1a). We applied a stringent PIR threshold (≥30%) to define IR to minimize nascent RNA background. Gene Ontology (GO) analysis showed IR-RNA enrichment in mRNA processing, chromatin regulation, splicing, cell cycle and DNA replication pathways (Fig. 1b), similar to studies in mouse and quiescent stem cells^17,21^. RIs were shorter than spliced introns (mean 1.8 kb versus 4.0 kb), had higher GC content (mean 52% versus 46%) and their host RNA showed higher nuclear enrichment (mean log_2_ fold change (FC) 1.3 versus −0.05; all *P* < 2.2 × 10^−16^, two-sided Welch’s *t*-test; Fig. 1c), consistent with previous findings in other cell types^16,24,35,66,67^ and suggesting sequence composition biases introns towards retention.

To investigate the localization of IR-RNAs at the single-molecule level, we applied smRNA fluorescence in situ hybridization (smRNA FISH) on 21 candidates (18 mRNAs and 3 lncRNAs) spanning diverse functional categories identified in Fig. 1b, including chromatin regulation, RNA metabolism, cell cycle, DNA damage response, genome maintenance and vesicle trafficking (Fig. 1d,e and Extended Data Fig. 1b). IR for some candidates was previously reported, although not necessarily the same intron^23,68–76^. Quantitative distribution analysis revealed three major localization patterns (Fig. 1e,f and Extended Data Fig. 1b). Class I transcripts (33%) were predominantly nuclear (≥70% of RNA molecules) in their IR form with broad distribution, including TERT, CENPT, FANCA, BRD8, RAD52, LINC00106 and MEG3. Class II (38%) showed intermediate nuclear localization (55–70% of RNA molecules), most displaying a broad distribution, consistent with substantial coexistence of IR- and spliced isoforms, including METTL3, EZH2, HDAC7, COG4, DDX17, LAMA5, TUG1 and TELO2. Class III (29%) exhibited lower nuclear abundance (<55% of RNA molecules) and predominant cytoplasmic localization of spliced isoform, with RI signals frequently, but not necessarily, at or near transcription sites, including TP53BP1, SFPQ, WEE1, DNMT3B, SON and PTBP1.

Together, this underscores two fates of IR-RNAs, a broadly mobile nuclear pool and a locally constrained population, which is indistinguishable from PIR values in compartment-resolved RNA-seq alone, suggesting alternative routes of post-transcriptional control. Interestingly, most nuclear-enriched IR-RNAs encode regulators of essential pathways, including centromere and kinetochore assembly (CENPT^77^), DNA repair (FANCA^78^), chromatin regulation (BRD8^79^), telomere maintenance (TERT^80^) and m^6^A RNA methylation (METTL3^81^), indicating IR as a spatial checkpoint.

### Transcriptional inhibition time course reveals long-lived IR-RNAs

**Fig. 2:**
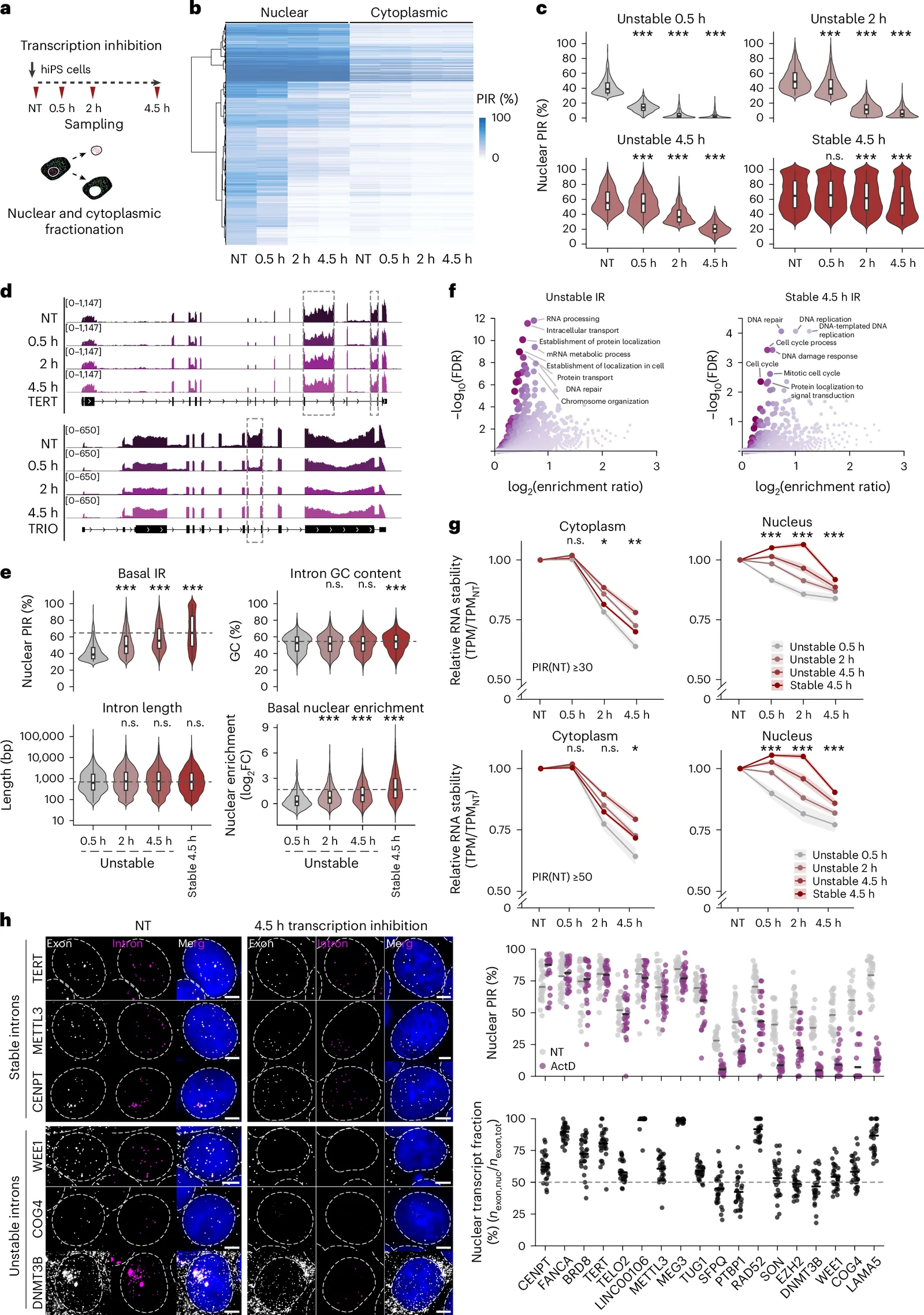
Transcriptional inhibition time course reveals a stable class of IR-RNAs. **a**, Schematic of the experimental approach. **b**, Heatmap of PIR across four timepoints after transcriptional inhibition in nuclear and cytoplasmic fractions. **c**, Violin plots of nuclear PIR across timepoints for introns grouped by IR stability (*n* = 1,031, 3,312, 899 and 3,869 introns; unstable 0.5 h, unstable 2 h, unstable 4.5 h, stable 4.5 h groups, respectively). n.s., not significant, ***P ≤ 0.001, evaluated by one-way ANOVA followed by Tukey’s HSD test versus NT. *P*_adj_ for 0.5 h, 2 h and 4.5 h versus NT, respectively: unstable 0.5 h, 3.19 × 10^−8^, 3.19 × 10^−8^, 3.19 × 10^−8^; unstable 2 h, *P*_adj _= 3.20 × 10^−10^, 3.20 × 10^−10^, 3.20 × 10^−10^; unstable 4.5 h, *P*_adj _= 1.65 × 10^−4^, 5.65 × 10^−9^, 5.65 × 10^−9^; stable 4.5 h, *P*_adj _= 3.26 × 10^−7^, 1.60 × 10^−8^. **d**, Poly(A)^+^ RNA-seq coverage across genes with stable IR (top) or unstable IR (bottom) across four timepoints. **e**, Violin plots of basal PIR, GC content and length of RIs grouped by IR stability as in **c** (with the same intron *n*), and of host RNA nuclear enrichment (log_2_(TPM_nuc_/TPM_cyto_)) for the corresponding transcript groups (*n* = 405, 1,391, 478 and 2,438 transcripts across increasing stability groups). n.s., not significant, ***P ≤ 0.001, evaluated by one-way ANOVA followed by Tukey’s HSD test versus unstable 0.5 h. Basal PIR, *P*_adj_ < 2.2 × 10^−16^ for unstable 2 h, unstable 4.5 h and stable 4.5 h; GC content, *P*_adj _= 1.71 × 10^−6^ for stable 4.5 h; host RNA nuclear enrichment, *P*_adj_ = 4.98 × 10^−8^, 3.51 × 10^−8^ and 3.51 × 10^−8^ for unstable 2 h, unstable 4.5 h and stable 4.5 h, respectively. **f**, GO analysis of stable and unstable IR-RNAs (circle size, gene count). **g**, Relative RNA stability compared with non-treated cells (TPM/TPM_NT_) in cytoplasmic (left) and nuclear (right) fractions for IR-RNAs grouped by IR stability and stratified by baseline PIR ≥ 30% (top; *n* = 368, 1,232, 422 and 1,675) or PIR ≥50% (bottom; *n* = 110, 749, 295 and 1,233), with group sizes reported in the same order as in **c**. *P < 0.05, **P ≤ 0.01, ***P ≤ 0.001, evaluated by two-sided Welch’s *t*-test stable 4.5 h versus unstable 0.5 h. PIR ≥30%, cytoplasm, *P*_adj _= 4.28 × 10^−2^, 3.41 × 10^−3^ at 2 h and 4.5 h, respectively; nucleus, *P*_adj _= 6.17 × 10^−29^, 2.46 × 10^−25^, 5.20 × 10^−4^ at 0.5 h, 2 h and 4.5 h, respectively. PIR ≥50%, cytoplasm, *P*_adj _= 4.31 × 10^−2^ at 4.5 h; nucleus, *P*_adj _= 7.71 × 10^−11^, 6.00 × 10^−10^, 1.78 × 10^−4^ at 0.5 h, 2 h and 4.5 h, respectively. Dots, mean; shadows, standard error of the mean. **h**, Representative smRNA FISH maximum intensity projections of stable and unstable IR-RNAs in non-treated cells and 4.5 h after transcriptional inhibition. Exon, white; intron, magenta; nucleus, blue; scale bar, 2 μm. Right: nuclear PIR and nuclear transcript fraction (%) (*n*_exon,nuc_/*n*_exon,total_) from smRNA FISH quantification. Dots, individual cells; horizontal lines, means; *n* = 30 cells, two independent stainings. Additional representative images and quantifications are shown in Extended Data Fig. 2c,d. For all panels, NT, non-treated cells. For **c** and **e**, midline, median; box limits, first and third quartiles; whiskers, most extreme values within 1.5× IQR of the hinges. Source data

**Extended Data Fig. 2:**
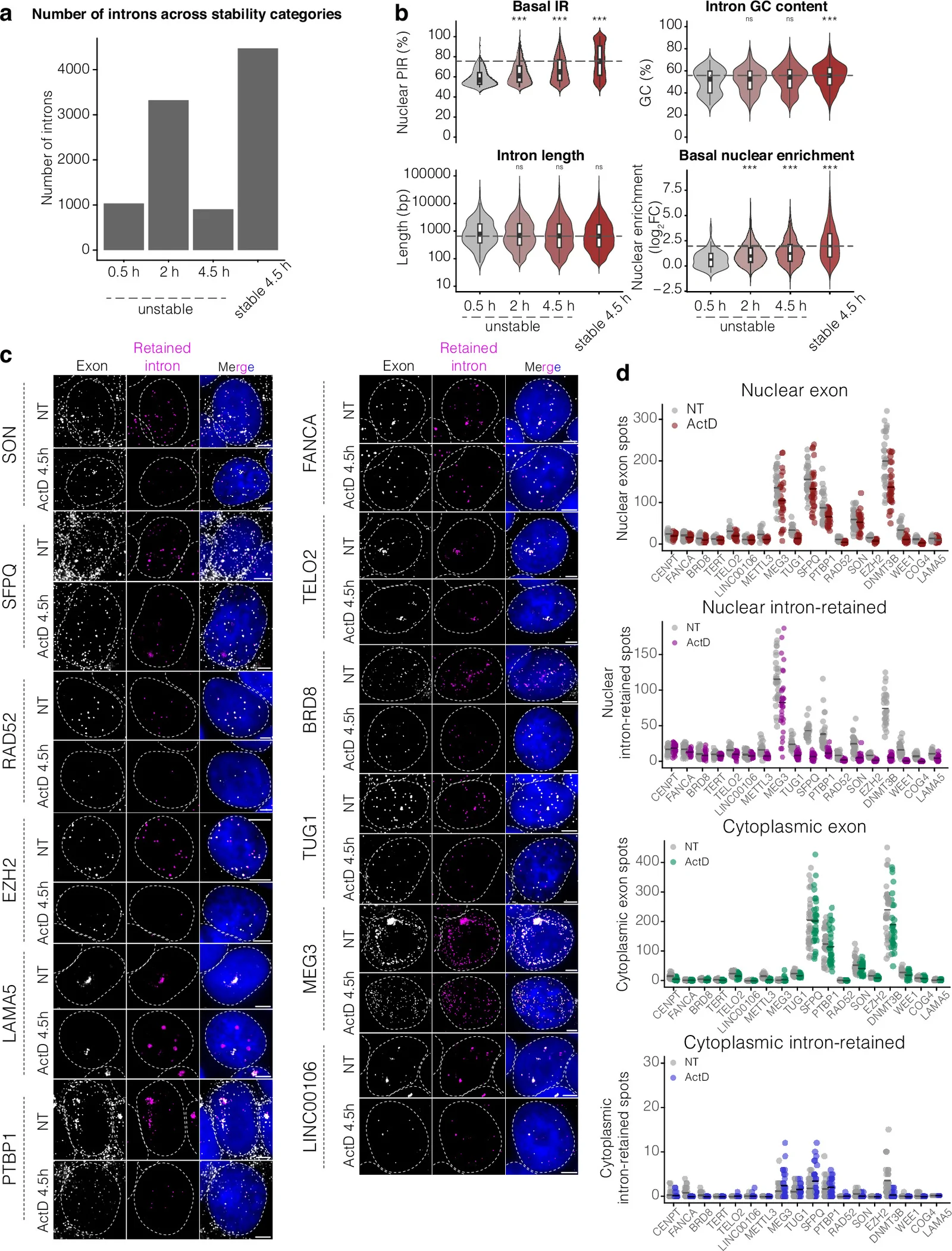
smRNA FISH analysis of IR stability in hiPSCs following transcriptional inhibition. **a**, Number of introns across different IR stability categories. **b**, Violin plot showing distribution of basal PIR, GC content and length of RIs, as well as host RNA nuclear enrichment [log_2_(TPM_nuc_/TPM_cyto_)] (*n* = 206, 1,547, 568 and 2,824 introns for the unstable 0.5 h, unstable 2 h, unstable 4.5 h and stable groups, respectively, and *n* = 122, 855, 350 and 1,919 transcripts, respectively). Midline, median; box limits, first and third quartiles; whiskers, most extreme values within 1.5 × IQR of the hinges. ns = not significant, ***P ≤ 0.001, evaluated by Tukey’s HSD test versus unstable 0.5 h. Basal PIR, *P*_adj_ = 2.80 × 10^−5^, 2.46 × 10^−11^, 4.06 × 10^−14^ for unstable 2 h, unstable 4.5 h, stable 4.5 h, respectively; GC content, *P*_adj_ = 1.93 × 10^−6^ for stable 4.5 h; host RNA nuclear enrichment, *P*_adj_ = 9.27 × 10^−4^, 7.05 × 10^−6^ and < 2.2 × 10^−16^ for unstable 2 h, unstable 4.5 h, stable 4.5 h, respectively. **c**, Representative maximum intensity projections of smRNA FISH images of hiPS cells treated with ActD for 4.5 h or non-treated cells (NT). Exon, white; intron, magenta; nucleus, blue, outlined with a dashed line; scale bar, 2 µm. **d**, Quantification of smRNA FISH for genes shown in **c** and Fig. 2h. *n* = 30 cells per gene, two independent stainings. Black line, mean values. Source numerical data are available in source data. Source Data

To determine how long RIs persist on subcellular timescales, we performed poly(A)^+^ RNA-seq on nuclear and cytoplasmic fractions after transcriptional inhibition with Actinomycin D (ActD) at 0.5, 2 and 4.5 h (Fig. 2a). The majority of IR events were enriched in nuclear RNA, as expected^16–18,35^, with a substantial fraction resolved between 0.5 h and 2 h, and a subset persisting throughout (Fig. 2b). RIs were classified as unstable at 0.5, 2 or 4.5 h, if PIR dropped below 50% of baseline at the respective timepoint, or stable if maintained throughout (Fig. 2b–d). Among 9,111 RIs, 1,031 (11.3%) resolved within 0.5 h, 3,312 (36.3%) by 2 h and 899 (9.9%) by 4.5 h, while 3,869 (42.5%) remained stable (Fig. 2c and Extended Data Fig. 2a). Stratification by localization class (Fig. 1e,f) revealed a correspondence between spatial pattern and kinetic stability: class I introns were predominantly stable (85.7%), class II mixed (37.5% stable) and class III were predominantly unstable (83.3%).

Stable RIs had higher GC content (53.7% versus 51.5–51.9% in unstable groups, *P*_adj_ < 1.3 × 10⁻^4^, Tukey’s honestly significant difference (HSD) test stable versus unstable groups), whereas intron length showed no substantial differences (Fig. 2e), indicating that sequence composition rather than length correlates with persistence. Baseline PIR and nuclear RNA enrichment both increased progressively with stability (Fig. 2e). We termed this stable class, defined by higher GC content, elevated baseline PIR and nuclear retention, long-lived RIs (LL-RIs), and the unstable group short-lived RIs (SL-RIs). GO analysis showed that LL-RIs are enriched in DNA repair, replication, cell cycle and mitosis-related pathways, whereas SL-RIs are enriched in RNA processing, metabolism and protein localization pathways (Fig. 2f), linking IR kinetics to gene function and indicating that genome maintenance and cell cycle pathways associate with the longest-lived IR.

Analysis of mean relative RNA levels across compartments showed that LL-IR-RNAs were, on average, more stable in the nucleus, with a decrease at 4.5 h (Fig. 2g). By contrast, groups with shorter IR persistence showed progressively lower average nuclear RNA stability. Cytoplasmic RNA levels declined faster than nuclear levels across all groups, most prominently for the unstable 0.5 h group, indicating partial uncoupling between nuclear and cytoplasmic RNA stability. These data indicate that temporal IR stability is associated with increased nuclear RNA persistence, while cytoplasmic RNA is subject to higher turnover.

Because the 30% PIR cutoff can include transcripts in which RIs represent a minority of the pool, we repeated the analysis at 50% PIR to focus on IR-dominant transcript populations. Trends in basal PIR, GC content, nuclear enrichment and nuclear decay were preserved, with GC content differences becoming sharper and increasing progressively across stability groups (mean 50.5%, 51.6%, 52.8%, 54.6%, *P*_adj _= 1.93 × 10^−6^, Tukey’s HSD test stable 4.5 h versus unstable 0.5 h; Extended Data Fig. 2b), reinforcing the link between GC content and IR stability, while nuclear decay of the unstable 0.5 h group became stronger (Fig. 2g). Increasing the PIR threshold therefore filtered out marginal events and sharpened the distinction between rapidly changing IR events and a kinetically distinct, long-lived IR population. We validated these results with smRNA FISH on 18 RIs (9 stable), confirming the persistence of LL-RIs after transcriptional inhibition, and rapid depletion of SL-RIs. Furthermore, stable RIs were predominantly found in broadly distributed RNAs, with high basal PIR and high nuclear transcript fraction (Fig. 2h and Extended Data Fig. 2c,d).

**Extended Data Fig. 3:**
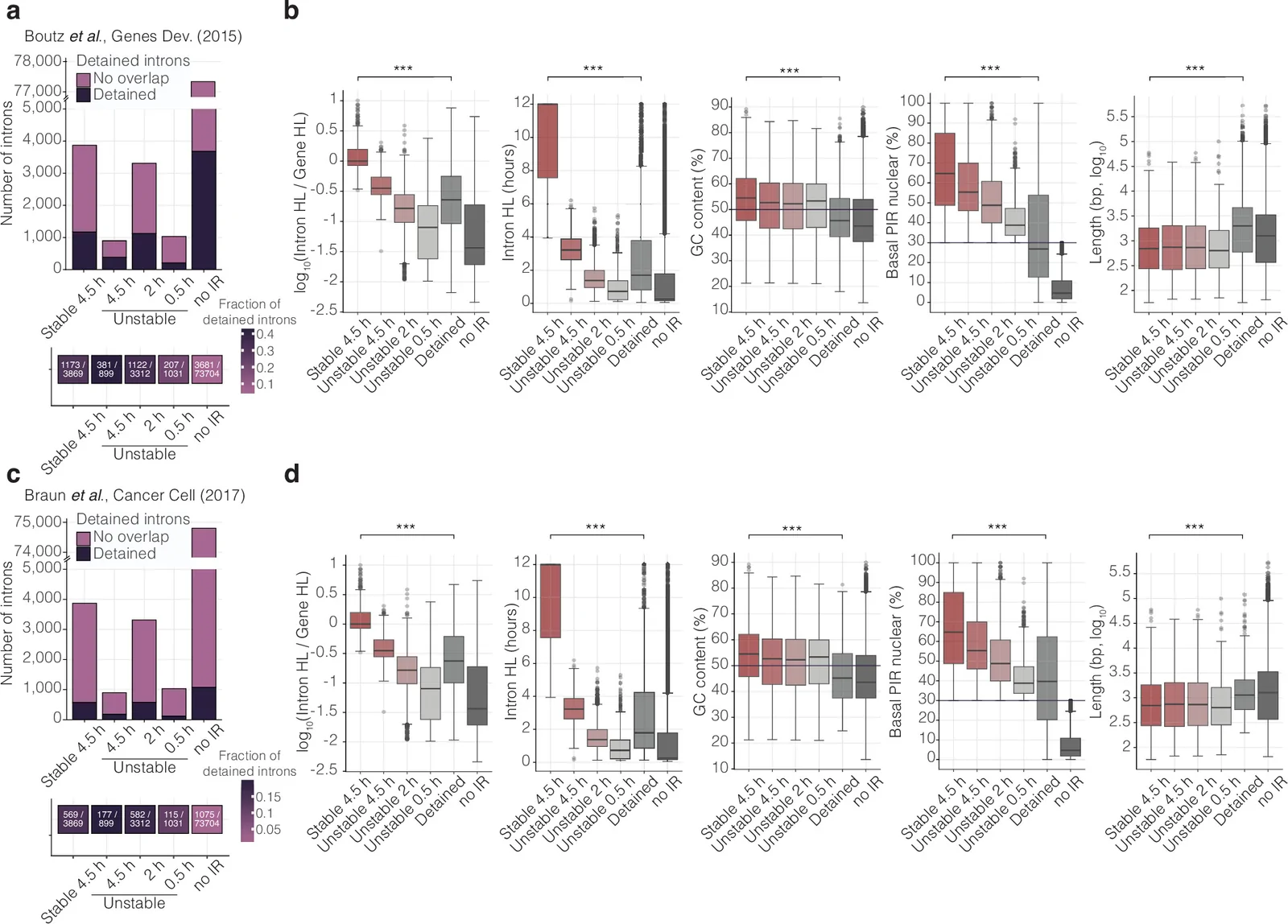
Overlap of IR stability groups with published detained intron datasets. **a**, Stacked bar plots showing the number of introns from the indicated IR stability groups that overlap with detained introns defined by Boutz et al. versus introns without overlap. Bottom, fraction of shared introns in each group. **b**, Boxplots showing host RNA nuclear enrichment [log_2_(TPM_nuc_/TPM_cyto_)], basal PIR, GC content, and intron length for the indicated IR stability groups, introns overlapping the Boutz detained intron set, and introns without IR (no IR). ***P ≤ 0.001, evaluated by one-way ANOVA followed by Tukey’s HSD test stable 4.5 h versus detained introns. **c**, Same as in **a**, but using the detained intron set defined by Braun et al. **d**, Same as in **b**, but for introns overlapping the Braun detained intron set. ***P ≤ 0.001, evaluated by one-way ANOVA followed by Tukey’s HSD test stable 4.5 h versus detained introns. For **b** and **d**, *P*_adj_ < 2.2 × 10^−16^ for all plotted features. Source numerical data are available in source data. Source Data

Because detained introns represent the best-characterized reference of nuclear polyadenylated IR-RNAs that can undergo post-transcriptional splicing, we compared the stability classes to detained introns reported by Boutz et al.^17^ and Braun et al.^18^. Annotated detained introns distributed across multiple stability groups (Extended Data Fig. 3a,c), with 42–56% having lower baseline PIR in our dataset and mapping to the no IR category, probably reflecting differences in both cell type and event definition. Among those that could be assigned to our IR stability groups (46–58%), 59–61% mapped to unstable groups, whereas 39–41% to the stable group. Only a minority of our stable introns overlapped annotated detained introns, corresponding to ~15% in the Braun dataset and ~30% in the Boutz dataset (Extended Data Fig. 3a,c).

In the work of Boutz et al., half-life (HL) was measured for six detained introns and averaged ~29 min (ref. ^17^). Consistent with this, annotated detained introns remained shorter-lived than the stable class (mean HL 3.2–3.4 h versus 9.99 h), had lower basal PIR (mean ~35–42% versus ~66%), greater intron length (mean log_10_(length) ~3.07–3.21 versus ~2.86) and lower GC content (mean ~46–47% versus ~54%; all *P*_adj_ < 2.2 × 10^−16^, Tukey’s HSD test; Extended Data Fig. 3b,d). Detained introns in the work of Boutz et al. were enriched in RNA processing, stress response, DNA damage and cell cycle regulation pathways^17^, consistent with the broad functional enrichment in Fig. 1b, whereas the long-lived class shows a more specific enrichment in mitosis, DNA repair and genome maintenance pathways. These comparisons place stable IR within the broader intron detention framework as a long-lived population.

Collectively, LL-RIs (42% of the IR pool) are GC-rich, nuclear-enriched, stable and linked to cell cycle, mitosis and DNA repair regulators, while SL-RIs predominate among RNA metabolism genes, suggesting that IR dynamics contribute to post-transcriptional control in a context-dependent manner.

### RIs are predictable from sequence features linked to RBP binding and are enriched for nuclear speckle-associated RBPs

**Fig. 3:**
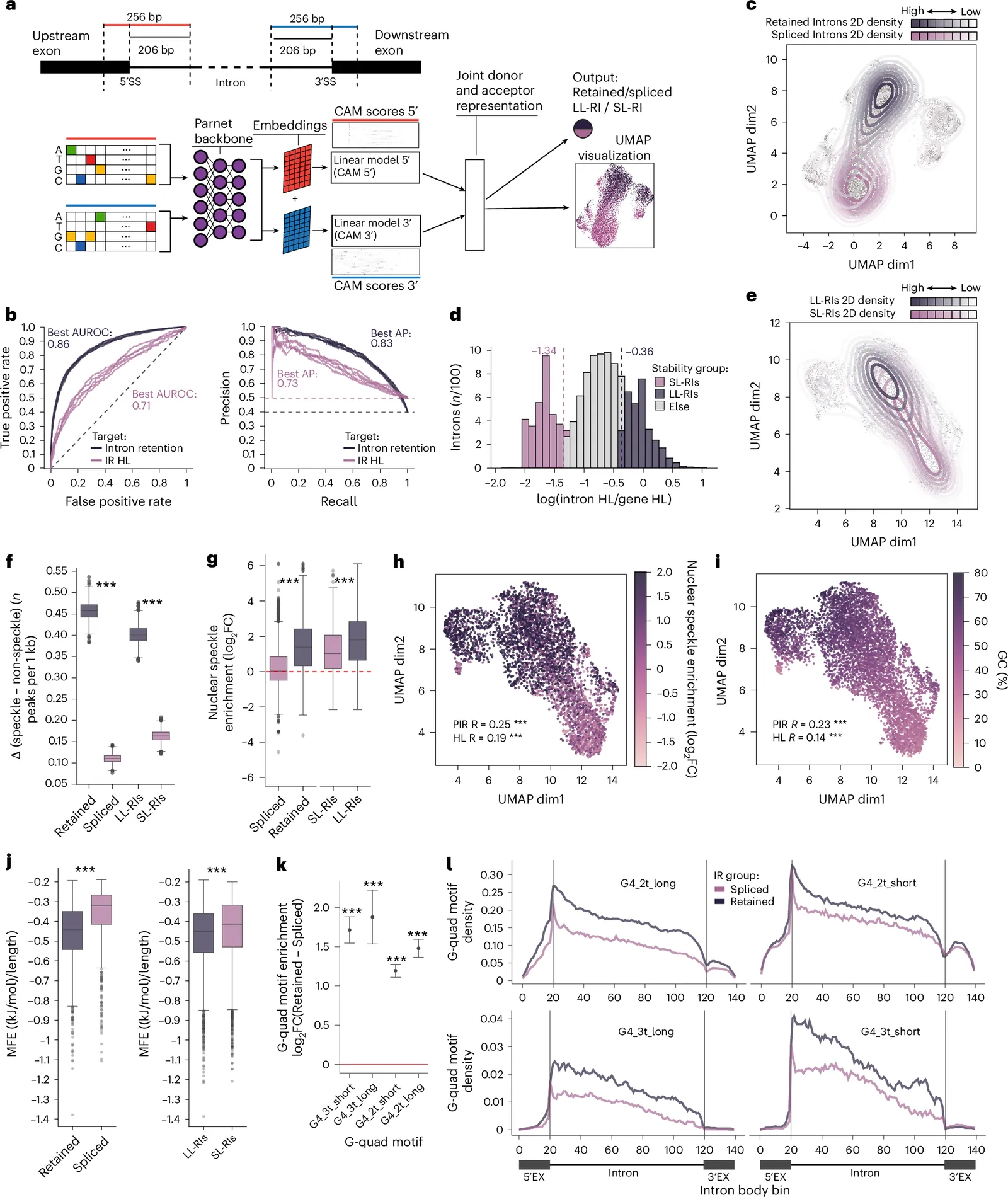
IR-RNAs are associated with nuclear speckles and enriched in G-quadruplexes. **a**, Schematic illustrating the process of Parnet’s fine-tuning to predict IR or IR HL. One-hot encoded sequences of intron termini are passed through the Parnet model to generate embeddings, which are then decomposed by the CAM modules assigning per-position importance score and a probability of a given intron being retained/stable. Two probabilities are then fused to generate the final prediction. **b**, Precision–recall (PRC; left) and receiver operating characteristic (ROC; right) curves across five folds of Parnet fine-tuning for IR (purple) and IR HL (pink) classification. Best-fold average precision (AP) and AUROC values are shown. **c**, UMAP projection of IR fine-tuned Parnet intron embeddings, where each point represents an intron and distances reflect learned feature similarity. Contour lines indicate kernel density estimates for the two IR classes. In the legends, dim stands for UMAP dimension. **d**, Distribution of gene-level normalized IR HL, with LL-RI and SL-RI cutoffs indicated. **e**, As in **c**, using IR HL fine-tuned embeddings; contours represent densities of IR HL classes defined in **d**. **f**, Boxplots showing the distribution of bootstrapped average deltas between nuclear speckle and non-nuclear speckle RBP peaks in the four intron classes defined in **d** and Extended Data Fig. 4a (*n* = 2,000 in all four cases). Midline, median; box limits, first and third quartiles; whiskers, most extreme values within 1.5× IQR of the hinges. LL-RIs versus SL-RIs, *P*_adj _= 3.57 × 10^−22^; retained versus spliced introns, *P*_adj _= 9.96 × 10^−43^, as estimated by two-way ANOVA. ***P ≤ 0.001. **g**, Boxplots showing the distribution of nuclear speckle enrichment across the four classes defined in **d** and Extended Data Fig. 4a. Box bounds are defined as in **f**. ***P ≤ 0.001, as estimated by Welch’s two-sided *t*-test. LL-RIs (*n* = 2,140) versus SL-RIs (*n* = 2,322), *P*_adj _= 4.30 × 10^−57^; retained (*n* = 5,867) versus spliced (n = 7,618) introns, *P*_adj_ = 2.2 × 10^−308^. Nuclear speckle enrichment data were derived from ref. ^74^. **h**, Same as **e**, but coloured by nuclear speckle enrichment of the intron hosting gene. Pearson’s *r* values between IR and IR HL, and colour-coded variables are reported on the plot. **i**, Same as in **e**, but introns coloured by GC content. **j**, Boxplots showing the distributions of length-normalized MFE estimates for introns subsampled from four classes defined in **d** and Extended Data Fig. 4a. ***P ≤ 0.001, Welch’s two-sided *t*-test. LL-RIs (*n* = 2,982) versus SL-RIs (n = 2,988), *P*_adj _= 2.42 × 10^−11^; retained (*n* = 734) versus spliced (n = 966), *P*_adj_ = 3.97 × 10^−40^. Box bounds are defined as in **f**. **k**, Dot plot showing the enrichment of four G4 motif counts. Points, log_2_ fold enrichment of motif-hit rates in retained (*n* = 9,111) relative to spliced (*n* = 11,131) introns; error bars, 95% confidence intervals for the log_2_ fold enrichment. ***P ≤ 0.001. Significance was estimated from the corresponding Poisson generalized linear model class coefficient (see‘G-quadruplex motif enrichment’ section in the Methods). *G4_3t_short, P*_adj _= 3.4 × 10^−80^; *G4_3t_long, P*_adj_ = 2.11 × 10^−22^; *G4_2t_short, P*_adj _= 1.26 × 10^−190^; *G4_2t_long, P*_adj_ = 3.13 × 10^−149^. **l**, Meta-profiles showing four G4 motif distributions along the intron body and flanking exons of retained (purple) and spliced (pink) introns as defined in Extended Data Fig. 4a. EX stands for exon and denotes the borders between the intron body and the flanking exons. Source data

**Extended Data Fig. 4:**
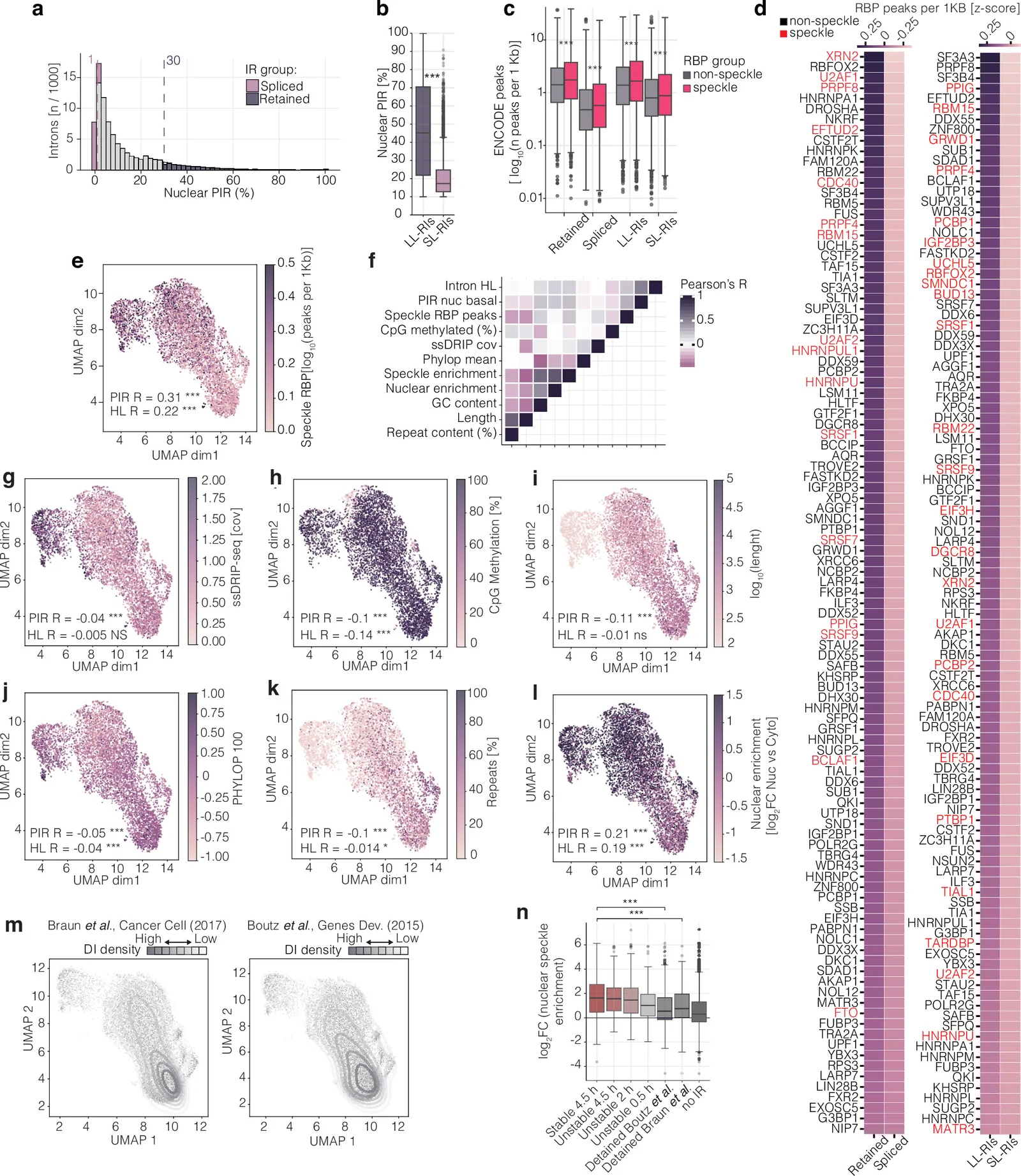
IR-RNAs associate with nuclear speckles. **a**, Histogram showing the distribution of PIR levels. Cutoffs for retained and spliced classes are labeled on the plot. **b**, Boxplots showing the distribution of PIR levels in IR HL classes as defined in Fig. 3d. Welch’s two-sided t-test (*n* = 3171 LL-RIs, *n* = 3171 SL-RIs). *P* = 2.2 × 10^−308^. ***P ≤ 0.001. **c**, Boxplots showing the distributions of nuclear speckle and other RBP peaks. Welch’s two-sided t-test (*n* = 5000 per group). Retained *P*_adj_ = 1.13 × 10^−14^; Spliced *P*_adj_ = 6.37 × 10^−4^; LL-RIs *P*_adj_ = 9.56 × 10^−25^; SL-RIs *P*_adj_ = 1.13 × 10^−10^. ***P ≤ 0.001. **d**, Heatmap showing the enrichment of ENCODE eCLIP RBP peaks in IR HL and IR intron classes. RBPs associated with nuclear speckles are colored in red. **e**, UMAP reduction of Parnet intron embeddings fine-tuned on IR HL. Each point is an intron and is coloured by the average of length-normalized ENCORE speckle RBP peaks. Pearson’s two-sided correlation tests between the color-coded covariate and IR *n* = 79,436, *P*_adj_ = 2.2 × 10^−308^; IR HL *n* = 25,354, *P*_adj_ = 9.06 × 10^−283^. ***P ≤ 0.001. **f**, Correlation plot showing Pearson’s R for pairs of variables on X and Y. **g-l**, Same as **e**, but colored by ssDRIP-seq coverage (**g**; *P*_ad__j_(IR) = 1.85 × 10^−30^); CpG methylation (**h**; *P*_adj_(IR) = 6.44 × 10^−166^, *P*_adj_(HL) = 9.2 × 10^−102^); intron length (**i**; *P*_adj_(IR) = 5.65 × 10^−211^); phyloP (**j**; *P*_adj_(IR) = 6.94 × 10^−44^, *P*_adj_(HL) = 3.49 × 10^−9^); repeats (**k**; *P*_adj_(IR) = 1.03 × 10^−173^, *P*_adj_(HL) = 0.0302); nuclear enrichment (**l**; *P*_adj_(IR) = 2.2 × 10^−308^, *P*_adj_(HL) = 7.03 × 10^−209^). ns = not significant, *P < 0.05, ***P ≤ 0.001. **m**, Projections of Parnet embeddings of the two sets of detained introns defined by Braun et al. 2017 (left) and Boutz et al. 2015 (right) onto the UMAP of the introns from this study. Contour lines show kernel density estimates for the two sets of detained introns. **n**, Boxplots showing host RNA nuclear speckle enrichment for the IR stability groups, introns overlapping the two detained intron sets, and introns without IR (no IR). Tukey’s HSD *P*_adj_ < 2.2 × 10^−16^ for both comparisons; *n*(stable 4.5 h) = 2300, *n*(Boutz et al.) = 4952, *n*(Braun et al.) = 1972. ***P ≤ 0.001. For **b**, **c**, **n**, midline, median; box limits, first and third quartiles; whiskers, most extreme values within 1.5 × IQR of the hinges. Source numerical data are available in source data. Source Data

We next sought to unbiasedly characterize sequence features of RIs and LL-RIs using Parnet^82^, an experimentally grounded in-house RNA foundation model trained on the full ENCORE RBP eCLIP dataset. We first fine-tuned Parnet to classify retained versus non-retained introns (Methods), independent of HL (Fig. 3a and Extended Data Fig. 4a). The model distinguished retained from non-retained introns with high accuracy (area under receiver operating characteristic curve and average precision 86% and 83%, respectively; henceforth, AUROC and AP, respectively; Fig. 3b) and the two classes separated in embedding space (Fig. 3c), indicating that IR is encoded in distinct sequence features, capturing RNA–RBP interaction potential and local sequence information.

Although LL-RIs show higher basal retention than unstable introns (Fig. 2e and Extended Data Fig. 4b), we next fine-tuned Parnet to distinguish LL-RIs from SL-RIs (Methods), defined by the lower log(IR HL <−1.34) and upper log(IR HL ≥−0.36) quartiles of the HL distribution (Methods; Fig. 3d). Despite the smaller, more complex training set, Parnet achieved clear separation between SL- and LL-RIs (Fig. 3e) and performed well above random (AUROC 71%, AP 73%; Fig. 3b), indicating that sequence features contribute to IR stability.

Independent analysis revealed an enrichment of nuclear speckle-associated RBP binding sites as a hallmark of RIs overall, particularly of LL-RIs (Fig. 3f and Extended Data Fig. 4c), which had increased ENCODE eCLIP RBP peaks compared with efficiently spliced introns and SL-RIs (Methods; Extended Data Fig. 4d). We interpreted the learned embeddings to assess whether the fine-tuned foundation model captures these features. Projecting SL- and LL-RI representations and colouring by nuclear speckle-associated RBP peaks revealed a clear gradient: LL-RIs clustered where ENCODE nuclear speckle RBP binding was higher (Extended Data Fig. 4e). Thus, the model encodes intronic features linked to nuclear speckle association and discriminates SL- from LL-RIs accordingly. Consistently, assay of reverse transcription-based RBP binding-site sequencing (ARTR-seq) data^74^, approximating RNA enrichment by tethering reverse transcriptase to nuclear speckle markers, showed a similar LL-RI-to-SL-RI gradient and RI enrichment in nuclear speckle-associated fractions (Fig. 3g,h). Together, these results reinforce the association between nuclear speckles and intron fate.

In line with this, Parnet embeddings revealed a strong link between RIs, LL-RIs and GC content (Fig. 3i and Extended Data Fig. 4f), confirming their GC richness (Fig. 2e). This is consistent with proximity- and antibody-guided RNA-labelling studies showing that RIs enriched with nuclear speckle markers tend to be shorter and GC-rich^60,74^ (Extended Data Fig. 4f). Because GC-rich sequences can promote R-loop formation and transcriptional pausing^83^, we analysed single-strand DNA ligation-based DNA:RNA immunoprecipitation sequencing (ssDRIP-seq, a method for genome-wide detection of R-loops) data from the same cell line^84^. While R-loop peaks correlated with GC content, nuclear speckle enrichment and localization, we found no direct relationship between R-loops and IR or IR HL (Extended Data Fig. 4f,g). Bisulfite sequencing^84^ showed that most introns were highly methylated, except for a small group of short, conserved, demethylated first introns overlapping R-loops^85^ (Extended Data Fig. 4h–j). Intron length and repeat content showed negative correlation with basal PIR, whereas association with IR HL approached non-significance (Extended Data Fig. 4f,i,k). As expected, both basal PIR and IR HL correlated with the nuclear enrichment of the host gene (Extended Data Fig. 4f,l). Parnet embeddings revealed that annotated detained introns^17,18^ mostly overlapped with SL-RIs and had lower ARTR-seq nuclear speckle enrichment^74^ (mean log_2_FC 0.8 versus 1.6, respectively, *P* < 2.2 × 10^−16^, Tukey’s HSD test; Extended Data Fig. 4m,n).

**Extended Data Fig. 5:**
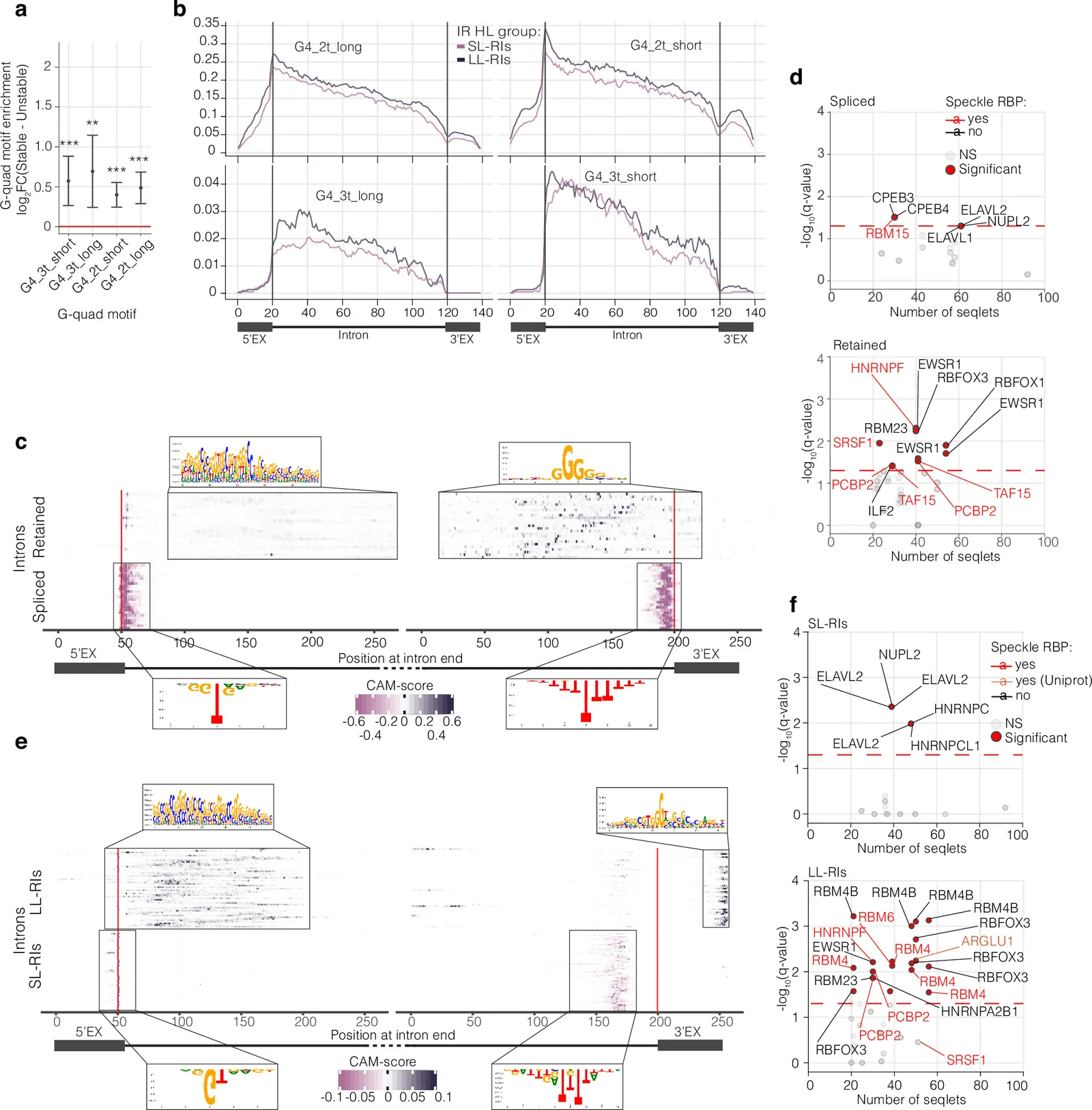
IR-RNAs are enriched in G-quadruplexes. **a**, Dot plot showing the enrichment of four G-quadruplex motif counts. Points show log2 fold enrichment of motif-hit rates in LL- relative to SL-RIs (*n* = 3171 LL-RIs and SL-RIs); error bars indicate 95% confidence intervals for the log2 fold enrichment. Statistical significance was estimated from the corresponding Poisson GLM class coefficient (*Methods - G-quadruplex motif enrichment*). G4_3t_short, *P*_adj_ = 3.53 × 10^−4^; G4_3t_long, *P*_adj_ = 2.6 × 10^−3^; G4_2t_short, *P*_adj_ = 1.46 × 10^−6^; G4_2t_long, *P*_adj_ = 2.58 × 10^−6^. *P < 0.05, **P ≤ 0.01, ***P ≤ 0.001. **b**, Meta profiles showing the distribution of four G-quadruplex motifs along the intron body and flanking exons within LL- (purple) and SL- (pink) RIs. **c**, Class Attribution Maps (CAMs) of Parnet fine-tuned on IR. Per-base class attribution scores for 256 bases on each end of the intron (X) are plotted for top 25 predicted introns in each class (Y). Top motifs extracted by tf-modisco for each intron’s end are reported above (Positive-Retained) and below (Negative-Spliced) (*Methods – Parnet fine-tuning and Class Activation Mapping (CAM) feature importance*). **d**, Results of TomTom analysis of motifs extracted from IR CAMs by tf-modisco. Each dot corresponds to a CAM-derived motif, with TomTom one-sided motif-comparison q-values (probability that a random motif would align to the query motif) shown on Y as -log10(q-value) and the number of motif seqlets (n) shown on X; q-values and n are reported in the *Source Data*. Up to three RBP motifs can be assigned to each input motif. Nuclear speckle RBPs are coloured in red. **e**, Same as **c**, but for Parnet fine-tuned on IR HLs. **f**, Same as **d**, but the motifs were extracted from the IR HL model CAMs (*Methods – Attribution scores interpretation*). Source numerical data are available in source data. Source Data

We next asked whether the GC richness of LL-IRs reflects enhanced RNA structure. LL-RIs displayed significantly lower length-normalized predicted minimum free energy (MFE) than unstable or spliced introns, indicating greater structural stability (Fig. 3j). Among structural elements, G-quadruplexes (G4s), which modulate pre-mRNA processing^86,87^ and accumulate near weak splice sites^86^, were ~3-fold enriched in RIs (Fig. 3k). For all motif categories (Methods), meta-profiles revealed a preferential G4 localization near the 5′ of RIs, declining towards the intron body and 3′ end (Fig. 3l). Two G4 motifs showed a secondary enrichment near the 3′ end, in proximity to branch site. G4 motifs were modestly enriched in LL-RIs (~1.5-fold) while preserving positional bias (Extended Data Fig. 5a,b).

Attribution analyses (Methods) identified additional predictive motifs: canonical 5′ splice site and 3′ polypyrimidine tract motifs, hallmarks of efficient splicing^88–90^ (Extended Data Fig. 5c), were depleted in RIs, consistent with previous studies^91^, suggesting that weakened splice signals and local structures jointly promote IR. Attribution maps also highlighted RBP binding sites^92^: positive attributions in RIs included RBFOX1/3, ILF2, EWSR1, TAF15, RBM23, PCBP2, HNRNPF and SRSF1; the latter four localized to nuclear speckles^93^ (Extended Data Fig. 5d, bottom). Negative attributions, enriched in efficiently spliced introns, identified six significant matches: CPEB3/4, ELAVL1/2, NUPL2 and RBM15 (Extended Data Fig. 5d, top). All except NUPL2 are established regulators of alternative splicing^94,95^; NUPL2, a nucleoporin-like factor, links late splicing to mRNP export^96^.

Compared with SL-RIs, LL-RIs showed weaker canonical 5′ splice sites, stronger GC-rich enrichment at the 5′ end, and overrepresented motifs for nuclear speckle-localized RBPs: RBM4/RBM4B, which bind G-rich sequences such as G4s, and PCBP2, ARGLU1 and HNRNPF, known alternative splicing regulators^97–105^ (Extended Data Fig. 5e,f).

Together, these analyses show that IR and stability are encoded in distinct sequence and structural features. RIs display weakened splice sites, G4 motif enrichment and splice silencing elements characterized by RBP motifs for SRSF1 or HNRNPF, while LL-IRs are further defined by GC-rich, structure-prone sequences and nuclear speckle-associated RBP interactions, suggesting that intron longevity arises from the interplay of *cis*-encoded features, RBP associations and spatial environment.

### IR-RNAs localize to a distinct niche at the periphery of nuclear speckles

**Fig. 4:**
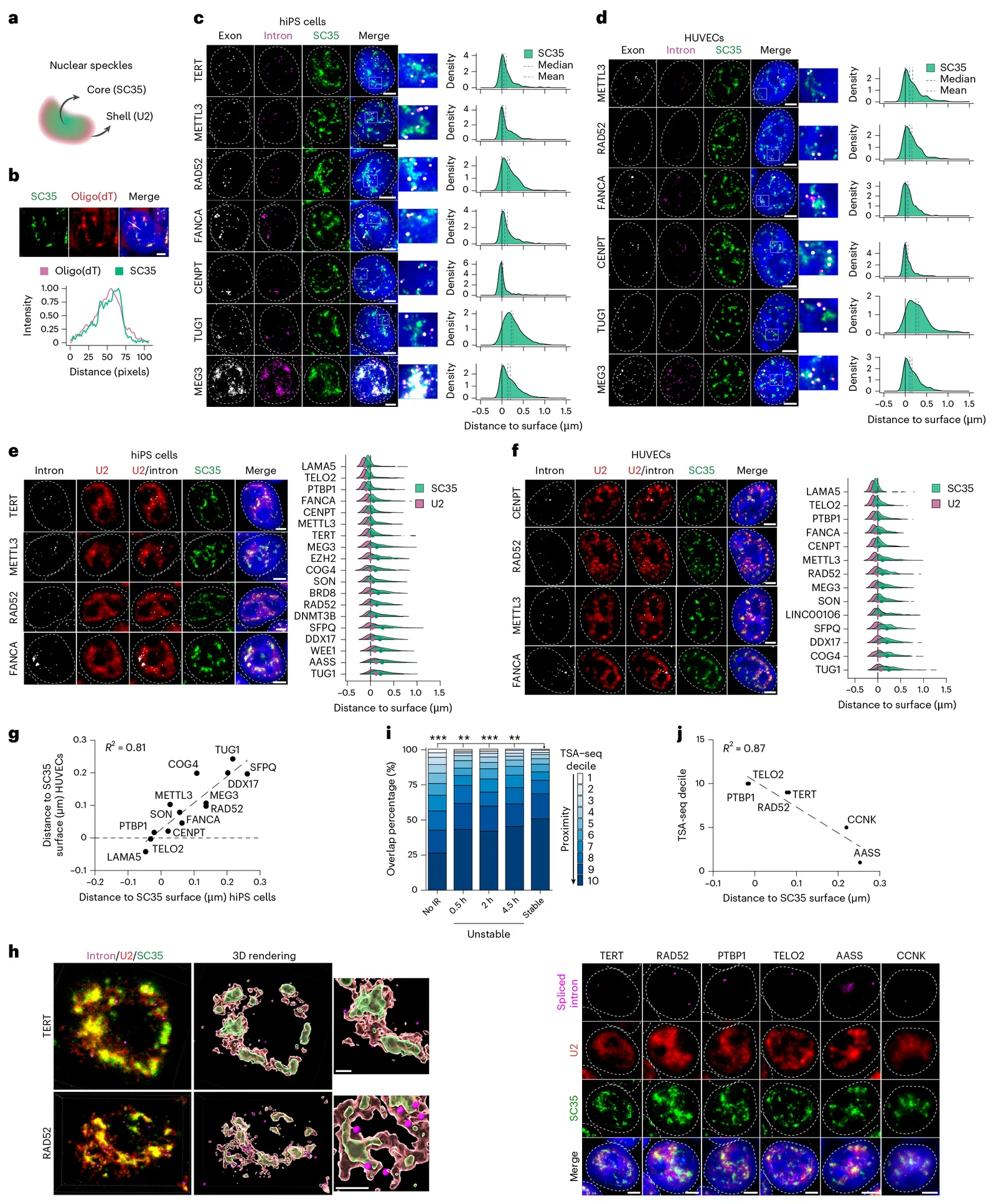
IR-RNAs localize at the nuclear speckle periphery. **a**, Schematic illustrating nuclear speckle architecture. **b**, Top: oligo(dT) smRNA FISH (red) and SC35 IF (green) maximum intensity projections. Nucleus, blue; scale bar, 2 µm. Bottom: co-localization plot of oligo(dT) and SC35 fluorescence intensity along the line indicated in the maximum intensity projection. Four independent stainings. **c**,**d**, Left: maximum intensity projections of exon (white) and intron (magenta) smRNA FISH and SC35 IF (green) in hiPS cells (**c**) and HUVECs (**d**). Nucleus, blue; scale bar, 2 µm. Right: density plots of distances (µm) to the surface of the SC35-positive nuclear speckle core for individual IR-RNA molecules. Positive values indicate molecules outside the segmented surface, negative values indicate the inward distance of RNA molecules located within the segmented surface. Median, dashed blue line; mean, dashed grey line. Three independent stainings. **e**,**f**, Left: maximum intensity projections of intron (white) and U2 (red) smRNA FISH combined with SC35 IF (green) in hiPS cells (**e**) and HUVECs (**f**). Nucleus, blue; scale bar, 2 µm. Right: density plots of IR-RNA distances (µm) to the nuclear speckle surface. Distances were computed to the segmented SC35 or U2 surface; positive values indicate molecules outside the segmented surface, negative values indicate the distances of RNA molecules inside the segmented surface relative to the boundary. Dots, median. Three independent stainings. Additional representative images are provided in Extended Data Figs. 6a and 7a. **g**, Correlation between median distances (µm) of IR-RNAs to nuclear speckles in hiPS cells and HUVECs. *n* = 13 IR-RNAs. Pearson’s *r* = 0.90, *R*^2^ = 0.81, *P* = 2.82 × 10^−5^. **h**, Maximum intensity projections (left) and 3D rendering (right) of smRNA FISH and SC35 IF using STED microscopy in hiPS cells. Intron, magenta; U2, red; SC35, green; scale bar, 1 µm. **i**, Stacked bar plot showing distribution of DNA loci of transcripts grouped by IR status and IR stability with respect to their proximity to nuclear speckles assessed by TSA-seq^109^. **P ≤ 0.01, ***P ≤ 0.001, Fisher’s exact test. *P*_adj_ versus stable IR-RNAs: unstable 0.5 h, 7.63 × 10^−3^; unstable 2 h, 4.63 × 10^−7^; unstable 4.5 h, 7.58 × 10^−3^; no IR, 3.28 × 10^−91^. **j**, Top: correlation between TSA-seq score and the median distances of transcription sites to the nuclear speckle surface obtained by smRNA FISH (*n* = 6 genes). Pearson’s *r* = −0.94, *R*^2^ = 0.87, *P* = 6.2 × 10^−3^. Bottom: maximum intensity projections of the efficiently spliced intron (magenta) and U2 (red) smRNA FISH combined with SC35 IF (green). Nucleus, blue; scale bar, 2 µm. Three independent stainings. Source data

Nuclear speckles have a role in nuclear RNA retention^38,41,42^, and proximity labelling showed that IR-RNAs associate with nuclear speckle markers^60^. However, the limited spatial resolution of proximity-based biotinylation, constrained by diffusion of reactive intermediates and the nanoscale organization of nuclear subdomains, leaves unclear how IR-RNAs are organized relative to nuclear speckles, how spatially constrained these associations are, and how their association relates to the internal core and shell architecture of nuclear speckles^106,107^ (Fig. 4a). To address this, we employed high-resolution microscopy combining immunofluorescence (IF) for SC35 (SRSF2) to detect nuclear speckle core and U2 small nuclear RNA (snRNA) smRNA FISH to label the shell, and computed shortest distances of each IR-RNA spot across an IR-RNA panel spanning different stability categories to the segmented surfaces of the SC35-positive nuclear speckle core and the U2-positive nuclear speckle shell (Methods).

**Extended Data Fig. 6:**
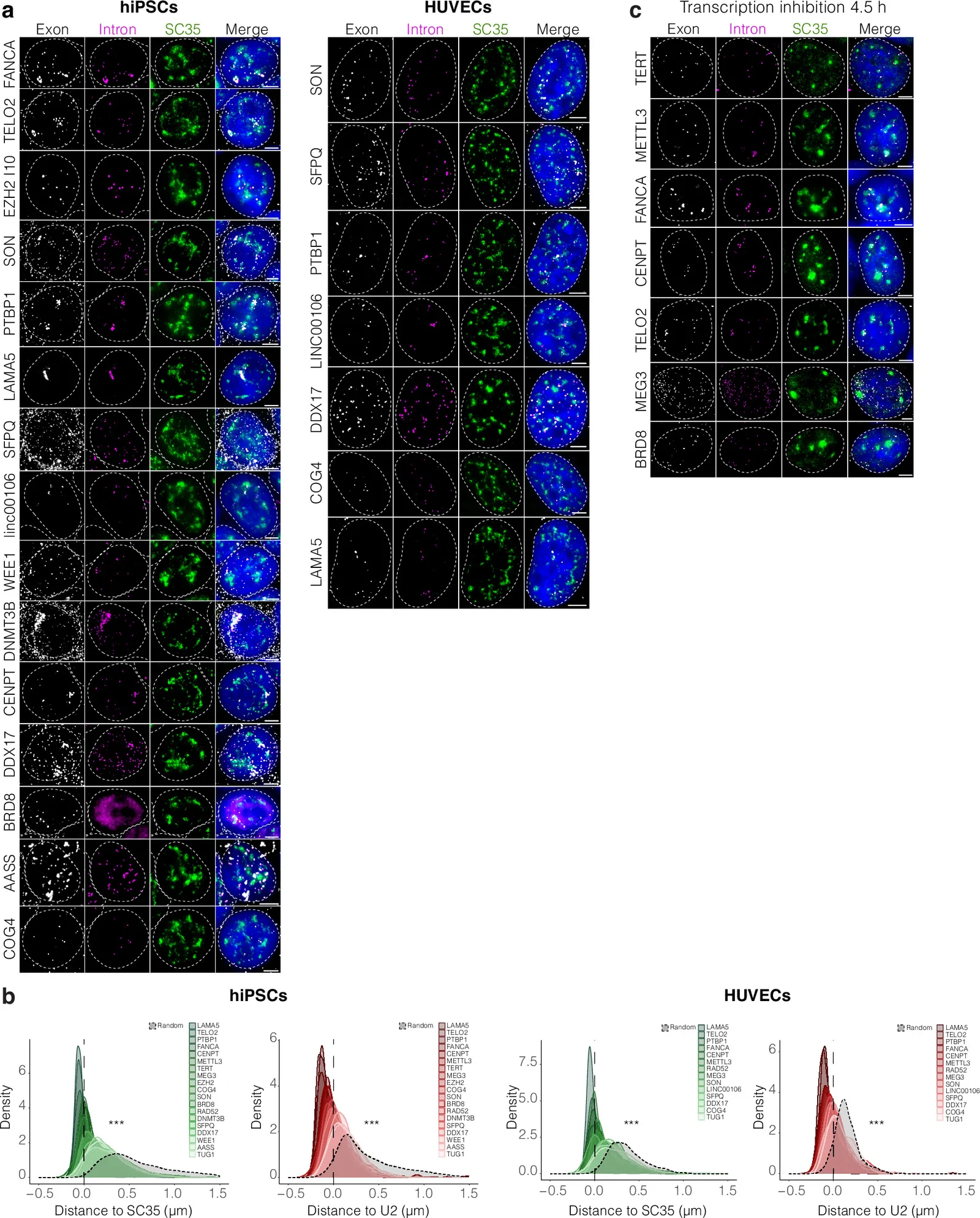
Subcellular localization of IR-RNAs relative to nuclear speckles in hiPSCs and HUVECs. **a**, Maximum intensity projections of smRNA FISH and SC35 IF in hiPSCs (left) and HUVECs (right). Exon, white; intron, magenta; SC35, green; nucleus, blue, outlined with a dashed line; scale bar, 2 µm. **b**, Density plots of the distance (µm) of IR-RNA molecules and random spots to nuclear speckle surface. Distances were computed to the segmented U2 and SC35 boundary for hiPSCs (left) and HUVECs (right). ***P ≤ 0.001, as evaluated by one-sided KS test, random spots versus individual IR-RNAs. In hiPSCs, *P*_adj_ < 2.2 × 10^−16^ for all RNAs for distances to SC35 and U2. In HUVECs, distance to SC35, *P*_adj_ < 2.2 × 10^−16^ for all RNAs except TUG1 (*P*_adj_ = 1.63 × 10^−12^) and COG4 (*P*_adj_ = 2.62 × 10^−9^); distance to U2, *P*_adj_ < 2.2 × 10^−16^ for all RNAs except COG4 (*P*_adj_ = 1.60 × 10^−11^). *n* IR-RNA molecules: hiPSCs, TELO2 718, PTBP1 687, CENPT 1,144, SON 1,308, MEG3 2,579, DDX17 2,977, AASS 2,830, LAMA5 397, FANCA 658, BRD8 993, METTL3 445, DNMT3B 725, WEE1 977, TUG1 824, RAD52 490, EZH2 538, SFPQ 877, TERT 184, COG4 125; HUVECs, MEG3 727, PTBP1 353, LAMA5 137, SON 371, CENPT 176, TELO2 150, FANCA 176, DDX17 879, METTL3 311, LINC00106 151, SFPQ 416, RAD52 117, TUG1 234, COG4 104. Random nulls were generated from 10 nuclei per target (hiPSCs: *n* = 9,267 SC35 and 9,269 U2 random spots; HUVECs: *n* = 2,445 SC35 and 2,445 U2 random spots). **c**, Maximum intensity projections of smRNA FISH in hiPSCs treated with ActD for 4.5 h. Channels are the same as in **a**. Scale bar, 2 µm. Source numerical data are available in source data. Source Data

Oligo(dT) smRNA FISH and SC35 IF showed global poly(A)^+^ RNAs within nuclear speckles, as expected^41,42^ (Fig. 4b). By contrast, IR-RNAs were predominantly excluded from the SC35-positive nuclear speckle core and positioned close to its outer surface, with median nearest distances to its outer surface ranging from −0.0470 to 0.261 µm (median across RNAs, 0.123 µm; Fig. 4c and Extended Data Fig. 6a). This positioning was non-random; all IR-RNAs were significantly shifted towards the core surface compared with randomly generated nuclear spots (one-sided Kolmogorov–Smirnov test, hereafter KS test; *P*_adj_ < 2.2 × 10^−16^; Extended Data Fig. 6b), with median distance shifts from −0.565 to −0.257 µm and strong enrichment at ≤0.1 µm from the core surface relative to random spots (median across RNAs, 16.4-fold; range, 7.9–32.4-fold; Supplementary Table 1). This indicates that IR-RNAs predominantly localize at the nuclear speckle periphery outside the SC35-positive core. To determine whether this distribution is conserved across cell types, we analysed HUVECs, which preserve native nuclear architecture. IR-RNAs showed the same peripheral positioning relative to the SC35-positive core (median distances from −0.0420 to 0.243 µm; median across RNAs, 0.0885 µm; Fig. 4d and Extended Data Fig. 6a), and were significantly shifted towards the core surface compared with random spots (one-sided KS test; all *P*_adj _< 4.3 × 10^−9^; Extended Data Fig. 6b), with median shifts from −0.370 to −0.085 µm and robust enrichment at ≤0.1 µm from the core surface (median across RNAs, 9.30-fold; range, 4.53–17.3-fold; Supplementary Table 1).

**Extended Data Fig. 7:**
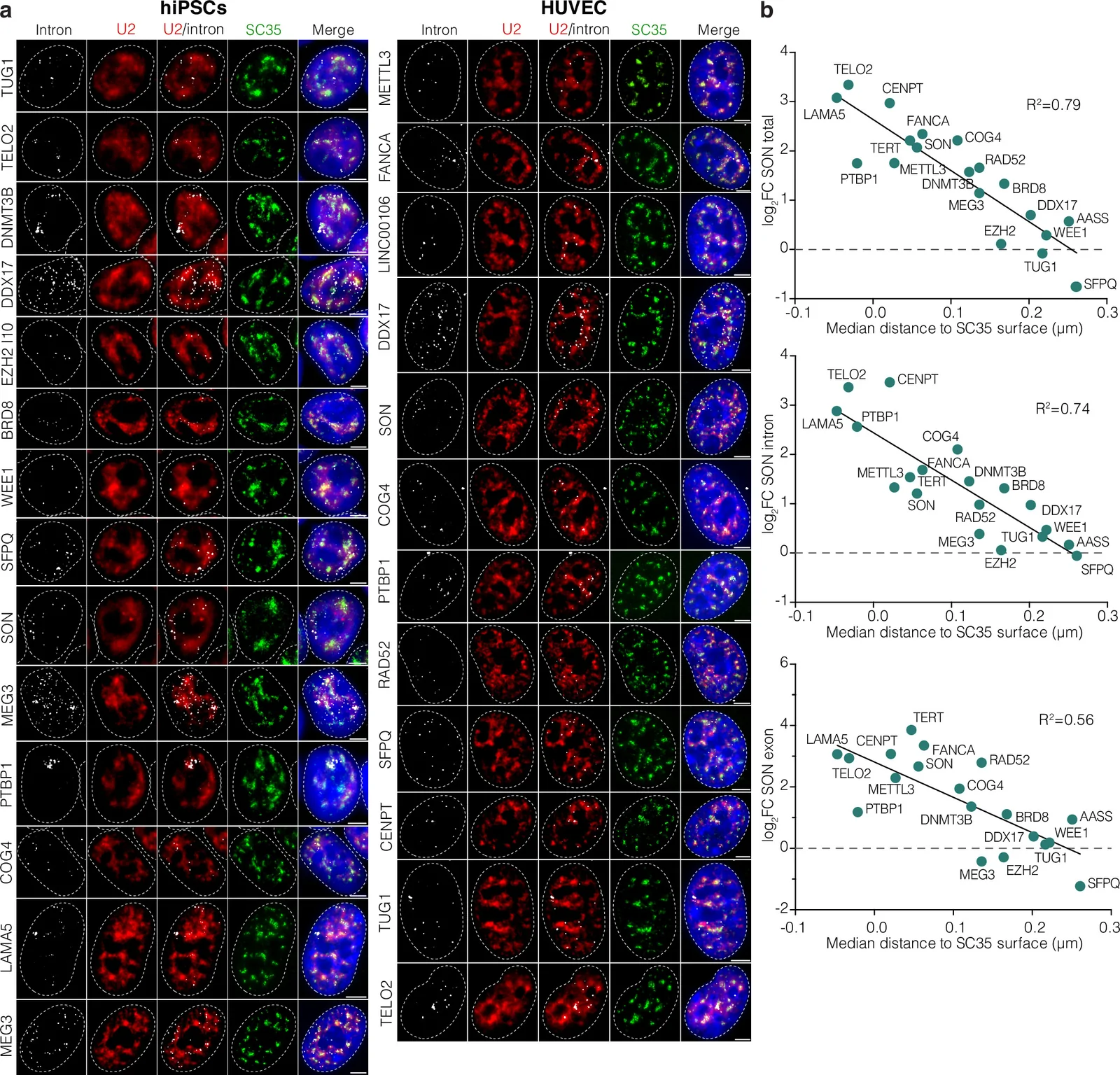
IR-RNAs localize to the outer shell of nuclear speckles in hiPSCs and HUVECs. **a**, Maximum intensity projections of smRNA FISH and SC35 IF in hiPSCs. Intron white; U2, red; SC35, green; nucleus, blue, outlined with a dashed line; scale bar, 2 µm. **b**, Correlation of SON log_2_ fold change of the indicated RNAs (derived from Wu et al., 2024^74^) and their median distances to the SC35-positive core surface (µm). Data are shown separately for total RNA (top), intron regions (middle), and exon regions (bottom). For total RNA, Pearson’s *r* = −0.89, *R*^2^ = 0.79, *P*_adj_ = 9.97 × 10^−7^; for introns, Pearson’s *r* = −0.86, *R*^2^ = 0.74, *P*_adj_ = 3.98 × 10^−6^; for exon regions, Pearson’s *r* = −0.75, *R*^2^ = 0.56, *P*_adj_ = 2.15 × 10^−4^. *n* = 19 RNAs. Source numerical data are available in source data. Source Data

Relative to the U2 shell, IR-RNAs predominantly localized close to the shell–nucleoplasm interface in both hiPS cells (median distances to the outer surface of the U2 shell ranging from −0.117 to 0.124 µm, median across RNAs, −0.002 µm) and HUVECs (median distances to the outer surface of the U2 shell ranging from −0.0980 to 0.0640 µm, median across RNAs, −0.0185 µm; Fig. 4e,f and Extended Data Fig. 7a). IR-RNAs were significantly shifted towards the U2 shell compared with random spots in hiPS cells (one-sided KS test; *P*_adj _< 2.2 × 10^−16^), with median shifts ranging from −0.394 to −0.154 µm and a median 4.05-fold enrichment at ≤0.1 µm from the shell surface across RNAs (range, 2.39–5.07-fold; Extended Data Fig. 6b and Supplementary Table 1). HUVECs showed the same pattern, with a significant shift towards the U2 shell compared with random spots and an enrichment at ≤0.1 µm from the shell surface (one-sided KS test; *P*_adj_ < 1.6 × 10^−11^; Extended Data Fig. 6b and Supplementary Table 1). Although IR-RNAs were closer to nuclear speckles than expected under a random null model, the effect size was heterogeneous, consistent with a graded spatial relationship. IR-RNAs with the largest proximity shifts clustered tightly at the nuclear speckle periphery, whereas more distal transcripts showed correspondingly smaller shifts relative to random. Importantly, nuclear speckle proximity was strongly correlated between hiPS cells and HUVECs (Pearson’s *r* = 0.90, *P* = 2.82 × 10^−5^; Fig. 4g), supporting conserved spatial organization across cell types.

To determine the spatial organization beyond the diffraction limit, we employed stimulated emission depletion (STED) super-resolution microscopy on TERT and RAD52 RIs, which showed with greater precision that they localize immediately adjacent to and largely outside the nuclear speckle core, reinforcing juxta-speckle localization (Fig. 4h).

IR-RNAs frequently localized around multiple nuclear speckles, consistent with diffusion-based distribution. Transcriptional inhibition, which induces poly(A)^+^ RNA storage within nuclear speckles^42^ and rounded nuclear speckle morphology^108^ (Extended Data Fig. 6c), did not disrupt the peripheral association of most IR-RNAs, indicating that their peripheral localization is stable and independent of transcription or intact nuclear speckle morphology, further distinguishing poly(A)^+^ IR-RNAs from global poly(A)^+^ RNAs.

Collectively, IR-RNAs localize at the nuclear speckle periphery, largely outside the SC35-positive core at the U2 shell–nucleoplasm interface, and frequently along the boundaries of multiple nuclear speckles, in a pattern that is conserved across cell types, stable upon transcriptional inhibition, and distinct from the interior accumulation of global poly(A)^+^ RNA, indicating that IR-RNAs have distinct retention mechanisms and a different role of nuclear speckles in their regulation.

### LL-IR-RNAs and their transcription sites are proximal to nuclear speckles

To relate nuclear speckle enrichment measured by ARTR-seq^74^ to physical proximity, we correlated ARTR-seq log_2_FC of IR-RNAs with their median distances to nuclear speckles measured by smRNA FISH. SON enrichment was strongly inversely correlated with measured distances. Exon-derived SON log_2_FC correlated with smRNA FISH proximity measurements (Pearson’s *r* = −0.75, *P*_adj_ = 2.15 × 10^−4^; Extended Data Fig. 7b). Intron-derived and total RNA (exon and intron) SON log_2_FC showed stronger correlations (total RNA, Pearson’s *r* = −0.89, *P*_adj_ = 9.97 × 10^−7^; intron, Pearson’s *r* = −0.86, *P*_adj_ = 3.98 × 10^−6^), consistent with these measurements more directly reflecting IR-RNA positions. These results show that log_2_FC SON enrichment can provide a transcriptome-wide readout of RNA positions relative to nuclear speckles. Accordingly, ARTR-seq showed that stable RIs are enriched in SON-proximal fractions and are among the IR classes with higher SON enrichment (Fig. 3g,h). Thus, LL-IR-RNAs are associated with a nuclear speckle-proximal environment. Importantly, this enrichment does not imply accumulation within the nuclear speckle core; IR-RNAs measured by smRNA FISH localized preferentially to the periphery rather than within the core.

**Extended Data Fig. 8:**
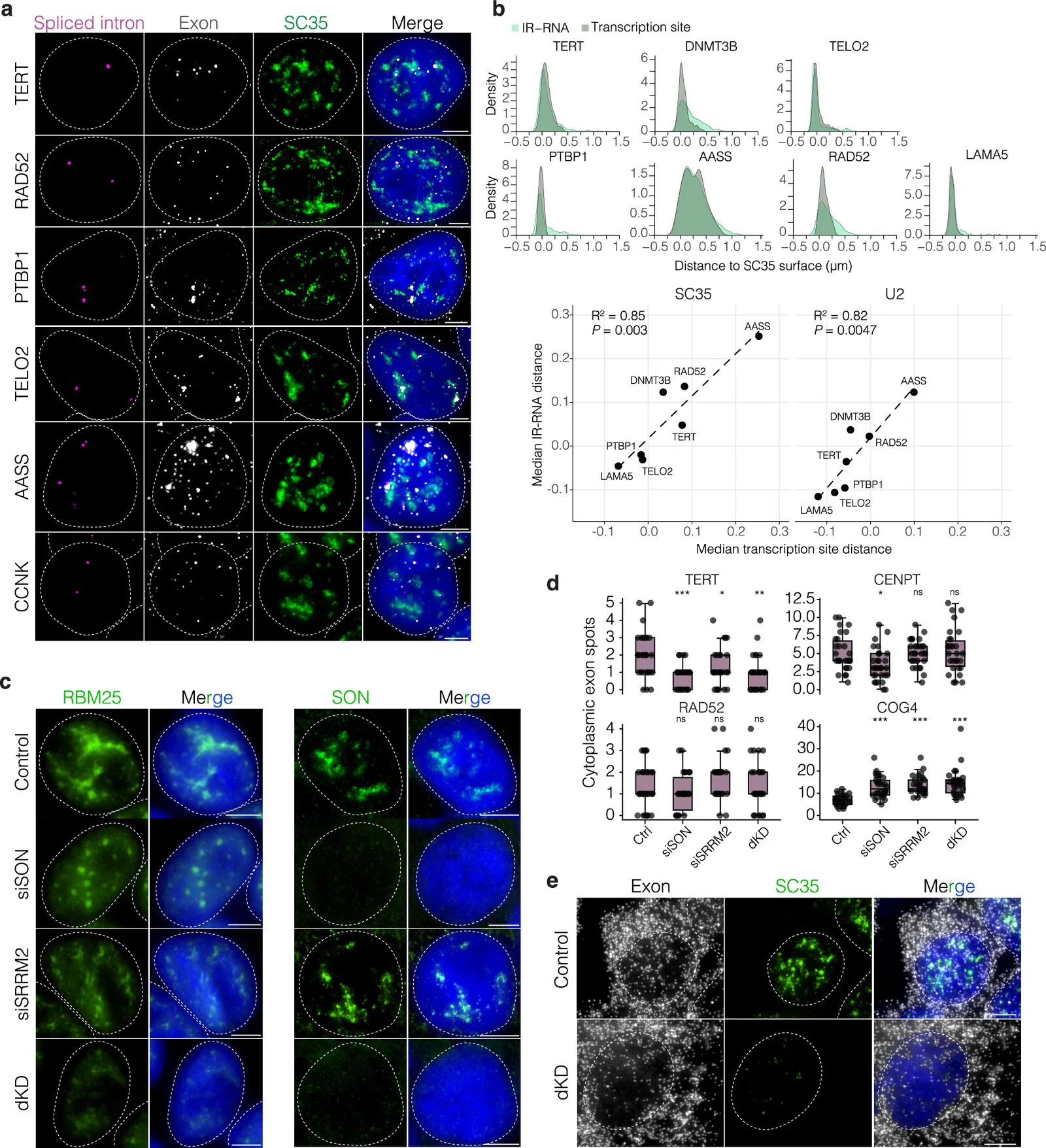
Transcription site positioning and perturbation of nuclear speckle integrity. **a**, Maximum intensity projections of exon and intron smRNA FISH combined with SC35 IF. Spliced intron, magenta; exon, white; SC35, green; nucleus in blue, outlined with a dashed line; scale bar, 2 µm. **b**, Top, density plots of the distance (µm) of IR-RNAs (green) and their transcription sites (TSs) (white) to the SC35 surface. *n* IR-RNA molecules/TS foci, respectively: AASS, 2,830/145; DNMT3B, 725/108; LAMA5, 397/184; PTBP1, 687/93; RAD52, 490/44; TELO2, 718/311; TERT, 184/73. Bottom, correlation between median transcription-site distance and median IR-RNA distance to the SC35 core surface or U2 shell surface. For SC35, Pearson’s *r* = 0.92, *P* = 3.0 × 10^−3^; for U2, Pearson’s *r* = 0.91, *P* = 4.7 × 10^−3^. *n* = 7 genes, three independent stainings. **c**, RBM25 and SON IF (green) after knockdown of SON, SRRM2 or both (double knockdown, dKD). Nucleus, blue; outlined with a dashed line; scale bar, 2 µm. **d**, Quantification of cytoplasmic exon spots after knockdown of SON, SRRM2, or both (double knockdown, dKD). Each dot represents one cell. Midline, median; box limits, first and third quartiles; whiskers, most extreme values within 1.5 × IQR of the hinges; points, individual observations. *n* = 30 cells, three independent stainings. ns = not significant, *P < 0.05, **P ≤ 0.01, ***P ≤ 0.001, as evaluated by two-sided Wilcoxon rank-sum test versus control siRNA (Ctrl). Cytoplasmic exon spots: TERT, *P*_adj_ = 3.38 × 10^−4^, 2.23 × 10^−2^, 2.39 × 10^−3^ for siSON, siSRRM2, dKD, respectively; CENPT, *P*_adj_ = 2.63 × 10^−2^ for siSON; COG4, *P*_adj_ = 7.76 × 10^−7^, 1.32 × 10^−8^, 2.64 × 10^−8^ for siSON, siSRRM2, dKD, respectively. **e**, Maximum intensity projections of GAPDH exon smRNA FISH combined with SC35 IF following siRNA-mediated knockdown of SON, SRRM2, or both (double knockdown, dKD). SC35, green; exon, white; nucleus, blue, outlined with a dashed line; scale bar, 2 µm. Source numerical data are available in source data. Source Data

To determine whether gene loci with stable or unstable IR differ in their proximity to nuclear speckles, we analysed SON tyramide signal amplification sequencing (TSA-seq)^109^, which estimates distances from gene loci to nuclear speckles and the resulting spatial patterns have been shown to be conserved across cell types^110^. We assigned genes stratified by IR and IR stability to ten TSA-seq proximity deciles, with groups 9–10 being the most nuclear speckle-proximal, which revealed that IR-containing genes were more proximal than genes without IR (~69% versus ~43% of stable IR and no IR genes, respectively, groups 9–10; *P*_adj _< 2.2 × 10^−16^, Fisher’s exact test), and that genes with stable IR were more proximal than those with unstable IR (~69% versus ~60–62% overlap with groups 9–10 of stable and unstable IR; all *P*_adj_ < 7.7 × 10^−3^, Fisher’s exact test stable versus unstable; Fig. 4i). As a validation, we performed smRNA FISH targeting exons and efficiently spliced introns of six representative genes to mark transcription sites, which were identified as one to two punctate nuclear foci with overlapping exon and spliced intron signals (Methods; Extended Data Fig. 8a). Measured distances of transcription sites to nuclear speckles correlated strongly with TSA-seq-based proximity estimates (Pearson’s *r* = −0.94, *P* = 6.2 × 10^−3^), supporting the robustness of the analysis (Fig. 4j).

We then compared the positions of transcription sites with those of the corresponding IR-RNAs. Median transcription-site distances correlated strongly with median IR-RNA distances to the surfaces of the SC35-positive nuclear speckle core (Pearson’s *r* = 0.92, *P* = 3.0 × 10^−3^) and the U2 shell (Pearson’s *r* = 0.91, *P* = 4.7 × 10^−3^; Extended Data Fig. 8b). Thus, genes more proximal to nuclear speckles tended to produce IR-RNAs that also remained relatively nuclear speckle-proximal after release from transcription sites. IR-RNAs generally showed broader positioning than transcription sites, consistent with redistribution after release, but we found no universal pattern. DNMT3B, PTBP1 and RAD52 IR-RNAs were slightly more distal to nuclear speckles than their transcription sites, whereas for TERT and AASS the two distributions were comparable. Overall, these data indicate that between-gene differences in nuclear speckle proximity are largely preserved after RNA release, supporting gene-level spatial determinants as the dominant source of positional variation. Consistent with this, gene proximity to nuclear speckles is associated with features such as GC content and intron length^111^.

Together, IR-RNAs occupy a niche at the nuclear speckle-nucleoplasm interface, linking IR stability and gene-level nuclear speckle proximity.

### IR and nuclear retention of IR-RNAs are maintained independently of nuclear speckle integrity

**Fig. 5:**
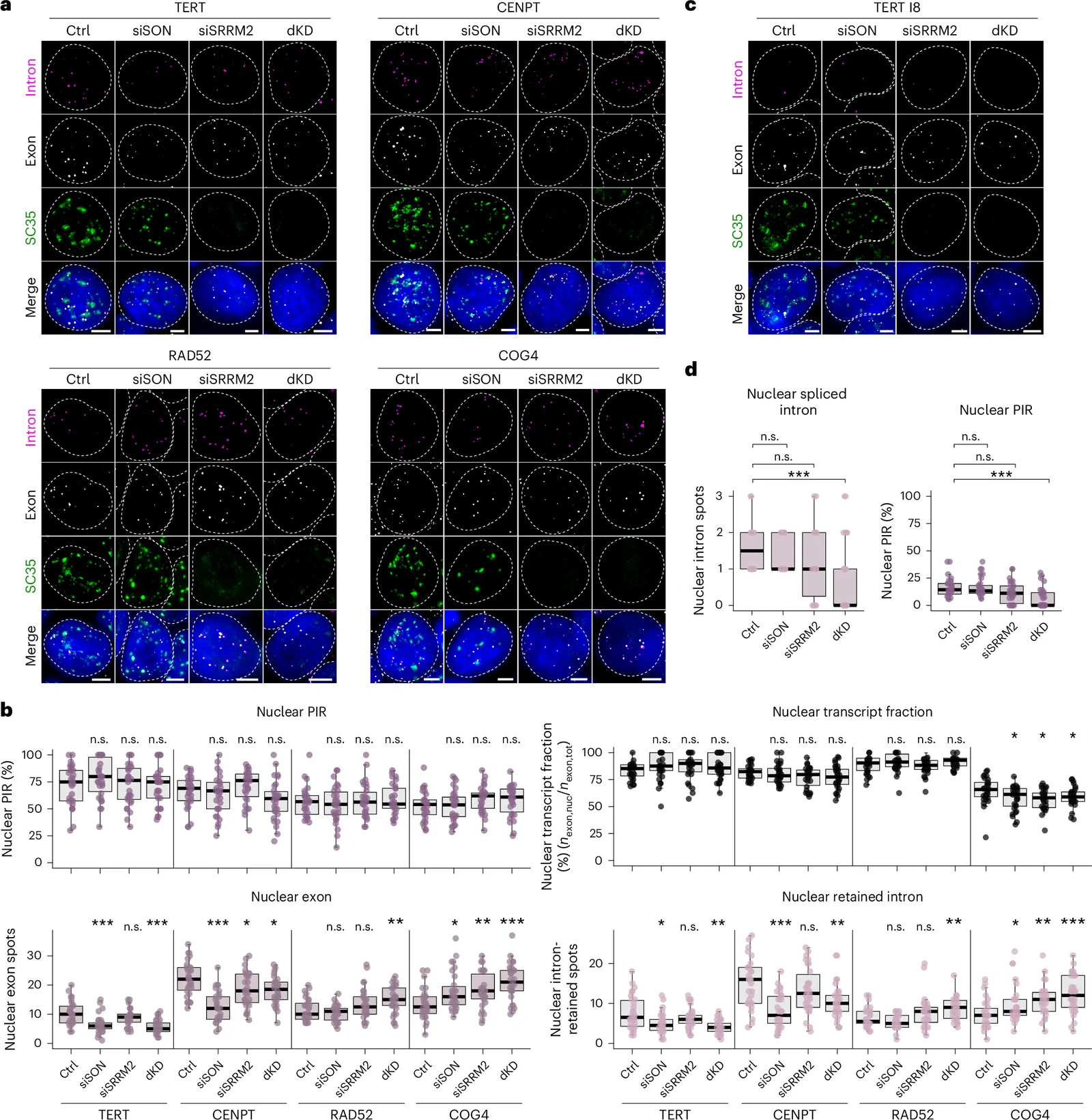
Intact nuclear speckle structure is not required for maintaining IR and nuclear retention of IR-RNAs. **a**, Maximum intensity projections of exon (white) and intron (magenta) smRNA FISH and SC35 IF (green) following knockdown of SON, SRRM2 or both (double knockdown, dKD). Nucleus, blue; scale bar, 2 µm. **b**, Quantification of nuclear PIR, nuclear exon spots, nuclear transcript fraction (%) (*n*_exon,nuc_/*n*_exon,total_), and nuclear intron-retained RNA spots for the indicated gene and condition. Each dot represents one cell. Midline, median; box limits, first and third quartiles; whiskers, most extreme values within 1.5× IQR of the hinges; points, individual observations. *n* = 30 cells, three independent stainings. n.s., not significant, *P < 0.05, **P ≤ 0.01, ***P ≤ 0.001, as evaluated by two-sided Wilcoxon rank-sum test versus control siRNA (Ctrl). Nuclear transcript fraction, COG4, *P*_adj_ = 4.63 × 10^−2^, 1.46 × 10^−2^ and 4.63 × 10^−2^, for siSON, siSRRM2 and dKD, respectively. Nuclear exon spots, TERT, *P*_adj_ = 4.06 × 10^−4^ and 4.65 × 10^−5^, for siSON and dKD, respectively; CENPT, *P*_adj_ = 1.70 × 10^−6^, 2.82 × 10^−2^ and 1.49 × 10^−2^, for siSON, siSRRM2 and dKD, respectively; RAD52, *P*_adj _= 9.63 × 10^−3^, for dKD; COG4, *P*_adj_ = 1.11 × 10^−2^, 4.54 × 10^−3^ and 1.15 × 10^−4^, for siSON, siSRRM2 and dKD, respectively. Nuclear retained-intron spots: TERT, *P*_adj _= 2.38 × 10^−2^ and 1.22 × 10^−3^, for siSON and dKD, respectively; CENPT, *P*_adj_ = 1.67 × 10^−4^ and 4.41 × 10^−3^, for siSON and dKD, respectively; RAD52, *P*_adj _= 7.32 × 10^−3^, dKD; COG4, *P*_adj_ = 3.88 × 10^−2^, 1.34 × 10^−3^ and 3.18 × 10^−4^, for siSON, siSRRM2 and dKD, respectively. **c**, Maximum intensity projections of TERT exon (white) and efficiently spliced intron 8 (magenta) smRNA FISH and SC35 IF (green) following knockdown of SON and/or SRRM2. Nucleus, blue; scale bar, 2 µm. **d**, Quantification of nuclear PIR and nuclear intron spots of TERT efficiently spliced intron 8, marking the active transcription site. Each dot represents one cell. Midline, median; box limits, first and third quartiles; whiskers, most extreme values within 1.5× IQR of the hinges; points, individual observations. *n* = 30 cells, two independent stainings. n.s., not significant, *P < 0.05, **P ≤ 0.01, ***P ≤ 0.001, as evaluated by two-sided Wilcoxon rank-sum test versus control siRNA (Ctrl). Nuclear spliced intron, *P*_adj_ = 7.95 × 10^−4^ for dKD; nuclear PIR, *P*_adj_ = 4.34 × 10^−4^ for dKD. Source data

We next asked whether nuclear speckle structure is required for maintaining IR or nuclear retention of IR-RNAs. To this end, we depleted the core nuclear speckle scaffold proteins SON and SRRM2, known to disrupt nuclear speckle structure^112^. We knocked down SON and SRRM2 individually and in combination, validated the disruption of nuclear speckles by SC35, RBM25 and SON IF (Fig. 5a; Extended Data Fig. 8c) and performed smRNA FISH on four IR-RNAs. Expression levels of individual genes responded variably; TERT nuclear and cytoplasmic RNA decreased, CENPT nuclear RNA decreased with minimal cytoplasmic changes, RAD52 was largely unchanged, and COG4 increased in both compartments across all knockdowns (Fig. 5a,b and Extended Data Fig. 8d).

Critically, despite changes in expression, PIR of examined IR-RNAs remained high and their nuclear transcript fraction was unchanged or changed only minimally across conditions (Fig. 5a,b). To rule out global export defects, we examined cytoplasmic GAPDH, whose distribution was unaffected (Extended Data Fig. 8e). To determine if these effects could be attributed to general splicing defects, we examined the splicing efficiency of *TERT* intron 8, an efficiently spliced intron. Although SRRM2 and SON/SRRM2 knockdown reduced the number of active *TERT* transcription sites, consistent with the observed reduction of TERT RNA molecules, splicing efficiency remained unchanged, indicating that disruption of SON, SRRM2 or nuclear speckle structure does not cause accumulation of unspliced transcripts (Fig. 5c,d).

Collectively, SON, SRRM2 and nuclear speckle integrity are not required to maintain IR, efficient splicing of constitutive introns, or nuclear retention of IR-RNAs examined here; instead, they contribute to the transcriptional output of examined nuclear speckle-associated genes.

### Mitosis drives coordinated LL-IR-RNA resolution

**Extended Data Fig. 9:**
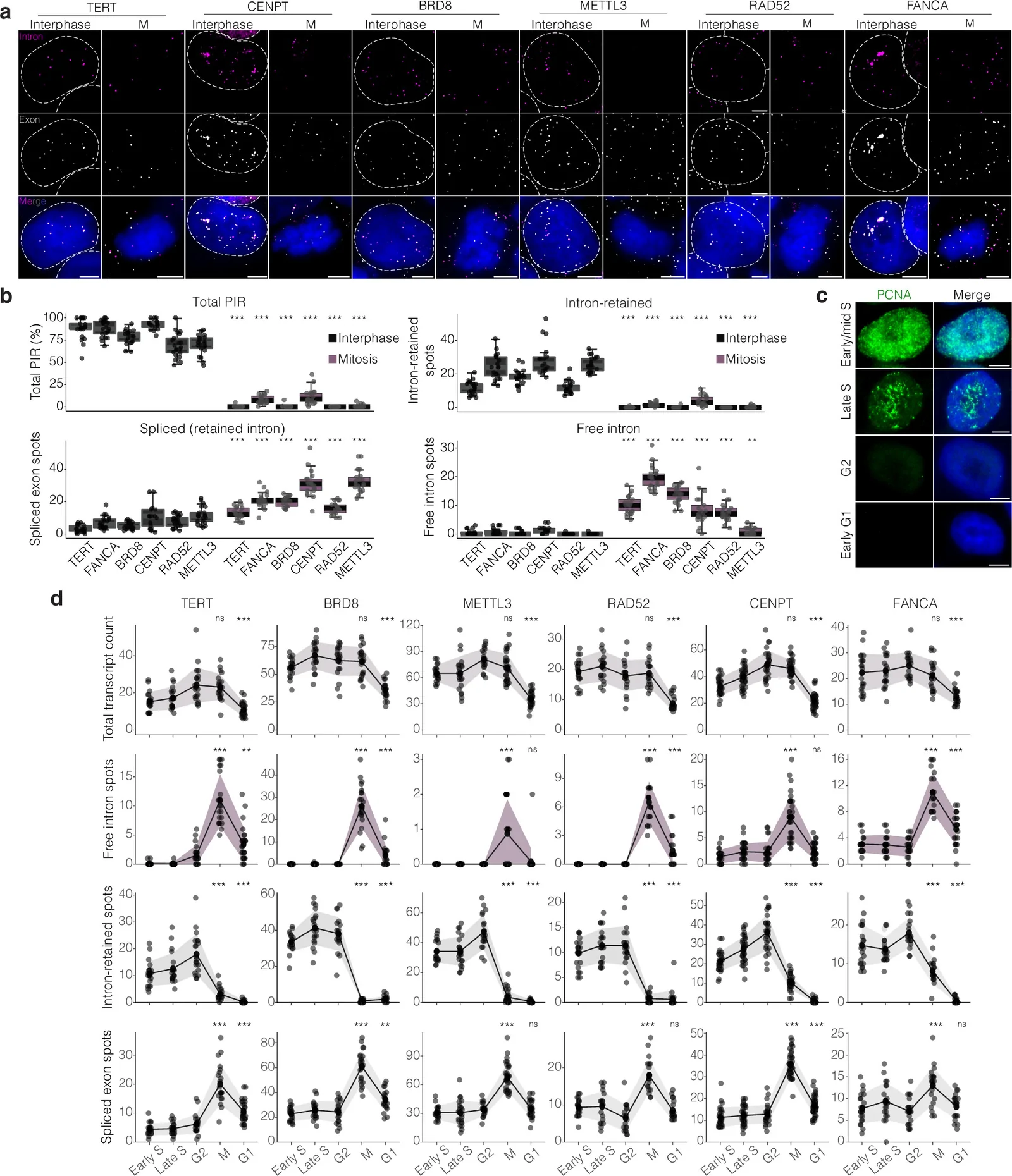
Splicing of LL-IR-RNAs in mitosis. **a**, Representative maximum intensity projections of exon/intron smRNA FISH for the indicated RNAs in hiPSCs in interphase and mitosis (M). Exon, white; intron, magenta; nucleus, blue, outlined with a dashed line; scale bar, 2 µm. **b**, Quantification of total PIR, intron-retained RNA spots, spliced RNA spots (Δ retained intron) and free intron spots in interphase and mitosis for indicated RNAs. Each dot represents one cell (*n* = 20 cells per gene and cell-cycle stage; *n* = 16 interphase cells at G2 phase for CENPT; three independent experiments). Midline, median; box limits, first and third quartiles; whiskers, most extreme values within 1.5 × IQR of the hinges; points, individual observations. **P ≤ 0.01, ***P ≤ 0.001, as evaluated by two-sided Wilcoxon rank-sum test, mitosis versus interphase. Total PIR, *P*_adj_ = 1.25 × 10^−8^, 3.72 × 10^−8^, 1.25 × 10^−8^, 1.72 × 10^−7^, 1.25 × 10^−8^, 1.87 × 10^−8^ for TERT, FANCA, BRD8, CENPT, RAD52, METTL3, respectively; intron-retained RNA spots, *P*_adj_ = 1.23 × 10^−8^, 2.95 × 10^−8^, 1.23 × 10^−8^, 1.61 × 10^−7^, 1.23 × 10^−8^, 1.83 × 10^−8^, respectively; spliced exon spots, *P*_adj_ = 7.69 × 10^−8^, 9.94 × 10^−8^, 7.69 × 10^−8^, 9.22 × 10^−7^, 7.26 × 10^−7^, 7.69 × 10^−8^, respectively; free intron spots, *P*_adj_ = 3.38 × 10^−8^, 3.38 × 10^−8^, 3.38 × 10^−8^, 1.28 × 10^−5^, 3.38 × 10^−8^, 3.44 × 10^−3^, respectively. **c**, Representative PCNA IF patterns used to assign synchronized hiPSCs to early/mid S, late S, G2, and early G1 phase. PCNA, green; nucleus, blue; scale bar, 2 µm. **d**, smRNA FISH quantification across synchronized cell-cycle stages; total transcript count, free intron spots, intron-retained RNA spots and total spliced RNA spots for the indicated RNAs. Each dot represents one cell (*n* given in Fig. 6b, three independent experiments). Black line, mean value; shadow, standard deviation; ns = not significant, *P < 0.05, **P ≤ 0.01, ***P ≤ 0.001, as evaluated by two-sided Wilcoxon rank-sum test versus G2. For gene order TERT, BRD8, METTL3, RAD52, CENPT and FANCA, total transcript count, G1: *P*_adj_ = 1.40 × 10^−7^, 2.95 × 10^−6^, 1.98 × 10^−8^, 1.80 × 10^−6^, 7.20 × 10^−12^, 1.51 × 10^−7^, respectively; free intron spots, M: *P*_adj_ = 4.83 × 10^−8^, 1.14 × 10^−8^, 1.23 × 10^−4^, 1.51 × 10^−8^, 2.75 × 10^−9^, 4.83 × 10^−8^, respectively; intron-retained RNA spots, M: *P*_adj_ = 6.12 × 10^−8^, 3.31 × 10^−8^, 4.85 × 10^−9^, 6.12 × 10^−8^, 3.65 × 10^−11^, 2.60 × 10^−7^, respectively; G1: *P*_adj_ = 6.95 × 10^−10^, 4.11 × 10^−8^, 7.98 × 10^−9^, 3.01 × 10^−8^, 1.03 × 10^−12^, 1.83 × 10^−8^, respectively; spliced exon spots, M: *P*_adj_ = 2.02 × 10^−7^, 5.23 × 10^−8^, 6.16 × 10^−9^, 2.38 × 10^−7^, 4.08 × 10^−11^, 7.73 × 10^−5^, respectively; free intron spots, G1: *P*_adj_ = 9.95 × 10^−3^, 1.27 × 10^−4^, 3.22 × 10^−5^, 5.85 × 10^−4^ for TERT, BRD8, RAD52, FANCA, respectively; spliced exon spots, G1: *P*_adj_ = 4.22 × 10^−4^, 4.13 × 10^−3^, 5.82 × 10^−4^ for TERT, BRD8, CENPT, respectively. Source numerical data are available in source data. Source Data

Building on our previous findings that TERT RI is spliced in mitosis^23^, we asked whether mitosis broadly functions as a temporal window for resolving nuclear IR-RNAs from genes carrying LL-RIs. We treated hiPS cells with nocodazole and performed smRNA FISH on six genes harbouring LL-RIs (*BRD8*, *METTL3*, *TERT*, *RAD52*, *FANCA* and *CENPT*), defined as having at least one LL-RI and selected for high basal PIR and nuclear RNA enrichment (Extended Data Fig. 9a,b). Most nocodazole-treated hiPS cells were in mitosis, and approximately 20% were in interphase, as shown previously^113^.

In mitosis, all six LL-IR-RNAs underwent extensive splicing and were released into the cytosol. IR-RNA numbers decreased sharply, while spliced RNAs (ΔRI) increased reciprocally (Extended Data Fig. 9a,b). The number of newly spliced transcripts closely matched that of IR-RNAs in interphase, suggesting that the spliced pool arises from the interphase IR-RNAs. Consequently, total PIR dropped sharply, while free intron signal, which was nearly absent in interphase, strongly increased, indicating active intron excision and transient stabilization of excised introns (Extended Data Fig. 9b).

**Fig. 6:**
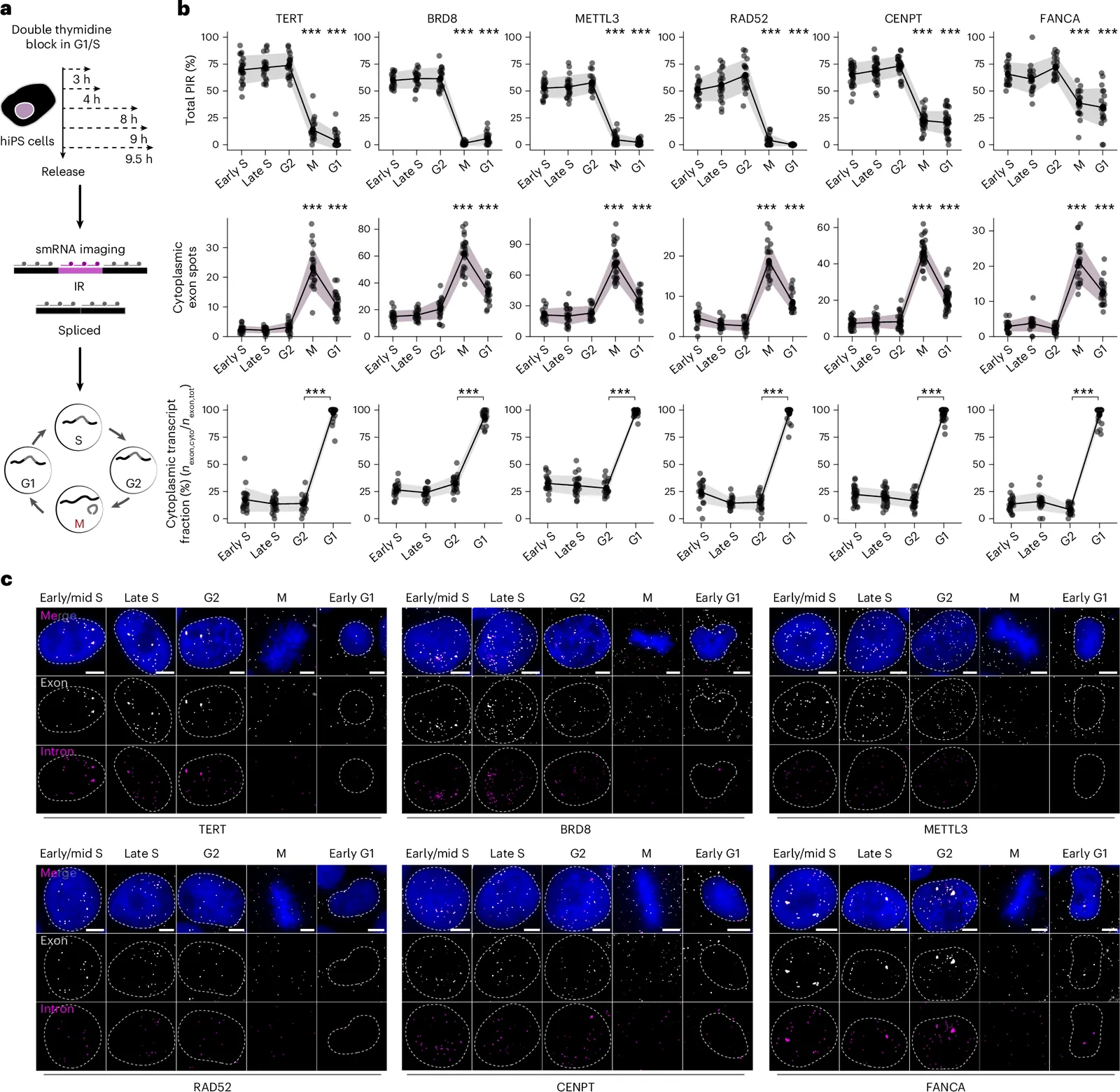
Mitosis drives splicing of stable IR-RNAs and cytoplasmic accumulation of the resulting mRNAs. **a**, Schematic of the experimental design. hiPS cells were synchronized by double thymidine block at the G1/S boundary, released and analysed by smRNA FISH at the indicated timepoints corresponding to early/mid S, late S, G2, M and early G1 phases. **b**, Quantification of total PIR (top), cytoplasmic exon spots (middle) and cytoplasmic transcript fraction (%) (*n*_exon,cyto_/*n*_exon,total_) (bottom) for the indicated RNAs across synchronized cell-cycle stages. Each dot represents one cell. Black line, mean value; shadow, standard deviation; *n* values are given for early/mid S, late S, G2, mitosis and early G1, respectively: TERT 21, 20, 21, 21, 29; BRD8 20, 20, 20, 23, 21; METTL3 20, 23, 20, 32, 27; RAD52 20, 20, 20, 20, 21; CENPT 30, 38, 29, 36, 44; FANCA 22, 20, 20, 21, 22; three independent experiments. Cytoplasmic transcript fraction is not shown for mitotic cells. ***P ≤ 0.001, as evaluated by two-sided Wilcoxon rank-sum test versus G2. Total PIR, M and G1, respectively: TERT, *P*_adj_ = 4.66 × 10^−8^, 1.59 × 10^−9^; BRD8, *P*_adj_ = 3.96 × 10^−8^, 5.49 × 10^−8^; METTL3, *P*_adj_ = 5.48 × 10^−9^, 6.89 × 10^−9^; RAD52, *P*_adj _= 5.73 × 10^−8^, 6.89 × 10^−9^; CENPT, *P*_adj_ = 3.51 × 10^−11^, 3.91 × 10^−12^; FANCA, *P*_adj _= 5.73 × 10^−8^, 1.94 × 10^−7^. Cytoplasmic exon spots, M and G1, respectively: TERT, *P*_adj_ = 4.36 × 10^−8^, 1.61 × 10^−8^; BRD8, *P*_adj_ = 4.36 × 10^−8^, 3.56 × 10^−5^; METTL3, *P*_adj _= 5.48 × 10^−9^, 6.13 × 10^−5^; RAD52, *P*_adj _= 6.11 × 10^−8^, 5.59 × 10^−8^; CENPT, *P*_adj_ = 3.37 × 10^−11^, 9.18 × 10^−11^; FANCA, *P*adj = 4.87 × 10^−8^, 5.51 × 10^−8^. Cytoplasmic transcript fraction, G1 versus G2: TERT, *P*_adj_ = 7.09 × 10^−10^; BRD8, *P*_adj_ = 4.58 × 10^−8^; METTL3, *P*_adj_ = 9.21 × 10^−9^; RAD52, *P*_adj _= 1.71 × 10^−8^; CENPT, *P*_adj _= 1.14 × 10^−12^; FANCA, *P*_adj_ = 1.71 × 10^−8^. **c**, Representative maximum intensity projections of exon (white) and intron (magenta) smRNA FISH in synchronized hiPS cells at the indicated cell-cycle stages. Hoechst, blue; scale bar, 2 µm. Source data

To monitor LL-IR-RNAs across the cell cycle and immediately after mitosis, we synchronized hiPS cells using a double thymidine block and collected cells at defined intervals after release spanning early-to-late S and G2 phase, mitosis and early G1 for smRNA FISH with single-cell-level staging using PCNA immunostaining, cell size and absent or early-onset transcription in early G1, as defined by exon–intron smRNA FISH (Methods; Fig. 6a and Extended Data Fig. 9c).

All six LL-IR-RNAs remained enriched in the nucleus throughout early and late S phase and persisted in G2, with stable PIR and IR-RNA numbers, indicating sustained IR and nuclear retention before mitosis (Fig. 6b,c and Extended Data Fig. 9d). By contrast, mitosis was accompanied by a rapid splicing transition. Total PIR dropped sharply across all candidates (Fig. 6b,c), paralleled by a marked reduction in IR-RNA count (Extended Data Fig. 9d). This shift was accompanied by a reciprocal increase in fully spliced transcripts (ΔRI) and an accumulation of free intron signal. Importantly, total RNA abundance per cell did not change significantly between G2 and mitosis, supporting mitosis-triggered processing rather than degradation (Extended Data Fig. 9d).

Upon mitotic exit, early G1 cells were dominated by cytoplasmic spliced transcripts and showed a marked increase in cytoplasmic RNA fraction, despite a lack of detectable transcription or its early-onset (Fig. 6b,c). The cytoplasmic RNA pool in early G1 corresponded to approximately half of the RNA in mitosis, consistent with partitioning into daughter cells, and exceeded cytoplasmic RNA levels measured in G2 (Fig. 6b). A fraction of free intron signal persisted in early G1 cytoplasm (Extended Data Fig. 9d), consistent with reports of cytoplasmic introns in vertebrates^114^.

Together, these data demonstrate that LL-IR-RNAs undergo coordinated splicing during mitosis, converting nuclear IR-RNAs into spliced mRNAs without total RNA loss. The resulting spliced transcripts are inherited by daughter cells in early G1 as dominant RNA species prior to transcriptional reactivation. We propose that this mechanism provides early G1 daughter cells with mature transcripts encoding critical regulators, without requiring new transcription, splicing and export, which collectively can take tens of minutes to more than an hour^115–118^. These findings place LL-IR-RNAs operationally within the intron-detention class as a long-lived subtype and identify mitosis as the physiological cue for their splicing.

### Kinase-dependent regulation of mitotic IR resolution

Entry into mitosis is accompanied by a broad phospho-remodelling programme driven by a kinase-dominant state established by cyclin-dependent kinase 1 (CDK1) activation and reduced protein phosphatase 1 and 2 (PP1/PP2) activity^119–121^. CLKs, which localize to nuclear speckles and peak at G2/M phase, phosphorylate SR proteins and other RS-domain-containing splicing factors, weakening their multivalent interactions, promoting release into the nucleoplasm and nuclear speckle dissolution in mitosis^44–46,122^, while DYRK3 drives a broader phosphorylation programme promoting dissolution of multiple condensates, including nuclear speckles^47^.

**Fig. 7:**
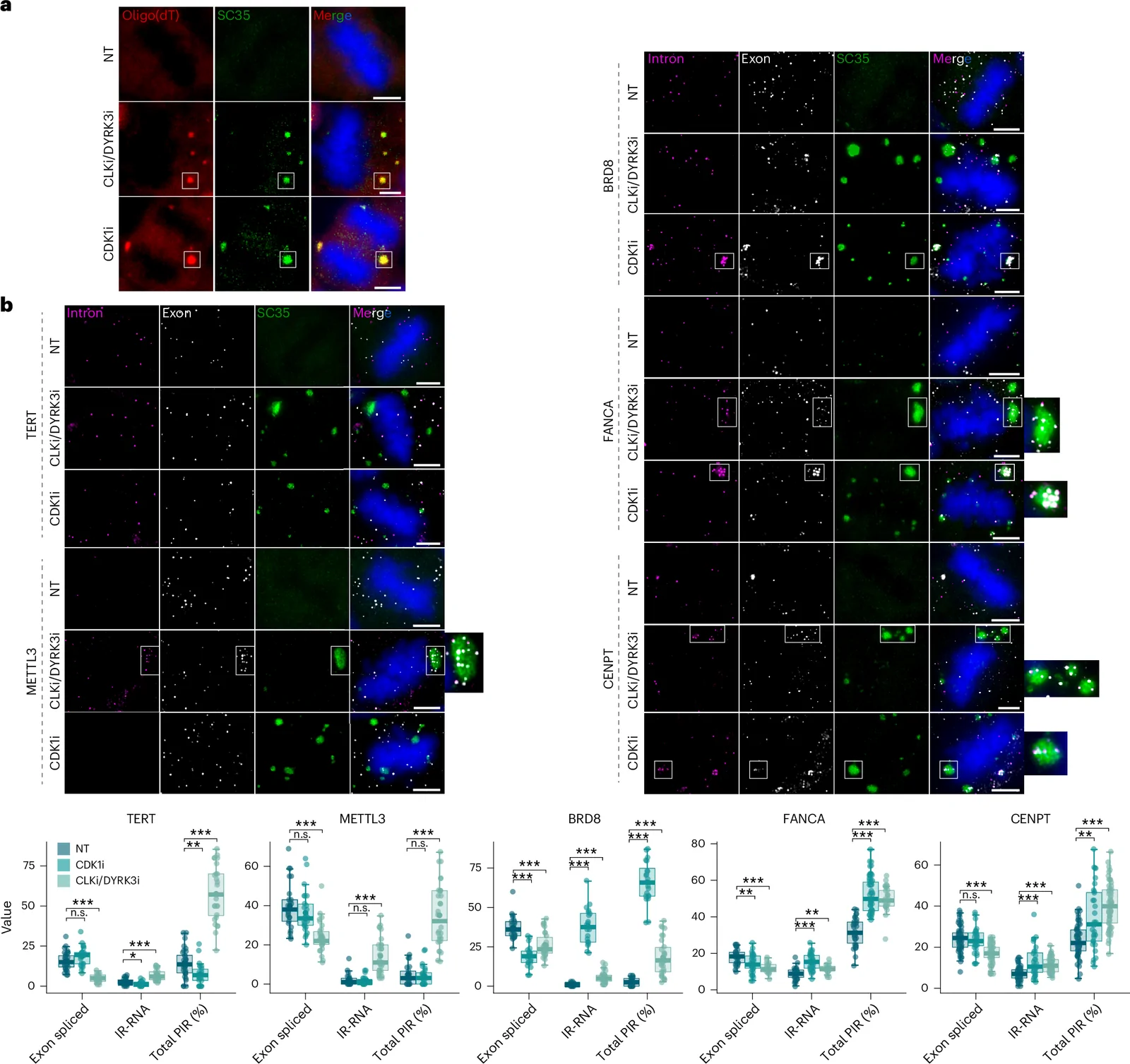
Splicing of RIs in mitosis is kinase dependent. **a**, Maximum intensity projections of oligo(dT) smRNA FISH (red) and SC35 IF (green). Hoechst, blue; scale bar, 2 µm. **b**, Top: maximum intensity projections of exon (white) and intron (magenta) smRNA FISH and SC35 IF (green) in non-treated (NT) hiPS cells or after CLKi/DYRK3i or CDK1i. Hoechst, blue; scale bar, 2 µm. Bottom: smRNA FISH quantification of spliced exon spots (ΔRI), retained-intron RNA spots and total PIR in mitosis. Midline, median; box limits, first and third quartiles; whiskers, most extreme values within 1.5× IQR of the hinges; points, individual cells. n.s., not significant, *P < 0.05, **P ≤ 0.01, ***P ≤ 0.001 as evaluated by two-sided Wilcoxon rank-sum test versus non-treated cells (NT). Spliced exon spots: CDK1i, *P*_adj_ = 2.86 × 10^−7^ and 1.07 × 10^−3^, for BRD8 and FANCA, respectively; CLKi/DYRK3i, *P*_adj_ = 3.41 × 10^−9^, 1.34 × 10^−6^, 4.16 × 10^−5^, 1.91 × 10^−6^ and 1.23 × 10^−8^, for TERT, METTL3, BRD8, FANCA and CENPT, respectively. Retained-intron RNA spots: CDK1i, *P*_adj_ = 1.05 × 10^−2^, 6.53 × 10^−8^, 4.84 × 10^−7^ and 6.31 × 10^−4^, for TERT, BRD8, FANCA and CENPT, respectively; CLKi/DYRK3i, *P*_adj _= 1.84 × 10^−6^, 7.69 × 10^−8^, 2.86 × 10^−7^, 2.13 × 10^−3^ and 3.59 × 10^−7^, for TERT, METTL3, BRD8, FANCA and CENPT, respectively. Total PIR: CDK1i, *P*_adj _= 3.61 × 10^−3^, 7.01 × 10^−8^, 1.07 × 10^−8^ and 1.94 × 10^−3^ for TERT, BRD8, FANCA and CENPT, respectively; CLKi/DYRK3i, *P*_adj _= 2.88 × 10^−9^, 1.27 × 10^−8^, 7.69 × 10^−8^, 1.55 × 10^−6^ and 6.27 × 10^−11^ for TERT, METTL3, BRD8, FANCA and CENPT, respectively. *n*, mitotic cells from four independent experiments, for NT, CDK1i and CLKi–DYRK3i, respectively: CENPT 55, 32, 55; METTL3 25, 26, 30; TERT 32, 25, 27; BRD8 25, 20, 30; FANCA 34, 38, 20. Source data

**Extended Data Fig. 10:**
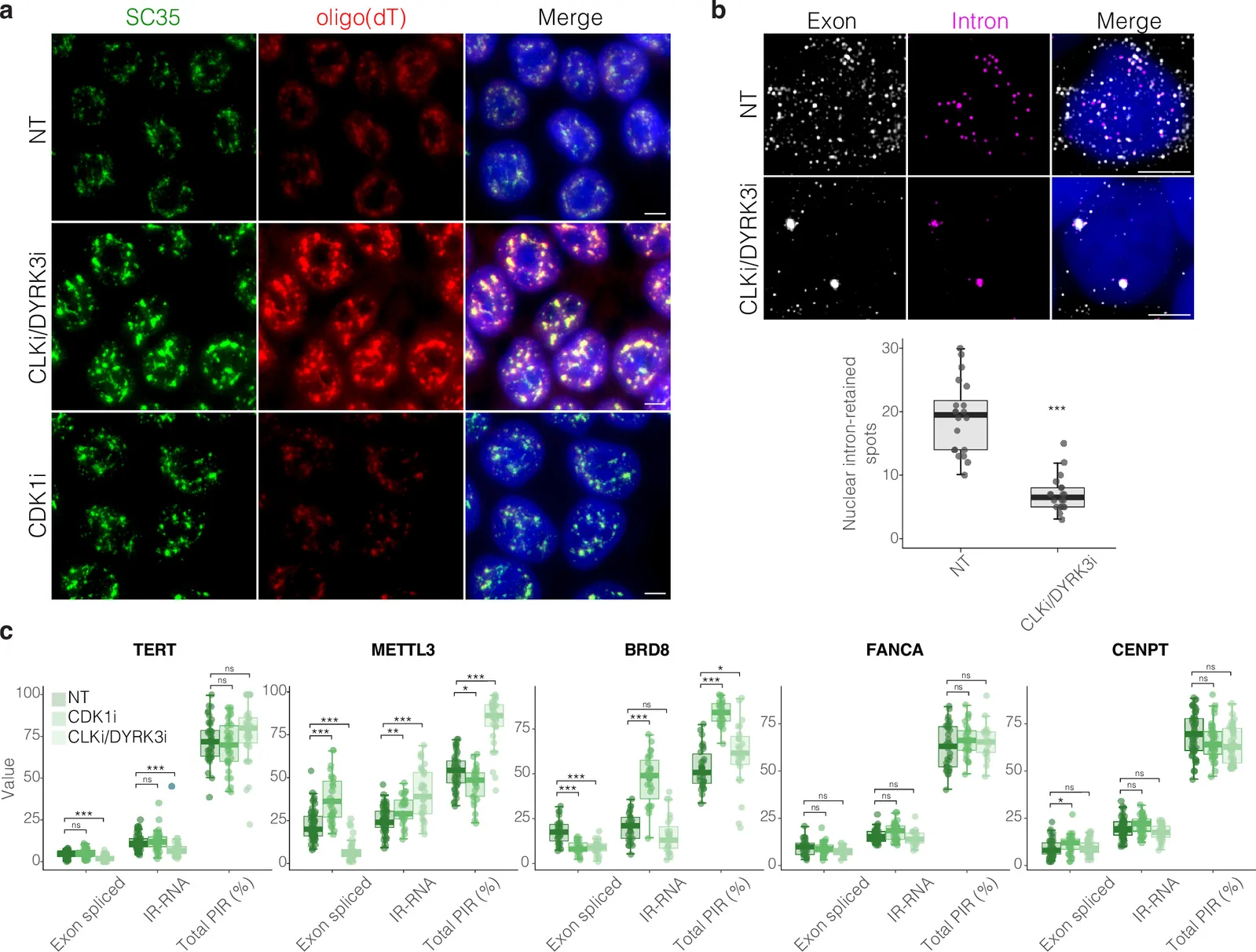
Splicing of retained introns in mitosis is kinase-dependent. **a**, Maximum intensity projections of oligo(dT) smRNA FISH (red), and SC35 IF (green) in non-treated cells (NT) or cells treated with CLKi/DYRK3i or CDK1i. Nucleus, blue; scale bar, 2 µm. **b**, Maximum intensity projections of SRSF7 exon (white) and intron (magenta) smRNA FISH in non-treated cells (NT) or cells treated with CLKi/DYRK3i. Nucleus, blue; scale bar, 2 µm. Below, quantification of intron-retained RNA spots of the indicated treatments. Each dot represents one cell. Midline, median; box limits, first and third quartiles; whiskers, most extreme values within 1.5 × IQR of the hinges; points, individual observations. ***P ≤ 0.001, as evaluated by two-sided Welch’s t-test versus non-treated cells (NT), *n* = 20 cells, two independent stainings. *P* = 4.47 × 10^−9^. **c**, Bottom, smRNA FISH quantification of spliced exon spots (Δ retained intron), intron-retained RNA spots and total PIR in interphase. Midline, median; box limits, first and third quartiles; whiskers, most extreme values within 1.5 × IQR of the hinges; points, individual observations. ns = not significant, *P < 0.05, **P ≤ 0.01, ***P ≤ 0.001 evaluated by two-sided Wilcoxon rank-sum test versus non-treated cells (NT). *n* values are given for NT, CDK1i and CLKi/DYRK3i, respectively: TERT 42, 36, 36; BRD8 30, 27, 23; METTL3 47, 29, 33; CENPT 45, 35, 34; FANCA 35, 30, 20; four independent experiments. Spliced exon spots, CDK1i, *P*_adj_ = 6.11 × 10^−6^, 1.51 × 10^−6^, 2.45 × 10^−2^ for METTL3, BRD8, CENPT, respectively; CLKi/DYRK3i, *P*_adj_ = 3.96 × 10^−5^, 3.41 × 10^−9^, 8.78 × 10^−6^ for TERT, METTL3, BRD8, respectively. IR-RNA spots, CDK1i, *P*_adj_ = 5.41 × 10^−3^, 4.44 × 10^−8^ for METTL3, BRD8, respectively; CLKi/DYRK3i, *P*_adj_ = 1.37 × 10^−5^, 4.87 × 10^−6^ for TERT, METTL3, respectively. Total PIR, CDK1i, *P*_adj_ = 1.32 × 10^−2^, 3.41 × 10^−9^ for METTL3, BRD8, respectively; CLKi/DYRK3i, *P*_adj_ = 2.88 × 10^−9^, 3.36 × 10^−2^ for METTL3, BRD8, respectively. *n* for NT, CDK1i and CLKi/DYRK3i, respectively: TERT 42, 36, 36; BRD8 30, 27, 23; METTL3 47, 29, 33; CENPT 45, 35, 34; FANCA 35, 30, 20; four independent experiments. Source numerical data are available in source data. Source Data

To determine whether mitotic LL-IR-RNA splicing depends on kinase-driven remodelling, we inhibited CLKs and DYRK3 (CLKi/DYRK3i) to block broad phosphorylation-dependent remodelling of splicing and condensate environment in mitosis, and CDK1 (CDK1i) to block upstream mitotic phospho-remodelling. As previously reported, CLKi/DYRK3i promoted poly(A)^+^ RNA accumulation in nuclear speckles in interphase, and disrupted nuclear speckle disassembly in mitosis^41^ (Fig. 7a and Extended Data Fig. 10a). CDK1i also prevented nuclear speckle dissolution in mitosis, indicating that CDK1 contributes to this process. Undissolved mitotic nuclear speckles maintained poly(A)^+^ RNA retention capacity (Fig. 7a).

CLKi/DYRK3i increased mitotic PIR across all candidate LL-IR-RNAs, with the strongest effects observed for TERT and METTL3, followed by FANCA, CENPT and BRD8 (+43.1%, +30.1%, +17.3%, +17% and +15.6%, respectively; all *P*_adj_ < 1.6 × 10⁻^6^, two-sided Wilcoxon rank-sum test), reflecting reduced spliced RNA and increased IR-RNA isoforms (Fig. 7b), indicating splicing inhibition instead of decay.

CDK1i affected mitotic PIR in a transcript-specific manner, with the strongest increase in BRD8, followed by FANCA and CENPT (+63.3%, +20.3% and +10.8%, respectively; all *P*_adj_ < 2.0 × 10^−3^, two-sided Wilcoxon rank-sum test; Fig. 7b). METTL3 was not significantly changed, whereas TERT showed a slight reduction (−7%; *P*_adj_ = 3.6 × 10^−3^, two-sided Wilcoxon rank-sum test; Fig. 7b). Consistent with these changes, IR-RNA increased for BRD8, FANCA and CENPT, decreased for TERT, whereas spliced RNA was reduced for BRD8 and FANCA. Unspliced IR-RNAs tended to co-localize with undissolved mitotic nuclear speckles, except for TERT, whereas exon-only or excised intron signals did not, indicating that exon–intron architecture promotes association with nuclear speckles. Thus, while both CDK1i and CLKi/DYRK3i prevented nuclear speckle dissolution, their impact on RI splicing is transcript specific.

To compare this behaviour with a canonical CLK-responsive detained intron, we examined SRSF7, a previously described CLK-responsive target in interphase^17^ that falls within the short-lived class in our dataset. As expected, CLKi strongly reduced SRSF7 detained-intron RNA, accompanied by brighter transcription site foci (Extended Data Fig. 10b). In interphase, CLKi/DYRK3i strongly increased METTL3 PIR and only modestly increased BRD8 PIR (+29.4% and +7.8%; *P*_adj_ = 2.9 × 10^−9^ and *P*_adj_ = 3.4 × 10^−2^, respectively, two-sided Wilcoxon rank-sum test; Extended Data Fig. 10c), with the remaining candidates unaffected, suggesting that RS-domain phosphorylation tunes METTL3 IR across the cell cycle. CDK1i increased BRD8 PIR and modestly decreased METTL3 PIR in interphase (+30.8% and −7.3%; *P*_adj_ = 3.4 × 10^−9^ and *P*_adj _= 1.3 × 10^−2^, respectively, two-sided Wilcoxon rank-sum test; Extended Data Fig. 10c), with other candidates unchanged. Thus, unlike the broad mitotic response, LL-IR-RNAs showed only selective sensitivity to kinase perturbation in interphase, supporting mitosis as the major window for coordinated kinase-dependent IR resolution.

Together, these data indicate that kinase activity is required for full LL-IR-RNA resolution in mitosis: CLK/DYRK3 inhibition broadly impairs splicing across candidates, while CDK1 contributes in a transcript-specific manner.

## Discussion

RIs in polyadenylated nuclear transcripts can be resolved through post-transcriptional splicing as a regulated layer of gene expression control^8,17,18,21^. Here, integrating transcription inhibition with deep-learning and high-resolution imaging, we identify a long-lived population of RIs in hiPS cells that persists for hours. Annotated detained introns distribute across multiple kinetic groups, indicating that LL-IR-RNAs represent a distinct subset within the broader detained-intron framework.

We identify a spatially defined niche for IR-RNAs at the nuclear speckle periphery, largely excluded from the SC35-positive core and concentrated at the U2 shell-nucleoplasm interface, resolving ambiguities left by proximity-labelling studies and distinguishing IR-RNAs from bulk poly(A)^+^ RNAs enriched within nuclear speckles^38,41,42^. LL-IR-RNAs and their transcription sites occupy more nuclear speckle-proximal positions than SL-IR-RNAs, linking IR stability to subnuclear architecture. IR-RNA localization described here is distinct from paraspeckles, which are typically absent in hiPS cells, lack the SON scaffold and would not be captured with SON-based proximity approaches^123–125^.

This spatial organization is mechanistically informative. Proximity to nuclear speckles has been associated with efficient splicing^64^, and transcription sites localize adjacent to nuclear speckles^61–64,126^. Recently, nuclear speckles were linked to RNA processing of GC-rich isochores^111^. Yet, LL-IR-RNAs remain unspliced even while positioned at the nuclear speckle periphery, indicating the presence of *cis*-encoded inhibitory features that delay splicing progression despite a splicing-permissive environment. Consistent with this, RIs and LL-RIs are enriched for weak 5′ splice sites, elevated GC content, stronger predicted secondary structures, and sequence motifs capable of forming non-canonical conformations. In particular, G-quadruplex-forming sequences are enriched near splice-regulatory elements (donor site, branch point), where, together with elevated GC content and short intron length, they may impede spliceosome progression until specific cues trigger resolution. Motif analysis identifies candidate *trans*-acting regulators, including HNRNPF and SRSF1, known to influence IR^127,128^, and EWSR1 and RBM4-family proteins^129,130^, the latter localizing to nuclear speckles, binding G-quadruplexes, and linked to splicing inhibition, making them plausible candidates for coupling GC-rich, structure-prone *cis*-features to splicing control^98,101–103^. These observations suggest that IR longevity reflects an interplay between RNA-intrinsic features, RBP interactions and the local nuclear environment.

Nuclear speckle perturbation experiments did not lead to LL-IR-RNA splicing or export, indicating that their periphery may function as a permissive interface where transcripts remain poised for resolution. IR-RNAs distribute around multiple nuclear speckles, consistent with diffusion during extended nuclear residence. Hundreds of IR-RNAs concentrated at the periphery may enable noncoding functions, such as protein or DNA binding^76,131^, or collectively define a distributed nuclear speckle network through multivalent interactions.

Finally, we identify mitotic reprogramming as a regulatory, coordinated window for synchronous resolution of LL-IR-RNAs, generating spliced transcripts that populate the cytoplasm of mitotic and early G1 daughter cells, consistent with an inheritance mechanism that reduces reliance on de novo transcription, splicing and export, which collectively can take tens of minutes to over an hour^115–118^. We emphasize that our data do not argue that these introns are spliced exclusively during mitosis, but rather that mitotic remodelling is a window in which they are resolved in a coordinated burst. This transition depends on kinase-driven remodelling of the splicing and condensate environment, coupling mitotic reorganization to coordinated intron resolution. CLK-dependent IR regulation has been described in interphase contexts, including promoting or suppressing detained intron splicing, CLK-dependent SR protein phosphorylation in nuclear stress bodies, CLK2 condensates linked to increased IR, and autoregulation of CLKs through IR-Clk1/4 transcripts^17,34,75,132^. Notably, this pathway is not uniform. METTL3 showed increased interphase PIR upon CLKi/DYRK3i, suggesting flexible tuning to meet ongoing cellular demands, whereas TERT IR showed tight coupling to mitosis, consistent with telomerase restriction to proliferative contexts, as we hypothesized earlier^23^. CLK and DYRK3 individual contributions remain to be determined. This underscores dynamic regulation of LL-IR-RNAs, with some transcripts requiring flexible availability and others being tightly gated to mitosis.

A long-lived IR programme was previously described in male germ cells, where IR-RNAs persist throughout differentiation and are spliced post-meiotically when de novo mRNA synthesis is suppressed^133^, suggesting that large-scale nuclear reorganization events during mitosis or meiosis may create regulatory windows for coordinated intron resolution to replenish cytoplasmic mRNAs when transcription is transiently repressed. These findings extend observations that alternative splicing oscillates across the cell cycle^60,122^, revealing a spatial interphase niche and a mitosis-linked intron resolution programme.

Functionally, this timing may ensure the rapid availability of mature transcripts as cells exit mitosis. Many LL-IR-RNAs encode regulators of DNA repair, genome maintenance and cell cycle. CENP-T is a core kinetochore component whose centromeric incorporation and outer kinetochore assembly are cell-cycle regulated^77,134^. FANCA maintains centrosome and spindle organization, is required for replication-associated DNA damage repair and supports cytokinesis during mitosis; its depletion induces G1/S arrest^135–138^. METTL3, the catalytic subunit of the m^6^A writer complex, regulates cell cycle progression and facilitates homologous recombination repair^81,139–141^. BRD8, a bromodomain-containing chromatin regulator, regulates the expression of prereplicative complex subunits in G1 phase, and its depletion induces G1 arrest^142^. TERT, the catalytic dose-limiting component of the telomerase complex, is needed for telomere maintenance in mitotically active cells^80,143^. Together, these examples support a model in which mitotic splicing generates translation-competent mRNAs precisely when transcription is globally repressed and pre-existing mRNA and protein pools have been halved by cell division, delivering a supply of mRNAs as daughter cells reform to meet early G1 demands that prime S phase and replication-coupled genome maintenance.

Our study identifies a nuclear speckle–peripheral reservoir of LL-IR-RNAs, defines its sequence and spatial determinants, and links mitotic remodelling to their coordinated release. Together, these findings show how intronic sequence, subnuclear organization and cell cycle transitions converge to gate mRNA availability in time and space.

## Methods

### Cell culture and cell cycle synchronization

hiPS cells WTC-11 (GM25256, male, Coriell Institute) were cultured on Vitronectin (Thermo Fisher Scientific) coated plates or 18-mm round glass coverslips for smRNA FISH and IF in StemFlex basal medium (Thermo Fisher Scientific) with StemFlex supplement (Thermo Fisher Scientific) and 2.5% penicillin–streptomycin. RevitaCell supplement with ROCK inhibitor (Thermo Fisher Scientific) was added when thawing the cells. hiPS cells were passaged with 0.5 mM EDTA in Dulbecco’s phosphate-buffered saline (DPBS). HUVECs (c-12203, pool of male/female cells, PromoCell) were freshly isolated from donors and cultured for no more than two passages to preserve their native transcriptional and nuclear architecture. They were cultured on 25 ng ml^−1^ fibronectin (Sigma-Aldrich)-coated glass coverslips (for smRNA FISH and IF) in endothelial cell basal medium (PromoCell) with endothelial cell growth medium supplement mix (PromoCell).

Actinomycin D (ActD, Sigma-Aldrich) was used at a final concentration of 5 μg ml^−1^ in complete growth media. Cell pellets and coverslips were collected at 0 h, 0.5 h, 2 h and 4.5 h after adding ActD. Kinase inhibition was performed using DYRK3 inhibitor GSK626616 (2 µM; MedChemExpress), CLK inhibitor CLK-IN-T3 (1.3 µM; MedChemExpress), CDK1 inhibitor RO-3306 (15 µM; Selleckchem) or dimethyl sulfoxide as a control, diluted in StemFlex medium for 5–7 h.

Cell cycle synchronization at the G2/M phase was performed with nocodazole (75 ng ml^−1^; Sigma-Aldrich) for 16 h (ref. ^144^). Synchronization at the G1/S phase was achieved with a double thymidine block^144^. For the first block, cells were incubated with 2 mM thymidine (Sigma-Aldrich) for 14.5 h. Thymidine-containing medium was then removed, cells were washed with DPBS and fresh growth medium was added to release cells for 9 h. A second thymidine block was subsequently applied for 16 h. To initiate the second release (defined as *t* = 0 h), thymidine-containing medium was removed, cells were washed with DPBS and fresh growth medium was added.

Coverslips were collected at defined time windows after the second release to enrich for specific cell cycle stages. S phase samples were collected at 3 h and 4 h after release. G2, M, and early G1 samples were collected at 8, 9 and 9.5 h after release. Cell cycle stage was assessed by nuclear size and PCNA staining pattern^145^, as explained in the ‘Wide-field microscopy and image analysis’ section.

### siRNA transfection

The day before transfection, hiPS cells were seeded on vitronectin-coated coverslips. Small interfering RNA (siRNA) targeting SON and/or SRRM2 (Thermo Fisher Scientific) were transfected at a final concentration of 40 nM using Lipofectamine RNAiMAX reagent (Thermo Fisher Scientific) following the manufacturer’s guidelines. After 6 h, the transfection mix was replaced by complete StemFlex growth media, and the cells were incubated for an additional 24 h. smRNA FISH and IF were performed as described below.

### RNA fractionation

RNA fractionation was performed as described previously^146,147^. In brief, hiPS cells were rinsed twice with ice-cold phosphate-buffered saline (PBS), scraped and centrifuged at 500*g* for 5 min. Cell pellets were lysed with 175 μl/10^6^ cells of ice-cold lysis buffer (50 mM Tris–HCl pH 8.0, 140 mM NaCl, 1.5 mM MgCl_2_, 0.5% NP-40, 1:200 dilution of 20 U μl^−1^ SUPERase RNase inhibitor) and incubated on ice for 10 min. The lysate was centrifuged at 300*g* for 3 min at 4 °C to pellet the nuclei, and the supernatant was collected as the cytoplasmic fraction. Finally, the nuclear and the cytoplasmic fractions were processed for RNA extraction as described below. The efficiency of RNA fractionation was assessed by quantitative polymerase chain reaction (qPCR) using GAPDH as a marker for the cytoplasmic fraction and 45S pre-rRNA for the nuclear fraction.

### RNA extraction

RNA extraction was performed with Maxwell RSC simplyRNA Tissue Kit (Promega) following the manufacturer’s instructions with DNaseI treatment. DNA contamination was assessed after each extraction with a qPCR for genomic DNA on RNA samples.

### cDNA synthesis and qPCR analysis

Reverse transcription was performed with SuperScript IV First-Strand Synthesis System (Thermo Fisher Scientific) with random hexamers. Relative expression was determined by qPCR using SYBR Green PCR master mix (Thermo Fisher Scientific) according to the manufacturer’s instructions using the following amplification conditions: 95 °C 10 min; 45 cycles of 95 °C 15 s, 57.5 °C 20 s and 72 °C 25 s. Primers used in qPCR analyses are summarized in Supplementary Table 2. Relative expression was analysed by the comparative ΔΔCt method.

### RNA-seq

The quantity, quality and integrity of the RNA samples were assessed using Bioanalyzer 5400 (Agilent Technologies). After isolation, RNA was sent to Novogene for sequencing. In brief, poly(A) RNA was enriched with oligo d(T) magnetic beads. Subsequently, mRNA was randomly fragmented, and cDNA synthesis proceeded through the use of random hexamers and the reverse transcriptase enzyme. After purification, the resulting products underwent end-repair, A-tailing and adapter ligation steps. Fragments of the appropriate size were enriched by PCR, where indexed P5 and P7 primers were introduced, and final products were purified. The library was checked with Qubit 2.0 and real-time PCR for quantification and bioanalyzer Agilent 5400 for size distribution detection. Quantified libraries were pooled and sequenced on Illumina NovaSeq 6000/NovaSeq X Plus platforms. The qualified libraries were sequenced using next-generation sequencing based on Illumina’s sequencing-by-synthesis technology, detection by fluorescence of the nucleotide added during the synthesis of the complementary chain, and in a parallelized and massive way. The strategy was paired-end 150 bp.

### Analysis of IR from RNA-seq

Vast-tools v2.5.1^148^ (https://github.com/vastgroup/vast-tools) was used to calculate PIR and transcripts per million (TPM) values. Reads mapping to mid-intron sequences and balanced counts of reads aligning to upstream and downstream exon–intron sequences were used to evaluate IR levels. PIR was measured as a percentage of mean retention reads over the sum of retained and spliced intron reads. Raw values were filtered based on reported quality scores, requiring at least 15 total reads per event and absence of a positive result (*P* < 0.01) for the binomial test for upstream/downstream junction read balance. Additional filtering was applied to retain introns with ≥5 non‑missing values across RNA-seq datasets after quality filtering and a minimum nuclear expression of 5 TPM. For analyses of IR kinetics after transcriptional inhibition, events with both cytoplasmic untreated replicates showing PIR values ≥99% were excluded. For the analysis of nuclear and cytoplasmic RNA stability, RNAs expressed at a minimum of 7 TPM in both compartments were kept. This threshold was chosen because, among IR-mRNAs, RAD52 exhibited the lowest cytoplasmic abundance as assessed by smRNA FISH (mean of one molecule in the cytoplasm per cell), which corresponded to 7 TPMs in the cytoplasm. This ensured that RNAs included in the stability analysis were reliably detected in both compartments and avoided bias from low-abundance RNAs where fluctuations in read counts could disproportionately affect stability measurements.

IR stability classes were classified based on IR kinetics at the intron-event level, such that nuclear PIR values at each timepoint were normalized to the nuclear PIR level of the same intron in untreated cells. Introns were considered classifiable if they showed basal nuclear retention in untreated samples (mean nuclear PIR ≥30) and replicate support in untreated nuclei (PIR ≥25 in both replicates when available). Stable introns were defined as those retaining at least 50% of their initial nuclear PIR signal at 0.5 h, 2 h and 4.5 h after transcriptional inhibition, with absolute nuclear PIR values of at least 25 at each treatment timepoint. Unstable classes were defined according to the first timepoint at which normalized nuclear PIR dropped below 50% of their initial nuclear PIR. Introns with untreated nuclear PIR below 30 were assigned to the no IR group. The analysis was repeated using an untreated nuclear PIR cutoff of ≥50 while retaining the same stability criteria based on normalized PIR kinetics. Transcripts were assigned to an LL-IR-RNA group if they contained at least one RI classified as stable at 4.5 h. For transcripts containing multiple RIs, transcript- or gene-level assignment reflects the most stable RI event. GO analysis was performed using WebGestalt^149^.

### Genome assembly used in the downstream tasks

GENCODE Human genome release 29 was used in all downstream tasks. Genome primary assembly was used to extract intronic sequences and respective flanks (https://ftp.ebi.ac.uk/pub/databases/gencode/Gencode_human/release_29/GRCh38.primary_assembly.genome.fa.gz). Comprehensive annotation GTF was used to look-up boundaries of transcript bodies (https://ftp.ebi.ac.uk/pub/databases/gencode/Gencode_human/release_29/gencode.v29.primary_assembly.annotation.gtf.gz).

### ENCORE dataset

All eCLIP narrowPeak files for each RBP were retrieved from the ENCODE portal (ENCORE project) (https://www.encodeproject.org/encore-matrix/?type=Experiment&status=released&internal_tags=ENCORE&searchTerm=ENCORE). For downstream analyses, we summarized RBP peaks per intron and normalized by the intron’s length. Normalized peak counts were then used to generate heatmaps across spliced/retained and stable/unstable intron classes to check for RBP peak enrichment in one class versus the other.

### Estimation of half-lives at intron and gene level

Cells were sampled at 0, 0.5, 2 and 4.5 h after transcription termination. For each cellular compartment (nuclear/cytoplasmic) and replicate (A and B), gene-level abundance (TPMs) and PIR were quantified with vast-tools (see above). HLs were estimated separately at gene level using TPM measurements and at intron level using PIR measurements.

In both cases, the first timepoint zero (*T*_0_) was isolated, requiring both replicates present. Then, all post-*T*_0_ timepoints were normalized by their matching *T*_0_ within the same replicate, yielding normalized *C*(*t*)/C(*T*_0_) values (TPMs in case of genes and PIR in case of introns), whereas *T*_0_ was set to 1.

We assume first-order decay after transcription inhibition. Thus, for each compartment separately and for each gene/intron we estimated the decay rate by fitting a log-linear regression with ordinary least squares over both replicates, using scipy.stats.linregress(): $\log {C}_{\rm{norm}}\left(t\right)=\alpha +\beta t.\,$The HL was calculated from the decay constant k=−β as

$${\rm{HL}}\,=\frac{\mathrm{ln}\,2}{k}$$

The HL’s standard error estimate was propagated from the slope’s standard error SE:

$${\mathrm{SE}}_{\mathrm{HL}}\approx \frac{\mathrm{ln}2}{{k}^{2}}{\mathrm{SE}}_{\beta }.$$

After computing compartment-specific intron and gene HLs, we applied three filters, requiring that

1. ${C}_{\rm{norm}}({T}_{1})$ > 0, to remove cases below our detection capabilities;
2. HL >0;
3. HLs are filtered by relative uncertainty, ensuring that the relative HL error

$$r\,=\,\frac{|{\rm{SE}}_{\rm{HL}}|}{\rm{|HL|}}$$

is below the fourth quartile of the *r* distribution.

HL estimates exceeding 12 h—reflecting a relatively ‘flat’ exponential decay and thus highly stable RIs—were set to the limit’s value, consistent with previous studies^150^, as HL cannot be reliably determined in such cases. Finally, for machine learning modelling, single-intron HLs are normalized to the HL of the corresponding full-length gene, to account for differences arising from the overall stability of the transcript.

### Prediction of IR and intron HL with the Parnet foundation model

#### Parnet model

Parnet is a multitask sequence-to-signal deep learning model of protein–RNA interactions, extending our original RBPNet framework^82^. In brief, RBPNet is a dilated convolutional network that maps RNA sequences directly to single-nucleotide crosslink profiles from raw CLIP-seq data, trained on all regions with significant crosslink enrichment signal, without requiring peak calling. By modelling both experimental signal and control background for each RBP experiment separately, it disentangles protein-specific binding from technical noise. Trained on hundreds of thousands of regions across and for each of the ~200 RBPs from ENCORE, RBPNet learns binding profiles and encodes a functional representation of RNA sequences in its embedding space. Parnet builds on this foundation by generalizing to multiple RBPs simultaneously, leveraging the learned embeddings as transferable RNA representations. In practice, this means that Parnet encodes the sequence rules of the RBP interactome for a given RNA, enabling downstream tasks such as RNA classification and modelling RNA processes, either through frozen embeddings or with additional fine-tuning layers.

For predicting IR and intron HL from sequence, we used Parnet (release 0.3.0, 21M parameters; https://github.com/marsico-lab/parnet).

#### Parnet fine-tuning and class activation mapping (CAM) feature importance

Coordinates of alternative splicing events for *Homo sapiens* (hg38) were obtained from VastDB (https://vastdb.crg.eu/downloads/hg38/EVENT_INFO-hg38.tab.gz). Parnet was fine-tuned in two separate classification tasks: first, distinguishing retained from non-retained introns, with introns labelled as ‘spliced’ if PIR <0.1 and as ‘retained’ if PIR ≥30; and second, classifying unstable log(IR HL <−1.34) versus stable log(IR HL ≥−0.36) RIs (PIR ≥10%). Thresholds were defined based on the distributions shown in Fig. 3d and Extended Data Fig. 4a.

Training sequences were generated by extracting 256 bases from the 5′ and 3′ intron ends, including 50 bases of flanking exons to capture potential exonic splice enhancers and silencers, as most splice regulatory elements lie within a few hundred bases of splice sites. Fasta sequences were extracted based on the selected coordinates with the Python library pysam (https://github.com/pysam-developers/pysam). Sequences on the negative strand were reverse complemented. The resulting set of intronic sequences was one-hot encoded into four nucleotide channels (A, C, G and T) and forward-passed through the model’s body. For short introns that did not span the full 256-base window, sequences were padded to 256 bases, with valid positions marked as 1 and padding as 0.

For this task, we used the core of the original Parnet model—its series of one-dimensional convolutional blocks with residual connections—wrapped in a ParnetBackbone class. This backbone converts one-hot encoded RNA sequences into a feature map that represents each position in the sequence. Valid positions are tracked using the binary mask defined above. On top of the backbone, we added two CAM attention heads, one for each intron end, based on the approach by Zhou et al.^151^. These attention heads learn to weight each position in the sequence according to its importance for the classification task. Weighted feature vectors are then pooled to create a single sequence-level representation, which is passed through a linear classifier to produce predictions. Finally, per-base attribution scores are computed to quantify the contribution of each nucleotide to the model’s decision, with positions outside the valid sequence ignored. The outputs from both 5′ and 3′ attention heads are combined in a small multilayer perceptron to produce the final prediction, the probability that an intron is retained/stable. Model training was performed using fivefold cross-validation. The checkpoint from the fold with the best validation performance was selected for downstream analyses.

#### Attribution score interpretation

To interpret the sequence elements underlying the model’s attribution scores we used tf-modisco lite (https://github.com/jmschrei/tfmodisco-lite), a motif discovery algorithm tailored for deep learning attribution scores, which given the attribution scores computed above for each sequence, aggregates them to highlight patterns or motifs in the different classes, hereafter called attribution motifs. We followed the standard procedures provided in the package documentation, setting motif_size, window_size and n_seqs to 31, 256 and 1,000, respectively. Only motifs with a minimum of 20 seqlets derived at 0.05 false discovery rate (FDR) were retained. To link enriched motifs across intron sequence classes to potential RBP binding sites, we used tfmodisco’s implementation of TomTom to query what is, to our knowledge, the largest and most up-to-date database of human RBP motifs^152^. In detail, every attribution motif identified by tf-modisco was compared with every motif in the database, recording the top three most likely matches. For visualization, we reported only RBP motifs that matched the attribution motif with a *q* value (FDR) less than 0.05.

#### Visualization of intron embeddings with Parnet

Embeddings from the model’s penultimate layer were extracted and average-pooled into three segments, 5′ end, middle and 3′ end, then concatenated and saved in an h5 file. For visualization, intron embeddings were projected into two dimensions using Uniform Manifold Approximation and Projection (UMAP; Python implementation)^153^. Default parameters were used except for the number of neighbours increased to 50, the distance metric set to cosine, and the minimum distance between neighbours set to 0.15.

### Quantification of RBP enrichment

The annotation of nuclear speckle-associated RBPs was obtained from RBP Image Database from the Lecuyer lab^93^, selecting RBPs with a quality score >1 that were detected in nuclear speckles in either HepG2 or K562 cell lines (Supplementary Table 3). To test whether peaks of nuclear speckle-associated RBPs (from ENCODE NarrowPeaks) are differentially enriched or depleted in spliced versus RIs, we fitted a linear model with three terms: intron class (spliced or retained), RBP group (speckle or non-speckle) and their interaction. A type II analysis of variance (ANOVA) was then used to assess whether the difference in mean peak counts between speckle and non-speckle RBPs depends on intron class—that is, whether this difference changes between retained and spliced introns.

### G-quadruplex motif enrichment

We used canonical G-quadruplex motif definitions, that is, (G{3,}[ATGCN]{1,7}){3,}G{3,}, which are widely employed in Potential G-Quadruplex-forming Sequences identification^154^. For the variant motifs with longer loops, we extended this definition accordingly, that is (G{3,}[ATGCN]{8,12}){3,}G{3,}. Motifs containing only two G-tetrads have also been observed to form stable G-quadruplex structures, for example, intralocked G4s with G_2_ tracts^155^. In summary, the following motif regular expressions were used to analyse G-quadruplex motif enrichment in retained/stable versus non-retained/unstable introns:

motifs = {

“G4_3t_short”: r’G{3,}[ACGT]{1,7}G{3,}[ACGT]{1,7}G{3,}[ACGT]{1,7}G{3,}’,

“G4_3t_long”: r’G{3,}[ACGT]{8,12}G{3,}[ACGT]{8,12}G{3,}[ACGT]{8,12}G{3,}’,

“G4_2t_short”: r’G{2,}[ACGT]{1,7}G{2,}[ACGT]{1,7}G{2,}[ACGT]{1,7}G{2,}’,

“G4_2t_long”: r’G{2,}[ACGT]{8,12}G{2,}[ACGT]{8,12}G{2,}[ACGT]{8,12}G{2,}’,

}

To test whether G-quadruplex motifs were enriched in different intron classes, we counted motif occurrences using pattern matching and modelled these counts with a Poisson regression. The model compared motif counts between classes while accounting for intron length. From the fitted model, we calculated how much more (or less) frequent the motifs were in each class and expressed these values as log_2_ rate ratios for plotting.

### ssDRIP-seq and bisulfite sequencing processing

To assess the enrichment of R-loops in retained versus non-retained introns, we downloaded and reprocessed publicly available ssDRIP-seq dataset^84^. The raw sequencing data can be accessed with BioProject ID PRJNA608890 (https://www.ncbi.nlm.nih.gov/bioproject/PRJNA608890). The dataset was processed as in the original publication. Namely, the reads were first trimmed with trim_galore (https://github.com/FelixKrueger/TrimGalore) and then mapped with STAR (https://github.com/alexdobin/STAR). The resulting bam files were further deduplicated with samtools (https://github.com/samtools/samtools). Finally, the locations of R-loops were called with macs3 software (https://github.com/macs3-project/MACS).

In addition, we downloaded and reprocessed bisulfite sequencing runs available under the same BioProject ID, namely SRR1118540, following the processing steps taken by the authors of the original publication. In detail, the reads were first trimmed with trim_galore then mapped and deduplicated with Bismark (https://github.com/FelixKrueger/Bismark). Finally, Bismark’s command bismark_methylation_extractor was used to extract methylation calls.

The shell scripts to execute the processing as well as the exact software versions are available via GitHub (see ‘Code availability’ section).

### MFE calculation

The MFE of RNA secondary structures was calculated using the ViennaRNA Python API, following the provider’s standard procedure. For each intronic sequence (including 50-bp flanks), the MFE of the predicted optimal structure was obtained and then normalized by intron length for downstream analysis^156^.

### Analysis of detained introns

Before downstream analysis, coordinates of detained introns from Boutz et al.^17^ were lifted over from hg19 to hg38 version of human genome assembly using liftOver tool provided by UCSC (https://genome.ucsc.edu/cgi-bin/hgLiftOver). Then, the coordinates of detained introns from Boutz et al.^17^ and Braun et al.^18^ were intersected with the introns from the vast-tools database, selecting the best possible match between two pairs of intronic coordinates. Of note, the mapping was successful for 11,729 out of 13,290 and 3,750 out of 3,968 introns, respectively. The mapped intron IDs are provided in Supplementary Table 4.

### smRNA FISH and IF

smRNA FISH was performed as described previously^147,157,158^. In brief, up to 48 tiled oligonucleotides targeting intronic and exonic regions of candidate transcripts were custom designed using Stellaris online RNA FISH Probe Designer (LGC Biosearch Technologies). Of note, due to small intron size, we tiled oligonucleotides across two RIs of METTL3 to image the IR-RNA; as a result, the two introns cannot be reliably distinguished as independent species. The imaged RI of RAD52 was selected based on probe-design feasibility and corresponds to unstable 4.5 h RI, whereas RAD52 harbours at least one LL-RI that was too short for reliable imaging. Probe labelling was carried out as previously described^159^. For each target, up to 30 oligonucleotides (200 µM in nuclease-free water; Eurofins) were pooled into a single master mix and labelled with either 5-Propargylamino-ddUTP-ATTO-647N (Jena Biosciences) or 5-propargylamino-ddUTP-ATTO-550 (Jena Biosciences). For labelling, 5 µl of the oligonucleotide master mix was combined with 1 µl ddUTP-ATTO-647N or ddUTP--ATTO-550, 0.3 µl terminal deoxynucleotidyl transferase (TdT, 20 U µl^−1^), 3 µl TdT buffer (Thermo Fisher Scientific) and 5.7 µl water in a PCR tube. Reactions were incubated at 37 °C for 16 h in a PCR thermocycler with the lid set to 40 °C; an additional 0.3 µl of fresh TdT enzyme was added after 8 h. Labelled oligonucleotides were purified using the Oligo Clean & Concentrator kit (Zymo Research) according to the manufacturer’s instructions. Labelling efficiency was assessed by absorbance at 646 nm (ATTO-647N) or 554 nm (ATTO-550). Oligonucleotide concentrations were calculated using Beer’s law, and probes were diluted to a final stock concentration of 12.5 µM in water, which was used at a 1:100 dilution for hybridization.

Cells were seeded on vitronectin-coated (for hiPS cells) or fibronectin-coated (for HUVECs) 18-mm round glass coverslips. On the day of smRNA FISH, coverslips were washed twice with PBS, fixed with 3.7% formaldehyde in PBS for 10 min at room temperature, washed twice with PBS and incubated in 70% ethanol at 4 °C for a minimum of 1 h. Coverslips were washed with 1 ml of wash buffer A (nuclease-free 2× SSC, Thermo Fisher Scientific; 10% deionized formamide, Sigma-Aldrich) for 5 min at room temperature. Cells were hybridized with 80 µl of hybridization buffer (10% dextran sulfate in nuclease-free 2× SSC, 10% deionized formamide) containing smRNA FISH probes (1:100) overnight (ON) at 37 °C in a humid chamber.

The following day, coverslips were washed with 1 ml of wash buffer A for 30 min at 37 °C, followed by a second wash with wash buffer A containing Hoechst DNA stain (1:1,000; Thermo Fisher Scientific) for 30 min at 37 °C. Coverslips were washed with 1 ml of 2× SSC for 5 min at room temperature, equilibrated for 5 min in base glucose buffer (2× SSC, 0.4% glucose solution, 20 mM Tris, pH 8.0, in RNase-free water) and then incubated in base glucose buffer supplemented with a 1:100 dilution of glucose oxidase (stock 3.7 mg ml^−1^, Sigma-Aldrich) and catalase (stock 4 mg ml^−1^, Sigma-Aldrich) for 15 min at room temperature. Finally, coverslips were mounted with ProLong Glass Antifade Mountant (Thermo Fisher Scientific) on glass slides before image acquisition.

Coverslips for combinatorial IF and smRNA FISH were processed as described above, with primary antibodies added to the hybridization buffer: anti-SC35 antibody (1:1,500, #s4045, Clone SC-35, ascites fluid, Sigma-Aldrich), anti-RBM25 antibody (1:300, #25297-1-AP, Proteintech), anti-SON antibody (1:300, #83787-5-RR, Clone 240845B7, Proteintech) and anti-PCNA antibody (1:200, #HPA030522, Atlas Antibodies). The first wash with wash buffer A following overnight hybridization contained the appropriate Alexa Fluor 488-conjugated secondary antibody: anti-mouse IgG (1:1,000, #ab150113, Abcam) or anti-rabbit IgG (1:1,000, A-11008, Invitrogen).

For STED, high-precision glass coverslips were used (Hecht Assistent, thickness 170 ± 5 µm), DNA stain was omitted, and the secondary antibody was Abberior STAR 460L-conjugated anti-mouse IgG (1:1,000; #ST460L-1001, Abberior).

### Wide-field microscopy and image analysis

smRNA FISH with or without IF *z*-stacks (200 nm step size) were acquired using the Nikon ECLIPSE Ti2 widefield microscope with a Nikon Plan Apo λ ×100/1.45-numerical aperture oil objective lens and a Nikon DS-Qi2 camera. Maximum intensity projections were generated using Nikon software (NIS-Elements version 5.11.03) and deconvolved in ImageJ (version 2.14.0/1.54f) using DeconvolutionLab2 plugin (version 2.1.2)^160^. Quantification of RNA FISH signal was performed using the ‘Big-FISH’ v0.6.2 Python package^161^ or manually when the number of RNA spots was low. RNA spots were assigned to nuclear or cytoplasmic compartments based on Hoechst segmentation. Intron-retained RNA spots were identified by spatial overlap of exon and intron smRNA FISH signals, and spliced RNA for the RI by exon signal without co-localizing intron. Transcription sites were identified based on overlap between efficiently spliced intron signals and exon signals (for Fig. 4j and Extended Data Fig. 8b) or by high-intensity nascent RNA foci characteristic of active transcription (for Extended Data Fig. 8b; DNMT3B and LAMA5). Nuclear transcript fraction was quantified as the fraction of exon smRNA FISH spots detected in the nucleus relative to the total number of exon spots per cell, calculated as *N*_exon,nuc_/(*N*_exon,nuc_ + *N*_exon,cyt_), where *N*_exon,nuc_ and *N*_exon,cyt_ denote the numbers of exon spots detected in the nucleus and cytoplasm, respectively. Cytoplasmic transcript fraction was defined analogously as *N*_exon,cyt_/(*N*_exon,nuc_ + *N*_exon,cyt_). Nuclear PIR was calculated as *N*_RI,nuc_/*N*_exon,nuc_, and total PIR as *N*_RI,nuc_/(*N*_exon,nuc_ + *N*_exon,cyt_), where *N*_RI,nuc_ denotes the number of nuclear IR-RNA spots.

The distances of intron smRNA FISH signals to the nearest nuclear speckle were measured in three dimensions in Imaris (Bitplane, version 11.0) using the ‘Shortest Distance to Surfaces’ function. RNA FISH signals were segmented as spots using the Imaris ‘Spots’ module. SC35 or U2 signals were segmented independently as three-dimensional (3D) surfaces representing the SC35-positive nuclear speckle core and the U2-positive nuclear speckle shell, respectively, using the Imaris ‘Surface’ module. For each RNA spot, the shortest distance from its centroid to the nearest segmented surface was computed using the ‘Shortest Distance to Surfaces’ function. Distances were calculated separately relative to the SC35 surface and to the U2 surface. For each segmented structure, distances were negative when RNA spots were located within the segmented volume and positive when located outside the respective surface. Values close to 0 indicate localization at the surface interface.

To define distances expected under random positioning, random spots of the same size as smRNA FISH spots were generated in Imaris, and their distances to nuclear speckles were extracted using the same workflow. For each RNA molecule, its distance was expressed as its percentile relative to the random mixture reference. Under random positioning, these percentiles are expected to be uniformly distributed. Nuclear speckle proximity was tested using a one-sided Kolmogorov–Smirnov test comparing the RNA percentile distribution to a uniform distribution, and corrected for multiple testing across RNAs using the Benjamini–Hochberg procedure^162^ separately for SC35 and U2. Alongside *P* values, effect sizes are reported as the shift in median and mean distance relative to the random mixture expectation and as fold enrichment within 0.1 µm of nuclear speckles in Supplementary Tables 1 and 5.

For cell cycle-resolved smRNA FISH analyses, cell cycle stage was assigned at single-cell resolution using PCNA IF together with nuclear morphology and size. Early, mid and late S-phase cells were identified by characteristic PCNA replication foci^145^. G1 and G2 cells, both lacking PCNA foci, were distinguished by nuclear size, consistent with their 2N and 4N DNA content. Mitosis was identified by condensed chromatin morphology. Early G1 cells were identified as recently divided daughter-cell pairs with mirrored nuclear morphology and absent or early-onset transcriptional foci in exon/intron smRNA FISH. These annotations were used for downstream quantification.

### STED imaging

STED imaging was performed as previously described with several changes^163^. Image stacks were acquired on a Leica Stellaris 8 STED FALCON system using a 100× HC PL APO CS2 1.4-numerical aperture oil immersion objective. The samples were excited with a second-generation Leica pulsed White Light Laser at 553, 644 and 449 nm (for the long Stokes-shift dye) and detected using HyDS and HyDX detectors. For STED imaging of the three fluorophores of interest, the pulsed 775-nm STED laser was used and the STED laser intensity was set to 10%. The field of view was optimized for the ideal number of cells and the pixel dwell time was kept between 2.8 μs and 3 µs with a pixel size of 35 nm, and the scan speed was set to 200 Hz. The 3D *z*-stack acquisition was performed in a line-by-line fashion for each channel in the sequence with a *z*-stack slice height of 183 nm. The 3D stacks were visualized in Imaris (Bitplane).

### Statistics and reproducibility

In figures and figure legends, asterisk notation was used corresponding to: *P < 0.05, **P ≤ 0.01 and ***P ≤ 0.001. *P* values were adjusted using the FDR procedure (also known as Benjamini–Hochberg procedure)^162^, unless otherwise stated in the figure legend where no multiple tests were conducted. Test names and statistics, sample sizes and *P* values are reported in Supplementary Table 5.

In Fig. 3f, to test whether the means of nuclear speckle-associated RBP peaks are differentially enriched (or depleted) in retained/spliced and stable/unstable introns, we fit a simple linear model with three coefficients: intron class, RBP group and the interaction of the two. Then, we applied a type II ANOVA to test for significance of differences between means of the groups. In this formulation, ANOVA answers the question whether the difference in peak count means between speckle and non-speckle RBPs depends on the intron class, that is, whether the said difference varies depending on whether the intron is retained or spliced.

No statistical method was used to predetermine sample size. Sample sizes for smRNA FISH and imaging experiments were chosen based on standard practice in the field and prior studies using similar approaches. Sample sizes for smRNA FISH quantifications were considered sufficient when consistent trends were observed across individual cells, imaging fields and independent experiments. All experiments were performed with a minimum of three independent replicates, unless otherwise stated in the figure legends. smRNA FISH with ActD-treated samples was performed twice and was considered sufficient given the high concordance between replicates, individual cells and consistency with vast-tools results. Validation of SRSF7 by smRNA FISH was performed in two independent experiments, as it was a validation of previously published findings. No data were excluded from the analyses, except as described in the Methods (for example, quality-control filtering, RNA-seq and intron-level filtering). The experiments were not randomized; samples were assigned to experimental groups based on the experimental design and treatment conditions. Data collection and analysis were not performed blind to the conditions of the experiments.

### Reporting summary

Further information on research design is available in the Nature Portfolio Reporting Summary linked to this article.

## Online content

Any methods, additional references, Nature Portfolio reporting summaries, source data, extended data, supplementary information, acknowledgements, peer review information; details of author contributions and competing interests; and statements of data and code availability are available at https://doi.org/10.1038/s41556-026-02040-5.

## Supplementary information

Reporting Summary

Peer Review File

Supplementary Table Supplementary Tables 1–5. Spreadsheet workbook containing all supplementary tables, with each table provided as a separate sheet.

## Source data

Source Data Figs. 1–7 and Extended Data Fig. 1–10 Source numerical/statistical data for Figs. 1–7 and Extended Data Figs. 1–10, provided in separate worksheet tabs.

## Data Availability

Raw RNA-seq data and processed data that include PIR and TPM values obtained through vast-tools have been uploaded to GSE327794. Publicly available datasets used in this study include TSA-seq data from GSE81553 (ref. ^109^), log_2_FC of enrichment with nuclear speckle markers from ARTR-seq^74^, ssDRIP-seq and bisulfite sequencing data from BioProject PRJNA608890 (ref. ^84^), eCLIP narrowPeak files from the ENCODE portal (ENCORE project) (https://www.encodeproject.org/encore-matrix/?type=Experiment&status=released&internal_tags=ENCORE&searchTerm=ENCORE) and detained introns from Boutz et al.^17^ and Braun et al.^18^. The mapped detained intron IDs are provided in Supplementary Table 4. A list of nuclear speckle-associated RBPs was obtained from RBP Image Database from the Lecuyer lab^93^ and is provided in Supplementary Table 3. Source data are provided with this paper.

## Code availability

Code to fine-tune the Parnet foundation model, compute and interpret model attribution scores, along with the scripts and notebooks to reproduce the results, is available via GitHub at https://github.com/marsico-lab/IR_iPSCs as well as via Zenodo at https://doi.org/10.5281/zenodo.21135982 (ref. ^164^).
