# Supplementary material for: The periphery of nuclear speckles defines a spatially and temporally regulated compartment of long-lived intron-retained RNAs that resolves during mitosis
Source: Nat Cell Biol. 2026 Sep 9;28(9):1857–74. doi: 10.1038/s41556-026-02040-5 (PMC13561874; doi:10.1038/s41556-026-02040-5)
Supplement: Supplementary file 1 [file 41556_2026_2040_MOESM1_ESM.pdf]

nature portfolio

Corresponding author(s): Dumbovic, Gabrijela

Last updated by author(s): Jun 25, 2026

Reporting Summary

Nature Portfolio wishes to improve the reproducibility of the work that we publish. This form provides structure for consistency and transparency in reporting. For further information on Nature Portfolio policies, see our Editorial Policies and the Editorial Policy Checklist.

Statistics

For all statistical analyses, confirm that the following items are present in the figure legend, table legend, main text, or Methods section.

n/a	Confirmed	
☐	☒	The exact sample size (*n*) for each experimental group/condition, given as a discrete number and unit of measurement
☐	☒	A statement on whether measurements were taken from distinct samples or whether the same sample was measured repeatedly
☐	☒	The statistical test(s) used AND whether they are one- or two-sided<br>*Only common tests should be described solely by name; describe more complex techniques in the Methods section.*
☒	☐	A description of all covariates tested
☐	☒	A description of any assumptions or corrections, such as tests of normality and adjustment for multiple comparisons
☐	☒	A full description of the statistical parameters including central tendency (e.g. means) or other basic estimates (e.g. regression coefficient) AND variation (e.g. standard deviation) or associated estimates of uncertainty (e.g. confidence intervals)
☐	☒	For null hypothesis testing, the test statistic (e.g. *F*, *t*, *r*) with confidence intervals, effect sizes, degrees of freedom and *P* value noted<br>*Give P values as exact values whenever suitable.*
☒	☐	For Bayesian analysis, information on the choice of priors and Markov chain Monte Carlo settings
☒	☐	For hierarchical and complex designs, identification of the appropriate level for tests and full reporting of outcomes
☐	☒	Estimates of effect sizes (e.g. Cohen's *d*, Pearson's *r*), indicating how they were calculated

Our web collection on statistics for biologists contains articles on many of the points above.

Software and code

Policy information about availability of computer code

Data collection

Imaging: Nikon ECLIPSE Ti2 widefield microscope with a Nikon Plan Apo λ ×100/1.45-numerical aperture oil objective lens and a Nikon DS-Qi2 camera. Maximum intensity projections were generated using Nikon software (NIS-Elements version 5.11.03) and deconvolved in ImageJ (version 2.14.0/1.54f) using DeconvolutionLab2 plugin (version 2.1.2). Distances between intron smRNA FISH signals and SC35 or U2 surfaces were obtained in Imaris (Bitplane, version 11.0) using the "closest exterior surface distance" function. FISH signals were first detected and segmented as spots using the Imaris "Spots" module, and then Shortest Distance to Surfaces option was applied.

STED: image stacks were acquired on a Leica Stellaris 8 STED FALCON system using a 100x HC PL APO CS2 1.4 NA oil immersion objective.

RNA-seq: Illumina Novaseq 6000/Novaseq X Plus platforms.

The trained Parnet 21M model weights were downloaded from the parnet repository (release 0.3.0, 21M parameters; https://github.com/marsico-lab/parnet).

ENCORE dataset: Narrow RBP eCLIP peaks were downloaded from ENCORE portal (https://www.encodeproject.org/encore-matrix/?type=Experiment&status=released&internal_tags=ENCORE)

Data analysis

Image analysis: ImageJ/Fiji version 2.14.0/1.54f. Quantification of RNA FISH signal was performed using "Big-FISH" v0.6.2 Python package or manually when the number of RNA spots was low.

Vast-tools v2.5.1 (https://github.com/vastgroup/vast-tools) was used to calculate PIR and TPM values.

Minimum free energy (MFE) of RNA secondary structures was calculated using the ViennaRNA version 2.7.0 Python API, following the provider's standard procedure. For each intronic sequence (including 50 bp flanks), the MFE of the predicted optimal structure was obtained and then normalized by intron length for downstream analysis.

ssDRIP-seq dataset was processed as in the original publication. The reads were trimmed with trim_galore version 0.6.10 (https://github.com/FelixKrueger/TrimGalore) and mapped with STAR 2.7.11b (https://github.com/alexdobin/STAR). The resulting bam files were deduplicated with samtools 1.23.1 (https://github.com/samtools/samtools). Locations of R-loops were called with macs3 software version 3.0.4 (https://github.com/macs3-project/MACS).

Bisulfite sequencing reads were trimmed with trim_galore 0.6.10 then mapped and deduplicated with Bismark 0.25.1 (https://github.com/FelixKrueger/Bismark). Bismark's command bismark_methylation_extractor was used to extract methylation calls.

Estimation of half-lives were generated by fitting a log-linear regression to the time-series data (Methods – Estimation of half-lives at intron and gene level). The code for that analysis can be found at https://github.com/marsico-lab/IR_iPSCs/blob/main/notebooks/2.hl_revision_workflow.ipynb jupyter notebook.

RBP peaks per intron were summarized and normalized by the intron length. Normalized peak counts were then used to generate heatmaps across spliced/retained and stable/unstable intron classes; to check for RBP peak enrichment in one class versus the other.

Code to fine-tune the Parnet foundation model, compute and interpret model attribution scores, along with the scripts, notebooks, and environments can be found at: https://github.com/marsico-lab/IR_iPSCs and was deposited on Zenodo at https://zenodo.org/records/21135983. For interrogation of attribution scores, tf-modisco lite version 2.4.0 (https://github.com/jmschrei/tfmodisco-lite) in a python 3.9 conda environment was used.

For manuscripts utilizing custom algorithms or software that are central to the research but not yet described in published literature, software must be made available to editors and reviewers. We strongly encourage code deposition in a community repository (e.g. GitHub). See the Nature Portfolio guidelines for submitting code & software for further information.

Data

Policy information about availability of data

All manuscripts must include a data availability statement. This statement should provide the following information, where applicable:

- Accession codes, unique identifiers, or web links for publicly available datasets
- A description of any restrictions on data availability
- For clinical datasets or third party data, please ensure that the statement adheres to our policy

ARTR-seq data was retrieved from Wu J et al., 2024. TSA-seq data was retrieved from the GEO accession number GSE81553 (Chen Y et al., 2018).

A list of previously annotated detained introns was retrieved from Boutz et al., 2015 and Braun et al., 2018.

A list of nuclear speckle-associated RBPs was obtained from RBP Image Database from the Lecuyer lab.

Enrichment of R-loops in retained versus non-retained introns was downloaded and reprocessed from the publicly available ssDRIP-seq dataset (Chen L et al., 2021). The raw sequencing data can be accessed with the following BioProject id PRJNA608890 (https://www.ncbi.nlm.nih.gov/bioproject/PRJNA608890).

Bisulphite sequencing runs are available under the same bioproject id (SRR1118540), following the processing steps taken by the authors of the original publication.

All eCLIP narrowPeak files for each RBP were retrieved from the ENCODE portal (ENCORE project) (https://www.encodeproject.org/encore-matrix/?type=Experiment&status=released&internal_tags=ENCORE&searchTerm=ENCORE).

The trained Parnet 21M model weights were downloaded from the parnet repository (release 0.3.0, 21M parameters; https://github.com/marsico-lab/parnet).

Raw RNA-seq data and processed data that include PIR and TPM values obtained through vast-tools are available in GEO under accession GSE327794.

Research involving human participants, their data, or biological material

Policy information about studies with human participants or human data. See also policy information about sex, gender (identity/presentation), and sexual orientation and race, ethnicity and racism.

Reporting on sex and gender	*Use the terms sex (biological attribute) and gender (shaped by social and cultural circumstances) carefully in order to avoid confusing both terms. Indicate if findings apply to only one sex or gender; describe whether sex and gender were considered in study design; whether sex and/or gender was determined based on self-reporting or assigned and methods used. Provide in the source data disaggregated sex and gender data, where this information has been collected, and if consent has been obtained for sharing of individual-level data; provide overall numbers in this Reporting Summary. Please state if this information has not been collected. Report sex- and gender-based analyses where performed, justify reasons for lack of sex- and gender-based analysis.*
Reporting on race, ethnicity, or other socially relevant groupings	*Please specify the socially constructed or socially relevant categorization variable(s) used in your manuscript and explain why they were used. Please note that such variables should not be used as proxies for other socially constructed/relevant variables (for example, race or ethnicity should not be used as a proxy for socioeconomic status). Provide clear definitions of the relevant terms used, how they were provided (by the participants/respondents, the researchers, or third parties), and the method(s) used to classify people into the different categories (e.g. self-report, census or administrative data, social media data, etc.)*

Population characteristics	*Describe the covariate-relevant population characteristics of the human research participants (e.g. age, genotypic information, past and current diagnosis and treatment categories). If you filled out the behavioural & social sciences study design questions and have nothing to add here, write "See above."*
Recruitment	*Describe how participants were recruited. Outline any potential self-selection bias or other biases that may be present and how these are likely to impact results.*
Ethics oversight	*Identify the organization(s) that approved the study protocol.*

Please provide details about how you controlled for confounding variables in your analyses.

Note that full information on the approval of the study protocol must also be provided in the manuscript.

Field-specific reporting

Please select the one below that is the best fit for your research. If you are not sure, read the appropriate sections before making your selection.

☒ Life sciences ☐ Behavioural & social sciences ☐ Ecological, evolutionary & environmental sciences

For a reference copy of the document with all sections, see nature.com/documents/nr-reporting-summary-flat.pdf

Life sciences study design

All studies must disclose on these points even when the disclosure is negative.

Sample size	No statistical methods were used to predetermine sample size. Sample sizes for smRNA FISH and imaging experiments were chosen based on standard practice in the field and prior studies using similar approaches. Sample sizes for smRNA FISH quantifications were considered sufficient when consistent trends were observed across individual cells, imaging fields and independent experiments. All experiments were performed with a minimum of three independent biological replicates, unless otherwise stated in the figure legends. smRNA FISH following ActD treatment was performed in two independent experiments and was considered sufficient because of the high concordance between replicates and consistency with the vast-tools results. Validation of SRSF7 by smRNA FISH was performed in two independent experiments, as it was a validation of previously published findings. For sequencing-based and computational analyses, sample sizes correspond to the number of introns, genes, transcripts, passing the predefined filtering criteria; no additional statistical sample-size calculation was performed. Samples were allocated to experimental groups according to the experimental design, treatment condition, time point, cell-cycle stage or computational class definition. Random allocation was not relevant for sequencing-based and computational analyses because groups were defined by predefined biological or computational criteria. Imaging fields and cells were selected randomly. Exact sample numbers for each experiment or analysis are provided in the corresponding figure legends and Source Data.
Data exclusions	No data were excluded from the analyses, except as described in the Methods (for example, quality-control filtering, RNA-seq and intron-level filtering).
Replication	Data reproducibility was verified by performing independent biological and technical measurements.
Randomization	The experiments were not randomized; samples were assigned to experimental groups based on experimental design and treatment conditions. Imaging fields/cells were selected randomly.
Blinding	All experiments in any comparisons used cells grown in same culture conditions. The investigators were not blinded because experiments were designed and performed by the same person.

Reporting for specific materials, systems and methods

We require information from authors about some types of materials, experimental systems and methods used in many studies. Here, indicate whether each material, system or method listed is relevant to your study. If you are not sure if a list item applies to your research, read the appropriate section before selecting a response.

Materials & experimental systems

n/a	Involved in the study	
☐	☒	Antibodies
☐	☒	Eukaryotic cell lines
☒	☐	Palaeontology and archaeology
☒	☐	Animals and other organisms
☒	☐	Clinical data
☒	☐	Dual use research of concern
☒	☐	Plants

Methods

n/a	Involved in the study	
☒	☐	ChIP-seq
☒	☐	Flow cytometry
☒	☐	MRI-based neuroimaging

Antibodies

Antibodies used	Monoclonal anti SC-35 antibody (Sigma Aldrich, 1:1500); Alexa Fluor® 488 anti-Mouse IgG (Abcam, 1:1000); Goat anti-Rabbit IgG Alexa Fluor™ 488 (Invitrogen, 1:1000); STAR 460L anti-Mouse IgG (Abberior, 1:1000); RBM25 Polyclonal antibody (Proteintech, 1:300); Anti-PCNA Antibody (Atlas Antibodies, 1:200); SON Recombinant monoclonal antibody (Proteintech, 1:300)
Validation	Monoclonal anti SC-35 antibody (Sigma Aldrich, #s4045, Clone SC-35, ascites fluid): The manufacturer validated the antibody for ELISA, EM, ICC, IF, IP use. The antibody was purchased new and used per manufacturer's instructions in 1:1500 dilution. No further validation was performed.<br>https://www.sigmaaldrich.com/DE/en/product/sigma/s4045<br><br>Alexa Fluor® 488 anti-Mouse IgG (Abcam, #ab150113): The manufacturer validated the antibody for ICC/IF, IHC, flow cytometry and ELISA use. The antibody was purchased new and used per manufacturer's instructions in 1:1000 dilution. Further validation was performed by including a condition without the primary antibody.<br>https://www.abcam.com/en-us/products/secondary-antibodies/goat-mouse-igg-h-l-alexa-fluor-488-ab150113<br><br>Goat anti-Rabbit IgG (H+L) Cross-Adsorbed Secondary Antibody, Alexa Fluor™ 488 (Invitrogen, #A-11008): The manufacturer validated the antibody for ICC/IF, IHC, flow cytometry. The antibody was purchased new and used per manufacturer's instructions in 1:1000 dilution. Further validation was performed by including a condition without the primary antibody.<br>https://www.thermofisher.com/antibody/product/Goat-anti-Rabbit-IgG-H-L-Cross-Adsorbed-Secondary-Antibody-Polyclonal/A-11008<br><br>STAR 460L anti-Mouse IgG (Abberior, #ST460L-1001-500UG): The manufacturer validated the antibody for STED use. The antibody was purchased new and used per manufacturer's instructions in 1:1000 dilution. No further validation was performed.<br>https://abberior.shop/abberior-STAR-460L-goat-anti-mouse-IgG-500-ll-1-mg-ml<br><br>RBM25 Polyclonal antibody (Proteintech, #25297-1-AP): The manufacturer validated the antibody for WB, IHC, IF/ICC, IP, ELISA. The antibody was purchased new and used per manufacturer's instructions in 1:300 dilution. No further validation was performed.<br>https://www.ptglab.com/products/RBM25-Antibody-25297-1-AP.htm<br><br>PCNA Antibody (Atlas Antibodies, #HPA030522): The manufacturer validated the antibody for WB, IHC, IF/ICC. The antibody was purchased new and used per manufacturer's instructions in 1:200 dilution. No further validation was performed.<br>https://www.atlasantibodies.com/products/primary-antibodies/triple-a-polyclonals/anti-pcna-antibody-hpa030522/<br><br>SON Recombinant monoclonal antibody (Proteintech, #83787-5-RR, Clone 240845B7): The manufacturer validated the antibody for WB, IHC, IF/ICC, flow cytometry and ELISA. The antibody was purchased new and used per manufacturer's instructions 1:300 dilution. No further validation was performed.<br>https://www.ptglab.com/products/SON-Antibody-83787-5-RR.htm

Eukaryotic cell lines

Policy information about cell lines and Sex and Gender in Research

Cell line source(s)	Human iPSC WTC-11 (GM25256, male, Coriell Institute); HUVECs (c-12203, pool female/male, PromoCell)
Authentication	All cell lines were obtained from repositories with the corresponding certificate of analysis and used without further authentication.
Mycoplasma contamination	All cell lines were tested negative for mycoplasma.
Commonly misidentified lines<br>(See ICLAC register)	No commonly misidentified lines were used.

Plants

Seed stocks	*Report on the source of all seed stocks or other plant material used. If applicable, state the seed stock centre and catalogue number. If plant specimens were collected from the field, describe the collection location, date and sampling procedures.*
Novel plant genotypes	*Describe the methods by which all novel plant genotypes were produced. This includes those generated by transgenic approaches, gene editing, chemical/radiation-based mutagenesis and hybridization. For transgenic lines, describe the transformation method, the number of independent lines analyzed and the generation upon which experiments were performed. For gene-edited lines, describe the editor used, the endogenous sequence targeted for editing, the targeting guide RNA sequence (if applicable) and how the editor was applied.*
Authentication	*Describe any authentication procedures for each seed stock used or novel genotype generated. Describe any experiments used to assess the effect of a mutation and, where applicable, how potential secondary effects (e.g. second site T-DNA insertions, mosiacism, off-target gene editing) were examined.*
