# Supplementary material for: The periphery of nuclear speckles defines a spatially and temporally regulated compartment of long-lived intron-retained RNAs that resolves during mitosis
Source: Nat Cell Biol. 2026 Sep 9;28(9):1857–74. doi: 10.1038/s41556-026-02040-5 (PMC13561874; doi:10.1038/s41556-026-02040-5)
Supplement: Supplementary file 2 [file 41556_2026_2040_MOESM2_ESM.pdf]

nature portfolio

Peer Review File

The periphery of nuclear speckles defines a spatially and temporally regulated compartment of long-lived intron-retained RNAs that resolves during mitosis

Corresponding Author: Dr Gabrijela Dumbović

Version 0:

Decision Letter:

*Please delete the link to your author homepage if you wish to forward this email to co-authors.

Dear Dr Dumbović,

Your manuscript, "Nuclear speckle periphery organizes stable intron-retained RNAs into a distinct nuclear retention compartment that resolves during mitosis", has now been seen by 3 referees, who are experts in nuclear speckles (referee 1); RNA interactions referee 2); and splicing (referee 3). As you will see from their comments (attached below) they find this work of potential interest, but have raised substantial concerns, which in our view would need to be addressed with considerable revisions before we can consider publication in Nature Cell Biology.

Nature Cell Biology editors discuss the referee reports in detail within the editorial team, including the chief editor, to identify key referee points that should be addressed with priority, and requests that are overruled as being beyond the scope of the current study. To guide the scope of the revisions, I have listed these points below. We are committed to providing a fair and constructive peer-review process, so please feel free to contact me if you would like to discuss any of the referee comments further.

In particular, it would be essential to:

A- Further experimentally investigate the role of mitotic entry in the splicing of detained introns (Referee#3 pt 6 – paragraph beginning "Mitosis acting as a trigger for the splicing of these detained introns is a very interesting observation…".)
B- For inhibitor experiments used to manipulate nuclear speckle remodelling during mitosis, clarify how to distinguish between effects from inhibition of nuclear speckle remodelling versus altered SRSF phosphorylation (referee#1 pt 3)
C- Ensure to provide full methodological details and details of all statistical analyses used (all referees- ref#1 pts 6,7,9, ref#2 minor pt, ref#3 pts 5-6)

D- All other referee concerns pertaining to strengthening existing data, providing controls, methodological details, clarifications and textual changes, should also be addressed.

E- Finally please pay close attention to our guidelines on statistical and methodological reporting (listed below) as failure to do so may delay the reconsideration of the revised manuscript. In particular please provide:

- a Supplementary Figure including unprocessed images of all gels/blots in the form of a multi-page pdf file. Please ensure that blots/gels are labeled and the sections presented in the figures are clearly indicated.

- a Supplementary Table including all numerical source data in Excel format, with data for different figures provided as different sheets within a single Excel file. The file should include source data giving rise to graphical representations and statistical descriptions in the paper and for all instances where the figures present representative experiments of multiple independent repeats, the source data of all repeats should be provided.

We would be happy to consider a revised manuscript that would satisfactorily address these points, unless a similar paper is published elsewhere, or is accepted for publication in Nature Cell Biology in the meantime.

When revising the manuscript please:

- ensure that it conforms to our format instructions and publication policies (see below and https://www.nature.com/nature/for-authors).

- provide a point-by-point rebuttal to the full referee reports verbatim, as provided at the end of this letter.

- for any revision that includes light microscopy data, we ask our authors to please include a completed light microscopy reporting table https://www.nature.com/documents/Light_microscopy_reporting_table.xlsx to ensure the methods are described thoroughly. The table will be available to reviewers and ultimately published should the manuscript be accepted at the journal.

- provide the completed Reporting Summary (found here https://www.nature.com/documents/nr-reporting-summary.pdf). This is essential for reconsideration of the manuscript will be available to editors and referees in the event of peer review. For more information see http://www.nature.com/authors/policies/availability.html or contact me.

When submitting the revised version of your manuscript, please pay close attention to our href="https://www.nature.com/nature-portfolio/editorial-policies/image-integrity">Digital Image Integrity Guidelines. and to the following points below:

-- that unprocessed scans are clearly labelled and match the gels and western blots presented in figures.
-- that control panels for gels and western blots are appropriately described as loading on sample processing controls
-- all images in the paper are checked for duplication of panels and for splicing of gel lanes.

Finally, please ensure that you retain unprocessed data and metadata files after publication, ideally archiving data in perpetuity, as these may be requested during the peer review and production process or after publication if any issues arise.

Nature Cell Biology is committed to improving transparency in authorship. As part of our efforts in this direction, we are now requesting that all authors identified as ‘corresponding author’ on published papers create and link their Open Researcher and Contributor Identifier (ORCID) with their account on the Manuscript Tracking System (MTS), prior to acceptance. ORCID helps the scientific community achieve unambiguous attribution of all scholarly contributions. You can create and link your ORCID from the home page of the MTS by clicking on ‘Modify my Springer Nature account’. For more information please visit please visit www.springernature.com/orcid.

This journal strongly supports public availability of data. Please place the data used in your paper into a public data repository, or alternatively, present the data as Supplementary Information. If data can only be shared on request, please explain why in your Data Availability Statement, and also in the correspondence with your editor. Please note that for some data types, deposition in a public repository is mandatory - more information on our data deposition policies and available repositories appears below.

Please submit the revised manuscript files and the point-by-point rebuttal to the referee comments using this link:

Link Redacted

*This url links to your confidential home page and associated information about manuscripts you may have submitted or be reviewing for us. If you wish to forward this email to co-authors, please delete the link to your homepage.

We would like to receive a revised submission within six months.

We hope that you will find our referees' comments, and editorial guidance helpful. Please do not hesitate to contact me if there is anything you would like to discuss.

Best wishes,

Sabrya Carim

Sabrya Carim, PhD
(she/her/hers)
Senior Editor, Nature Cell Biology
Nature Portfolio

Springer Nature
The Campus, 4 Crinan Street, London N1 9XW, UK
sabrya.carim@springernature.com
https://orcid.org/0000-0001-9485-1938

Reviewers' Comments:

Reviewer #1 (Remarks to the Author):

In this manuscript, Bianya et al. propose that intron-detained RNAs derived from cell cycle–related genes are pooled at the periphery of nuclear speckles and undergo synchronous splicing at mitosis, thereby contributing to the elaborate regulation of cell cycle–related genes. The authors' observations on the relationship between intron-detained RNAs and nuclear speckles during the mitotic cell cycle are informative and potentially valuable for the readers of Nature Cell Biology.

However, I have two major concerns. First, the authors conclude that "our findings demonstrate that IR is not stochastic, but a regulated program…" (lines 101–104). This concept, however, is neither novel nor original to the authors. Multiple research groups in the RNA field have already proposed and substantiated this idea—commonly referred to as intron detention—over the past decade. The authors do not sufficiently acknowledge previously reported findings, nor do they cite several key papers in this area. Moreover, they introduce a new term for a splicing event already known as intron detention. Altogether, this gives the impression that prior work by other researchers has been underestimated and that the novelty and originality of the present study are overstated. Second, the authors attempt to verify that inhibition of nuclear speckle remodeling during mitosis alters intron detention. To this end, they use a CLK1 inhibitor. However, CLK1 is a well-known kinase that regulates splicing—including intron retention and intron detention—through phosphorylation of SR splicing factors. It is therefore unclear how the authors discriminate between the effects of CLK1 inhibition on nuclear speckle remodeling and its well-established roles in splicing regulation independent of nuclear speckles.

Overall, although I agree that this study potentially highlights the biological importance of intron detention in cell-cycle progression, its conceptual novelty is limited, and the methodology of the core experiments appears flawed in its current form. Additional details and further concerns are described below.

Major Points

1. Several relevant papers are not cited.
DOI: 10.1083/jcb.201107093 (the first report describing signal-induced post-transcriptional splicing of intron-detained RNAs in the nucleus)
DOI: 10.1080/15384101.2017.1337983 (germ cell differentiation–dependent regulation of intron detention)
DOI: 10.1101/gr.273904.120 (a comprehensive analysis of intron retention and detention in subnuclear compartments)
DOI: 10.15252/embj.2019102729 (a nuclear MLO that controls intron detention)
These papers should be cited and discussed in the context of the current study.

2. The analyses presented in Figures 1 and 2 are informative but conceptually not novel, as several groups have previously reported similar bioinformatic analyses examining features of detained introns such as functional enrichment, intron length, and GC content.
DOI: 10.1101/gad.247361.114
DOI: 10.1101/gad.247361.114
DOI: 10.1101/gr.273904.120
Some points highlighted by the authors (e.g., enrichment in cell-cycle and DNA damage–related genes) have already been described in these studies. The authors should discuss prior findings more fairly and explicitly compare their results with previously reported data. It is also important for the authors to clarify which aspects of their findings truly provide novel or advanced insights. Moreover, it is unclear why the authors introduce a new term, "LL-IR RNAs," for a class of RNAs widely known as intron-detained RNAs. Intron-detained RNAs are defined as partially spliced RNAs containing one or more introns that are stably retained in the nucleus and that undergo splicing upon specific stimulation and/or function as a regulatory buffer. This definition is conceptually and essentially identical to LL-IR RNAs.

3. The authors use CLK1 and DYRK3 inhibitors to manipulate nuclear speckle remodeling during mitosis. However, these kinases are well-established regulators of splicing—including intron detention—through phosphorylation of SR proteins and SR-related factors. In particular, multiple reports have shown that CLK1 activity modulates splicing of intron-detained RNAs independently of the cell cycle. Because SRSFs also localize broadly within the nucleoplasm and some are enriched in nuclear bodies other than speckles, it remains unclear how the authors distinguish whether the observed effects result from inhibition of nuclear speckle remodeling rather than from altered SRSF phosphorylation outside of speckles. The authors should clarify this point and cite more appropriate, up-to-date studies on CLK1 and its role in splicing regulation, including intron detention.

4. In the description of Figure 3, the authors should discuss previous findings that GC-rich introns and shorter introns are enriched in nuclear speckles.
DOI: 10.1016/j.molcel.2021.12.010 (reference 33)

5. In Figures 6A and 6B, the authors present only the ratio of exon to intron detention. To exclude the possibility that intron-detained RNAs are simply degraded, they should also show quantitative changes in the levels of spliced mRNAs.

6. In the Materials and Methods section, statistical procedures are specified only for Figure 3 and Supplementary Figures 3D and 4A. According to other figure legends, the authors repeatedly used t-tests for multiple comparisons. If the reported p-values were adjusted for multiple testing (e.g., using FDR or Bonferroni correction), the correction procedures should be specified in each legend or in the Methods section. If no corrections were applied, the authors should re-evaluate

significance using methods appropriate for multiple comparisons. Additionally, the type of statistical test used in Supplementary Figure 7 is not indicated.

7. The methods for the FISH experiments in Figures 5 and 6 are insufficiently described, making it difficult to understand how "nuclear RNA abundance" was quantified. It is also confusing that the terms "nuclear RNA abundance" and "nuclear RNA enrichment" are used interchangeably despite appearing to refer to the same measurement. The terminology should be unified and the quantification method clearly explained.

8. The list of speckle-associated RBPs used for the analysis in Figure 3 is not provided. It should be included in the supplementary materials.

Minor Points
9. In the smFISH protocol, the description "smRNA FISH exon/intron probes (1:100)" (line 977) is unclear because the stock concentration of the probes is not specified.

10. Some explanations are insufficient in the text and figures.
The significance of the long arrow in Figure 1E is not described.
The meaning of "UT" in Figure 2A is unclear.
"HL" (line 298) should be defined.

11. Several terms should be standardized for consistency:
SC35-rich domain / SC35 domain / SC35-dense interior
class / type
"Exon RNA FISH spots" (Figure 1D) vs. "Exon nuclear" (Figure 5), if they refer to the same measurement.

12. Typographical errors:
"mature RNA mRNA" (line 58), "untread" (line 183),
Duplicate sentence in Supplementary Figure 1 legend:
"Introns selected for smRNA FISH are marked with a purple asterisk."

Reviewer #2 (Remarks to the Author):

This is an impressive study with several elements of novelty. Although unspliced mRNA was found localized in nuclear speckles decades ago, the present findings extend this specifically to transcripts whose splicing is kinetically slow relative to the majority. The work is technically well executed, and the conclusions are strongly supported by the data. This paper reminds all of us in the nuclear organization and function field that we still have new things to learn.

The authors should consider the following points.

1. It would be good to emphasize that this peri-speckle localization is not at the nuclear bodies known as "paraspeckles". The authors could cite the fact that paraspeckles do not contain the SON protein and thus would not be captured by the designed proximity ligation method. They could also cite literature that pluripotent stems cells typically lack paraspeckles.

2. In the abstract, the phrase "and are released to the cytosol" could be rephrased to just "are released", since the nuclear envelope disassembles in mitosis (as do the speckles themselves) and thus everything that was intranuclear is "released".

3. Unless NCB allows speculation in the Abstract, the authors should eliminate the last sentence of the second paragraph. They are of course free to so speculate in the Discussion. The Abstract also seems much too long and could be shortened if the handling editor concurs.

4. Where in the nucleus splicing occurs was a very controversial issue, with some groups claiming it is co-transcriptional* and others saying it requires that the pre-mRNA be released from the transcription site and arrive at a speckle. The authors need not write a minireview on this but should mention this more in their Introduction and Discussion. They might also cite an important early paper in the field that demonstrated that if the transcriptional intensity of a gene is very high then splicing factors are recruited there in high concentrations and thus a speckle forms de novo (Huang and Spector, Genes Dev. 5: 2288-2302, 1991.

5. Line 528: "We hypothesize this mechanism ensures their inheritance by newly formed daughter cells and translation in early G1 without requiring new transcription, splicing and export, which can take tens of minutes to over an hour in mammalian cells." It would be good for the authors to see whether they can see long-lived IR spots in post-mitotic cells, e.g. neurons.

Minor point: Line 152-3 "Retained intron signals were frequently, but not necessarily, detected at or near transcription sites." One has to find the legend for Sup. Fig. 7 to see how transcription sites were detected in these experiments. This should be described in more detail in the methods.

* Although not a point the authors need to mention but something to always be borne in mind is that all three of the first pre-

mRNAs to be identified (β-globin, ovalbumin and the adenovirus tripartite leader) involved Northerns of oligo(dT)-cellulose selected RNA and thus their subsequent splicing was post-transcriptional.

Thoru Pederson and Paul D. Kaufman, UMass Chan Medical School

Reviewer #3 (Remarks to the Author):

This is a peer review of "Nuclear speckle periphery organizes intron-retained RNAs into a distinct nuclear retention compartment that resolves during mitosis" from Biayna et al. The authors begin by showing that intron retention is increased in the nucleus compared to the cytoplasm (fig 1). Using global transcriptional inhibition they show that a subset of retained introns in the nucleus are particularly stable (fig 2). They use a machine learning approach to link IR-RNAs with nuclear speckles through RBP motif enrichment (fig 3) which they then confirm by analzying TSA-seq and using smFISH (fig 4). However, they go on to show that nuclear speckles are not required to observe the intron-retention in that class of RNAs (fig 5). Interestingly, the authors demonstrate that mitosis is associated with the eventual splicing of the those RNAs (fig 6). Finally, the authors attempt to associate specific mitotic kinase activities with the intron retention (fig 6).

Comments
1. The manuscript contains two key findings. First, a subset of detained intron containing transcripts localize to the periphery of nuclear speckles. Second, mitosis is a trigger for splicing many of those transcripts.

2. Stable intron-containing transcripts that remain in the nucleus have been known for quite a while (Boutz et al 2015). Given that these early studies were also done in stem cells, the transcripts identified in this study should be compared with those previously identified.

3. It seems the conclusion of figure 1 is that RNAs in the nucleus tend to have more introns. Wouldn't this be the expected result based on first principles, since nuclear RNAs were likely more recently produced and may still be undergoing processing?

4. Actinomycin D is a pretty stressful treatment for cells and is likely triggering stress response pathways. This is a minor issue, but metbolic labeling of RNAs, perhaps with a pulse of 4sU would likely have been a more subtle approach.

5. The path from a machine learning model based on eCLIP data to the proposal that these transcripts are speckle associated is not clearly described.

6. The authors should clarify what negative distance to a speckle means (fig 4C and elsewhere). In addition, the peri-speckle assertion requires some sort of null distribution to compare to. I.E. If one chose random spots in the nucleus, what would the expected distribution of distances to nearest speckles look like.

7. It is not clear from the images shown if SON has been depleted by it's shRNA. Removal of both SON and SRRM2 is required to prevent speckle formation.

6. Mitosis acting as a trigger for the splicing of these detained introns is a very interesting observation. However, besides stating that they are using nocodazole, there is no description in the methods about how this is being done. Is this a nocodazole block for the cells labeled "M" and those labeled G2. Nocodazole is supposed to block the cell cycle at prometaphase, so I'm a bit confused about how the G2 cells are obtained. It would be supportive to use another method of synchronizing cells like a thymidine block so that cells could be tracked following release from the block to see if splicing in coincident with entry into M phase. Another prediction would provide needed support for the authors assertions would be that time spent in the thymidine block would lead to more accumulation of the unspliced nuclear transcripts, since you are delaying M phase entry. Given the importance of this finding to the novelty of the work, additional support and details for the role of mitotic entry is critical.

GUIDELINES FOR MANUSCRIPT SUBMISSION TO NATURE CELL BIOLOGY

READABILITY OF MANUSCRIPTS – Nature Cell Biology is read by cell biologists from diverse backgrounds, many of whom are not native English speakers. Authors should aim to communicate their findings clearly, explaining technical jargon that might be unfamiliar to non-specialists, and avoiding non-standard abbreviations. Titles and abstracts should concisely communicate the main findings of the study, and the background, rationale, results and conclusions should be clearly explained in the manuscript in a manner accessible to a broad cell biology audience. Nature Cell Biology uses British spelling.

MANUSCRIPT FORMAT – please follow the guidelines listed in our Guide to Authors regarding manuscript formats at

Nature Cell Biology.

TITLE – should be no more than 100 characters including spaces, without punctuation and avoiding technical terms, abbreviations, and active verbs..

AUTHOR NAMES – should be given in full.

AUTHOR AFFILIATIONS – should be denoted with numerical superscripts (not symbols) preceding the names. Full addresses should be included, with US states in full and providing zip/post codes. The corresponding author is denoted by: "Correspondence should be addressed to [initials]."

ABSTRACT AND MAIN TEXT – please follow the guidelines that are specific to the format of your manuscript, as listed in our Guide to Authors (http://www.nature.com/ncb/pdf/ncb_gta.pdf) Briefly, Nature Cell Biology Articles, Resources and Technical Reports have 3500 words, including a 150 word abstract, and the main text is subdivided in Introduction, Results, and Discussion sections. Nature Cell Biology Letters have up to 2500 words, including a 180 word introductory paragraph (abstract), and the text is not subdivided in sections.

ACKNOWLEDGEMENTS – should be kept brief. Professional titles and affiliations are unnecessary. Grant numbers can be listed.

AUTHOR CONTRIBUTIONS – must be included after the Acknowledgements, detailing the contributions of each author to the paper (e.g. experimental work, project planning, data analysis etc.). Each author should be listed by his/her initials.

FINANCIAL AND NON-FINANCIAL COMPETING INTERESTS – the authors must include one of three declarations: (1) that they have no financial and non-financial competing interests; (2) that they have financial and non-financial competing interests; or (3) that they decline to respond, after the Author Contributions section. This statement will be published with the article, and in cases where financial and non-financial competing interests are declared, these will be itemized in a web supplement to the article. For further details please see https://www.nature.com/licenceforms/nrg/competing-interests.pdf.

REFERENCES – are limited to a total of 70 for Articles, Resources, Technical Reports; and 40 for Letters. This includes references in the main text and Methods combined. References must be numbered sequentially as they appear in the main text, tables and figure legends and Methods and must follow the precise style of Nature Cell Biology references. References only cited in the Methods should be numbered consecutively following the last reference cited in the main text. References only associated with Supplementary Information (e.g. in supplementary legends) do not count toward the total reference limit and do not need to be cited in numerical continuity with references in the main text. Only published papers can be cited, and each publication cited should be included in the numbered reference list, which should include the manuscript titles. Footnotes are not permitted.

METHODS – Nature Cell Biology publishes methods online. The methods section should be provided as a separate Word document, which will be copyedited and appended to the manuscript PDF, and incorporated within the HTML format of the paper.

Methods should be written concisely, but should contain all elements necessary to allow interpretation and replication of the results. As a guideline, Methods sections typically do not exceed 3,000 words. The Methods should be divided into subsections listing reagents and techniques. When citing previous methods, accurate references should be provided and any alterations should be noted. Information must be provided about: antibody dilutions, company names, catalogue numbers and clone numbers for monoclonal antibodies; sequences of RNAi and cDNA probes/primers or company names and catalogue numbers if reagents are commercial; cell line names, sources and information on cell line identity and authentication. Animal studies and experiments involving human subjects must be reported in detail, identifying the committees approving the protocols. For studies involving human subjects/samples, a statement must be included confirming that informed consent was obtained. Statistical analyses and information on the reproducibility of experimental results should be provided in a section titled “Statistics and Reproducibility”.

All Nature Cell Biology manuscripts submitted on or after March 21 2016 must include a Data availability statement as a separate section after Methods but before references, under the heading "Data Availability”. . For Springer Nature policies on data availability see http://www.nature.com/authors/policies/availability.html; for more information on this particular policy see http://www.nature.com/authors/policies/data/data-availability-statements-data-citations.pdf. The Data availability statement should include:

• Accession codes for primary datasets (generated during the study under consideration and designated as "primary accessions") and secondary datasets (published datasets reanalysed during the study under consideration, designated as "referenced accessions"). For primary accessions data should be made public to coincide with publication of the manuscript. A list of data types for which submission to community-endorsed public repositories is mandated (including sequence, structure, microarray, deep sequencing data) can be found here http://www.nature.com/authors/policies/availability.html#data.

• Unique identifiers (accession codes, DOIs or other unique persistent identifier) and hyperlinks for datasets deposited in an approved repository, but for which data deposition is not mandated (see here for details http://www.nature.com/sdata/data-policies/repositories).

• At a minimum, please include a statement confirming that all relevant data are available from the authors, and/or are included with the manuscript (e.g. as source data or supplementary information), listing which data are included (e.g. by figure panels and data types) and mentioning any restrictions on availability.

• If a dataset has a Digital Object Identifier (DOI) as its unique identifier, we strongly encourage including this in the Reference list and citing the dataset in the Methods.

We recommend that you upload the step-by-step protocols used in this manuscript to protocols.io. More details can found at https://www.protocols.io/help/publish-articles.

DISPLAY ITEMS – main display items are limited to 6-8 main figures and/or main tables for Articles, Resources, Technical Reports; and 5 main figures and/or main tables for Letters. For Supplementary Information see below.

FIGURES – Colour figure publication costs $600 for the first, and $300 for each subsequent colour figure. All panels of a multi-panel figure must be logically connected and arranged as they would appear in the final version. Unnecessary figures and figure panels should be avoided (e.g. data presented in small tables could be stated briefly in the text instead).

All imaging data should be accompanied by scale bars, which should be defined in the legend.
Cropped images of gels/blots are acceptable, but need to be accompanied by size markers, and to retain visible background signal within the linear range (i.e. should not be saturated). The boundaries of panels with low background have to be demarked with black lines. Splicing of panels should only be considered if unavoidable, and must be clearly marked on the figure, and noted in the legend with a statement on whether the samples were obtained and processed simultaneously. Quantitative comparisons between samples on different gels/blots are discouraged; if this is unavoidable, it should only be performed for samples derived from the same experiment with gels/blots were processed in parallel, which needs to be stated in the legend.

Figures should be provided at approximately the size that they are to be printed at (single column is 86 mm, double column is 170 mm) and should not exceed an A4 page (8.5 x 11"). Reduction to the scale that will be used on the page is not necessary, but multi-panel figures should be sized so that the whole figure can be reduced by the same amount at the smallest size at which essential details in each panel are visible. In the interest of our colour-blind readers we ask that you avoid using red and green for contrast in figures. Replacing red with magenta and green with turquoise are two possible colour-safe alternatives. Lines with widths of less than 1 point should be avoided. Sans serif typefaces, such as Helvetica (preferred) or Arial should be used. All text that forms part of a figure should be rewritable and removable.

We accept files from the following graphics packages in either PC or Macintosh format:

- For line art, graphs, charts and schematics we prefer Adobe Illustrator (.AI), Encapsulated PostScript (.EPS) or Portable Document Format (.PDF). Files should be saved or exported as such directly from the application in which they were made, to allow us to restyle them according to our journal house style.

- We accept PowerPoint (.PPT) files if they are fully editable. However, please refrain from adding PowerPoint graphical effects to objects, as this results in them outputting poor quality raster art. Text used for PowerPoint figures should be Helvetica (preferred) or Arial.

- We do not recommend using Adobe Photoshop for designing figures, but we can accept Photoshop generated (.PSD or .TIFF) files only if each element included in the figure (text, labels, pictures, graphs, arrows and scale bars) are on separate layers. All text should be editable in 'type layers' and line-art such as graphs and other simple schematics should be preserved and embedded within 'vector smart objects' - not flattened raster/bitmap graphics.

- Some programs can generate Postscript by 'printing to file' (found in the Print dialogue). If using an application not listed above, save the file in PostScript format or email our Art Editor, Allen Beattie for advice (a.beattie@nature.com).

Regardless of format, all figures must be vector graphic compatible files, not supplied in a flattened raster/bitmap graphics format, but should be fully editable, allowing us to highlight/copy/paste all text and move individual parts of the figures (i.e. arrows, lines, x and y axes, graphs, tick marks, scale bars etc.). The only parts of the figure that should be in pixel raster/bitmap format are photographic images or 3D rendered graphics/complex technical illustrations.

All placed images (i.e. a photo incorporated into a figure) should be on a separate layer and independent from any superimposed scale bars or text. Individual photographic images must be a minimum of 300+ DPI (at actual size) or kept constant from the original picture acquisition and not decreased in resolution post image acquisition. All colour artwork should be RGB format.

EXTENDED DATA FIGURES - When re-submitting your manuscript, please ensure that any supplementary figures and tables that are crucial to the manuscript's conclusions are converted into Extended Data figures and tables to increase visibility of these data. Extended Data figures and tables are online-only (present in the online PDF and full-text HTML versions of the paper), peer-reviewed display items that provide essential background to the article but are not included in the main article due to space constraints. A maximum of ten Extended Data display items (figures and tables) is permitted.

FIGURE LEGENDS – must not exceed 350 words for each figure to allow fit on a single printed NCB page together with the figure. They must include a brief title for the whole figure, and short descriptions of each panel with definitions of the symbols used, but without detailing methodology.

TABLES – main tables should be provided as individual Word files, together with a brief title and legend. For supplementary tables see below.

SUPPLEMENTARY INFORMATION – Supplementary information is material directly relevant to the conclusion of a paper, but which cannot be included in the printed version in order to keep the manuscript concise and accessible to the general reader. Supplementary information is an integral part of a Nature Cell Biology publication and should be prepared and presented with as much care as the main display item, but it must not include non-essential data or text, which may be removed at the editor's discretion. All supplementary material is fully peer-reviewed and published online as part of the HTML version of the manuscript. Supplementary Figures and Supplementary Notes are appended at the end of the main PDF of the published manuscript.

Supplementary items should relate to a main text figure, wherever possible, and should be mentioned sequentially in the main manuscript, designated as Supplementary Figure, Table, Video, or Note, and numbered continuously (e.g. Supplementary Figure 1, Supplementary Figure 2, Supplementary Table 1, Supplementary Table 2 etc.).

Unprocessed scans of all key data generated through electrophoretic separation techniques need to be presented in a supplementary figure that should be labelled and numbered as the final supplementary figure, and should be mentioned in every relevant figure legend. This figure does not count towards the total number of figures and is the only figure that can be displayed over multiple pages, but should be provided as a single file, in PDF or TIFF format. Data in this figure can be displayed in a relatively informal style, but size markers and the figures panels corresponding to the presented data must be indicated.

The total number of Supplementary Figures (not including the "unprocessed scans" Supplementary Figure) should not exceed the number of main display items (figures and/or tables (see our Guide to Authors and March 2012 editorial http://www.nature.com/ncb/authors/submit/index.html#suppinfo; http://www.nature.com/ncb/journal/v14/n3/index.html#ed). No restrictions apply to Supplementary Tables or Videos, but we advise authors to be selective in including supplemental data.

Each Supplementary Figure should be provided as a single page and as an individual file in one of our accepted figure formats and should be presented according to our figure guidelines (see above). Supplementary Tables should be provided as individual Excel files. Supplementary Videos should be provided as .avi or .mov files up to 50 MB in size. Supplementary Figures, Tables and Videos much be accompanied by a separate Word document including titles and legends.

GUIDELINES FOR EXPERIMENTAL AND STATISTICAL REPORTING

REPORTING REQUIREMENTS – We are trying to improve the quality of methods and statistics reporting in our papers. To that end, we are now asking authors to complete a reporting summary that collects information on experimental design and reagents. The Reporting Summary can be found here https://www.nature.com/documents/nr-reporting-summary.pdf)If you would like to reference the guidance text as you complete the template, please access these flattened versions at http://www.nature.com/authors/policies/availability.html.

STATISTICS – Wherever statistics have been derived the legend needs to provide the n number (i.e. the sample size used to derive statistics) as a precise value (not a range), and define what this value represents. Error bars need to be defined in the legends (e.g. SD, SEM) together with a measure of centre (e.g. mean, median). Box plots need to be defined in terms of minima, maxima, centre, and percentiles. Ranges are more appropriate than standard errors for small data sets. Wherever statistical significance has been derived, precise p values need to be provided and the statistical test used needs to be stated in the legend. Statistics such as error bars must not be derived from n<3. For sample sizes of n<5 please plot the individual data points rather than providing bar graphs. Deriving statistics from technical replicate samples, rather than biological replicates is strongly discouraged. Wherever statistical significance has been derived, precise p values need to be provided and the statistical test stated in the legend.

Information on how many times each experiment was repeated independently with similar results needs to be provided in the legends and/or Methods for all experiments, and in particular wherever representative experiments are shown.

We strongly recommend the presentation of source data for graphical and statistical analyses as a separate Supplementary Table, and request that source data for all independent repeats are provided when representative experiments of multiple independent repeats, or averages of two independent experiments are presented. This supplementary table should be in Excel format, with data for different figures provided as different sheets within a single Excel file. It should be labelled and numbered as one of the supplementary tables, titled "Statistics Source Data", and mentioned in all relevant figure legends.

--------- Please don't hesitate to contact NCB@nature.com should you have queries about any of the above requirements -----

----

Version 1:

Decision Letter:

Our ref: NCB-A60029A

4th June 2026

Dear Dr. Dumbović,

Thank you for submitting your revised manuscript "The periphery of nuclear speckles defines a spatially and temporally regulated compartment of long-lived intron-retained RNAs that resolves during mitosis" (NCB-A60029A). It has now been seen by the original referees and their comments are below. The reviewers find that the paper has improved in revision, and therefore we'll be happy in principle to publish it in Nature Cell Biology, pending minor revisions to satisfy the referees' final requests and to comply with our editorial and formatting guidelines.

If the current version of your manuscript is in a PDF format, please email us a copy of the file in an editable format (Microsoft Word or LaTex)-- we can not proceed with PDFs at this stage.

Please note that our articles must have 5 to 8 main figures and they can have up to 10 Extended data (ED) figures. If there are more figures than that, they become supplementary figures. Supplementary materials are less accessed than our main and ED figures so we try to limit the use of supplementary figures as much as we can. Please ensure that all figures fit into a single page (not multiple pages) and adhere to a maximum page size of roughly 180mm wide x 200mm high and use a font size of no smaller than 6pt Arial or Helvetica throughout the figures, to ensure legibility of the figures once resized for publication. Please ensure that the figure panel labels and the panels are not too close to the edge of the page. Currently, the panels of the main figures are too close to the edge of the page.

We are now performing detailed checks on your paper and will send you a checklist detailing our editorial and formatting requirements in about 7-10 days' time. **Please do not upload the final materials and make any revisions until you receive this additional information from us.**

Thank you again for your interest in Nature Cell Biology Please do not hesitate to contact me if you have any questions.

Best wishes,
Sabrya.

Sabrya Carim, PhD
(she/her/hers)
Senior Editor, Nature Cell Biology
Nature Portfolio

Springer Nature
The Campus, 4 Crinan Street, London N1 9XW, UK
sabrya.carim@springernature.com
https://orcid.org/0000-0001-9485-1938

Reviewer #1 (Remarks to the Author):

The authors have responded to the reviewers’ comments sincerely and have appropriately cited previous related studies. Through comparison with these prior reports, the novelty of the present work has become clearer. All the issues raised have been adequately addressed, and therefore the manuscript can be considered to be of a standard suitable for acceptance in Nature Cell Biology.

Minor point: Information regarding statistical analyses is scattered across the main text, figure legends, Methods, and Supplementary Table 5, making it difficult to follow. To avoid confusion for readers, the p-values after correction for multiple testing should be clearly indicated as “adjusted p-values” in the main text and figure legends as well.

Reviewer #2 (Remarks to the Author):

We are fine with the authors' responses and revisions.

Thoru Pederson and Paul Kaufman

Reviewer #3 (Remarks to the Author):

I commend the authors for addressing my previous concerns. In particular, the added comparison to prior detained-intron datasets, the clarification and null-model analysis for peri-speckle localization, the added SON depletion validation, and the new double-thymidine release experiment significantly strengthen the manuscript. The cell-cycle-resolved smRNA FISH data provide important independent support for the conclusion that long-lived intron-retained RNAs remain nuclear through interphase and undergo coordinated processing during mitosis. I have no further major experimental concerns and feel the manuscript is ready for publication.

Version 2:

Decision Letter:

Dear Dr Dumbović,

I am pleased to inform you that your manuscript, "The periphery of nuclear speckles defines a spatially and temporally regulated compartment of long-lived intron-retained RNAs that resolves during mitosis", has now been accepted for publication in Nature Cell Biology. Congratulations!

Thank you for sending us the final manuscript files to be processed for print and online production, and for returning the manuscript checklists and other forms. Your manuscript will now be passed to our production team who will be in contact with you if there are any questions with the production quality of supplied figures and text.

Over the next few weeks, your paper will be copyedited to ensure that it conforms to Nature Cell Biology style. Once your paper is typeset, you will receive an email with a link to choose the appropriate publishing options for your paper and our Author Services team will be in touch regarding any additional information that may be required.

After the grant of rights is completed, you will receive a link to your electronic proof via email with a request to make any corrections within 48 hours. If, when you receive your proof, you cannot meet this deadline, please inform us at rjsproduction@springernature.com immediately.

You will not receive your proofs until the publishing agreement has been received through our system.

Due to the importance of these deadlines, we ask that you please let us know now whether you will be difficult to contact over the next month. If this is the case, we ask you provide us with the contact information (email, phone and fax) of someone who will be able to check the proofs on your behalf, and who will be available to address any last-minute problems.

If you have any questions about our publishing options, costs, Open Access requirements, or our legal forms, please contact ASJournals@springernature.com

Once your paper has been scheduled for online publication, the Nature press office will be in touch to confirm the details. An online order form for reprints of your paper is available at https://www.nature.com/reprints/author-reprints.html. All co-authors, authors' institutions and authors' funding agencies can order reprints using the form appropriate to their geographical region.

Publication is conditional on the manuscript not being published elsewhere and on there being no announcement of this work to any media outlet until the online publication date in Nature Cell Biology.

You may wish to make your media relations office aware of your accepted publication, in case they consider it appropriate to organize some internal or external publicity. Once your paper has been scheduled you will receive an email confirming the publication details. This is normally 3-4 working days in advance of publication. If you need additional notice of the date and time of publication, please let the production team know when you receive the proof of your article to ensure there is sufficient time to coordinate. Further information on our embargo policies can be found here: https://www.nature.com/authors/policies/embargo.html

Please note that *Nature Cell Biology* is a Transformative Journal (TJ). Authors may publish their research with us through the traditional subscription access route or make their paper immediately open access through payment of an article-processing charge (APC). Authors will not be required to make a final decision about access to their article until it has been accepted. Find out more about Transformative Journals

Authors may need to take specific actions to achieve compliance with funder and institutional open access mandates. If your research is supported by a funder that requires immediate open access (e.g. according to Plan S principles or the NIH public access policy) then you should select the gold OA route, and we will direct you to the compliant route where possible. Because authors warrant

under our subscription licensing terms that they haven't committed to licensing any version of their article under a licence inconsistent with the terms of our agreement – including the applicable embargo period – publication under the subscription model isn't suitable for authors whose funders require no embargo.

To assist our authors in disseminating their research to the broader community, our SharedIt initiative provides you with a unique shareable link that will allow anyone (with or without a subscription) to read the published article. Recipients of the link with a subscription will also be able to download and print the PDF.

If your paper includes color figures, please be aware that in order to help cover some of the additional cost of four-color reproduction, Nature Portfolio charges our authors a fee for the printing of their color figures. Please contact our offices for exact pricing and details.

As soon as your article is published, you will receive an automated email with your shareable link.

If you have not already done so, we strongly recommend that you upload the step-by-step protocols used in this manuscript to protocols.io (https://protocols.io), an open online resource that allows researchers to share their detailed experimental know-how. All uploaded protocols are made freely available and are assigned DOIs for ease of citation. Protocols and Nature Portfolio journal papers in which they are used can be linked to one another, and this link is clearly and prominently visible in the online versions of both. Authors who performed the specific experiments can act as primary authors for the Protocol as they will be best placed to share the methodology details, but the Corresponding Author of the present research paper should be included as one of the authors. By uploading your Protocols onto protocols.io, you are enabling researchers to more readily reproduce or adapt the methodology you use, as well as increasing the visibility of your protocols and papers. You can also establish a dedicated workspace to collect your lab Protocols. Further information can be found at https://www.protocols.io/help/publish-articles.

You can use a single sign-on for all your accounts, view the status of all your manuscript submissions and reviews, access usage statistics for your published articles and download a record of your refereeing activity for the Nature Portfolio.

Nature Cell Biology encourages authors presenting evidence for cell, biological, molecular, and genetic interactions to consider communicating these findings using Biofactoid (https://biofactoid.org/). This tool helps users share a searchable representation of interactions (e.g. binding, gene expression, post-translational modification) between genes, gene products, or chemicals. Information added to Biofactoid, with author attribution, is shared on social media and public databases, such as Pathway Commons, where it can be discovered and analyzed in the context of a large and growing corpus of knowledge.

Please feel free to contact us if you have any questions.

With kind regards,

Sabrya.

Sabrya Carim, PhD
(she/her/hers)
Senior Editor, Nature Cell Biology
Nature Portfolio

Springer Nature
The Campus, 4 Crinan Street, London N1 9XW, UK
sabrya.carim@springernature.com
https://orcid.org/0000-0001-9485-1938

Click here if you would like to recommend Nature Cell Biology to your librarian
http://www.nature.com/subscriptions/recommend.html#forms

** Visit the Springer Nature Editorial and Publishing website at www.springernature.com/editorial-and-publishing-jobs for more information about our career opportunities. If you have any questions please click here.**

Open Access This Peer Review File is licensed under a Creative Commons Attribution 4.0 International License, which permits use, sharing, adaptation, distribution and reproduction in any medium or format, as long as you give appropriate credit to the original author(s) and the source, provide a link to the Creative Commons license, and indicate if changes were made.
In cases where reviewers are anonymous, credit should be given to 'Anonymous Referee' and the source.
The images or other third party material in this Peer Review File are included in the article's Creative Commons license, unless indicated otherwise in a credit line to the material. If material is not included in the article's Creative Commons license and your intended use is not permitted by statutory regulation or exceeds the permitted use, you will need to obtain permission directly from the copyright holder.
To view a copy of this license, visit https://creativecommons.org/licenses/by/4.0/

We thank the Reviewers for their thoughtful and constructive evaluation of our manuscript, "*The periphery of nuclear speckles defines a spatially and temporally regulated compartment of long-lived intron-retained RNAs that resolves during mitosis*." Their comments helped us substantially strengthen both the conceptual framing and the experimental support of the study.

In the revised manuscript, we addressed all points raised by the Reviewers through new experiments, additional analyses, and revisions of the text and figures. We added an independent double-thymidine block-and-release experiment that tracks LL-IR-RNA localization and processing across the cell cycle at single-cell resolution, showing that these RNAs remain predominantly nuclear during interphase, undergo a major splicing transition during mitosis, and appear in early G1 as spliced cytoplasmic transcripts. We also added a random-spot null model supporting the specific enrichment of IR-RNAs at the nuclear speckle periphery and performed direct quantitative comparisons with previously annotated detained introns to better position our findings within the prior literature. We further refined the deep-learning analysis distinguishing long- and short-lived retained introns by restricting the classification to introns with basal retention ≥10%, thereby reducing potential leakage from weakly retained introns that may have blurred the earlier comparison; this yielded a more robust and biologically consistent model while improving interpretability of the classification task. Finally, we added control analyses for the kinase-perturbation experiments, verified SON depletion, revised statistical reporting, and made figure and textual revisions.

Please find below our point-by-point responses to the Reviewers' comments.

REVIEWER COMMENTS

Reviewer #1:

In this manuscript, Bianya et al. propose that intron-detained RNAs derived from cell cycle–related genes are pooled at the periphery of nuclear speckles and undergo synchronous splicing at mitosis, thereby contributing to the elaborate regulation of cell cycle–related genes. The authors' observations on the relationship between intron-detained RNAs and nuclear speckles during the mitotic cell cycle are informative and potentially valuable for the readers of Nature Cell Biology.

However, I have two major concerns. First, the authors conclude that "our findings demonstrate that IR is not stochastic, but a regulated program…" (lines 101–104). This concept, however, is neither novel nor original to the authors. Multiple research groups in the RNA field have already proposed and substantiated this idea—commonly referred to as intron detention—over the past decade. The authors do not sufficiently acknowledge previously reported findings, nor do they cite several key papers in this area. Moreover, they introduce a new term for a splicing event already known as intron detention. Altogether, this gives the impression that prior work by other researchers has been underestimated and that the novelty and originality of the present study are overstated. Second, the authors attempt to verify that inhibition of nuclear speckle remodeling during mitosis alters intron detention. To this end, they use a CLK1 inhibitor. However, CLK1 is a well-known kinase that regulates splicing—including intron retention and intron detention—through phosphorylation of SR splicing factors. It is therefore unclear how the authors discriminate between the effects of CLK1 inhibition on nuclear speckle remodeling and its well-established roles in splicing regulation independent of nuclear speckles.

Overall, although I agree that this study potentially highlights the biological importance of intron detention in cell-cycle progression, its conceptual novelty is limited, and the methodology of the core experiments appears flawed in its current form. Additional details and further concerns are described below.

We thank the Reviewer for recognizing the value of the study, while also raising important conceptual concerns that helped us sharpen the framing of our findings. We agree that the phenomenon of intron detention is well established, and we did not intend to imply that it originates with our study. We have revised the manuscript throughout to better acknowledge foundational work in this area, cite the relevant literature more comprehensively, and position our findings more clearly within the existing intron-detention framework.

One of the main advances of our study is the identification, within that broader landscape, of a long-lived subset distinguished operationally by prolonged persistence after transcriptional shutoff and by reproducible spatial and cell-cycle-linked behavior in hiPSCs. Specifically, compared with previously characterized detained introns, including the representative introns reported by Boutz *et al*. (2015),

with a mean half-life of approximately 29 minutes, these introns have substantially longer half-lives, are associated with distinct sequence and structural features, and are enriched at the nuclear speckle periphery. We further show that these RNAs are resolved in a coordinated manner during mitotic remodeling and give rise to spliced cytoplasmic transcripts in early G1. In this way, our study adds a spatial and physiological dimension to existing models of intron detention.

A further advance is the quantitative spatial analysis. Previous work provided important precedents by inferring nuclear speckle association of retained introns from proximity-labeling experiments. What has been missing is a comparative, quantitative analysis across a broad endogenous panel that determines where retained/detained intron-containing RNAs sit relative to nuclear speckle subcompartments. Here, we combine 3D single-molecule RNA imaging with super-resolution microscopy to move beyond general nuclear speckle association and show a reproducible spatial pattern: IR-RNAs are excluded from the SC35-positive core, positioned at the interface between U2 shell and surrounding nucleoplasm, and frequently distributed around multiple nuclear speckles, with the long-lived class localizing most proximally. To our knowledge, this level of spatial resolution has not previously been established for a broad set of detained/retained intron-containing transcripts.

Finally, beyond prior handcrafted feature-based classifiers, our study uses a novel RNA AI foundation model to learn the properties of introns directly from sequence and to distinguish retained from efficiently spliced introns, as well as long-lived from short-lived retained introns. This points to combinatorial sequence, structural, and RBP-associated features linked to prolonged intron retention.

Together, these findings extend current models of intron detention by integrating kinetic, spatial, and cell-cycle–dependent regulation. We have revised the statement indicated by the Reviewer accordingly (**lines 132-136**): "*Together, our data support a model in which a subset of long-lived retained introns shows reproducible peri-nuclear speckle positioning and preferential resolution during mitosis, linking cis-encoded intronic features, nuclear organization, and cell-cycle timing, potentially supporting mitotic inheritance of mRNAs essential for genome maintenance and cell cycle regulation.*"

Major Points

R1.1. Several relevant papers are not cited.

DOI: 10.1083/jcb.201107093 (the first report describing signal-induced post-transcriptional splicing of intron-detained RNAs in the nucleus)

DOI: 10.1080/15384101.2017.1337983 (germ cell differentiation–dependent regulation of intron detention)

DOI: 10.1101/gr.273904.120 (a comprehensive analysis of intron retention and detention in subnuclear compartments)

DOI: 10.15252/embj.2019102729 (a nuclear MLO that controls intron detention)

These papers should be cited and discussed in the context of the current study.

We appreciate the Reviewer highlighting these studies. We agree that they are important to frame the manuscript more completely, and we have now incorporated them into the revised Introduction, Results, and Discussion as indicated below:

10.1083/jcb.201107093 and 10.15252/embj.2019102729, **lines 73-74**: "*Furthermore, detained introns can be resolved in response to specific signaling pathways and cues*[8,19,20,23,35]"; **lines 863-868**: "*More broadly, CLK-dependent control of IR has been described in other nuclear contexts in interphase*[19,35,81]*. For instance, CLK activity promotes or suppresses splicing of specific detained introns; CLK-dependent SR protein phosphorylation in nuclear stress bodies and CLK2 condensates has been linked to increased IR, whereas CLKs are themselves autoregulated through nuclear IR-Clk1/4 transcripts*[19,35,81,132]*.*"

10.1101/gr.273904.120, **lines 71-73**: "*Subcellular fractionation-based transcriptome studies have shown that many intron-retaining transcripts are polyadenylated yet remain nuclear/chromatin-anchored, supporting IR as a mechanism to delay mRNA export*[34]*.*"; **lines 214-215**: "*The vast majority of IR events were enriched in nuclear RNA (Fig. 2B), consistent with prior reports*[18–20,34]*.*"

10.1080/15384101.2017.1337983, **lines 869-875**: "*Interestingly, a long-lived IR program has been described in male germ cells, where IR-RNAs encoding key proteins for male gamete function persist*

throughout differentiation, with splicing delayed until post-meiotic stages of spermatogenesis, when de novo mRNA synthesis is largely shut down[133]. It is therefore plausible that large-scale nuclear reorganization events, such as those accompanying mitosis or meiosis, create regulatory windows that enable coordinated intron resolution, thereby replenishing the cytoplasmic mRNA pool when transcription is transiently repressed or delayed."

R1.2. The analyses presented in Figures 1 and 2 are informative but conceptually not novel, as several groups have previously reported similar bioinformatic analyses examining features of detained introns such as functional enrichment, intron length, and GC content.

DOI: 10.1101/gad.247361.114

DOI: 10.1101/gad.247361.114

DOI: 10.1101/gr.273904.120

Some points highlighted by the authors (e.g., enrichment in cell-cycle and DNA damage–related genes) have already been described in these studies. The authors should discuss prior findings more fairly and explicitly compare their results with previously reported data. It is also important for the authors to clarify which aspects of their findings truly provide novel or advanced insights. Moreover, it is unclear why the authors introduce a new term, "LL-IR RNAs," for a class of RNAs widely known as intron-detained RNAs. Intron-detained RNAs are defined as partially spliced RNAs containing one or more introns that are stably retained in the nucleus and that undergo splicing upon specific stimulation and/or function as a regulatory buffer. This definition is conceptually and essentially identical to LL-IR RNAs.

We thank the Reviewer for this important comment. We agree that the global feature analyses in Fig. 1B,C are not, on their own, the conceptual advance of the study and our intention was not to present them as such. Fig. 1B,C are presented as a dataset-level characterization showing that IR events in hiPSCs display global properties reported previously in other cell types. This provides the context for the later analyses, which resolve these events into kinetically and spatially distinct subclasses. We have revised the manuscript to state this more explicitly.

The main advance emerges from Fig. 1D onward; by combining transcriptional inhibition kinetics, imaging-based spatial analysis, and cell-cycle-resolved measurements, we define a long-lived nuclear intron-retained RNA subpopulation, which is localized to a conserved nuclear speckle-peripheral compartment and is subjected to coordinated resolution during mitosis. In this sense, the novelty lies not in the enrichment of IR within cell-cycle- or DNA damage-related genes per se, but in the finding that IR events in these genes display higher magnitudes of temporal stability than other IR events thereby allowing their long-lived nuclear persistence. We have revised the text throughout to make this distinction clearer and to avoid overstating aspects that were established.

As suggested by the Reviewer, we performed a quantitative comparison between our classes and detained introns previously annotated by Boutz *et al*. (2015) and Braun *et al*. (2018). These analyses are now included in **Suppl. Fig. 3**, **Suppl. Fig. 4M,N,** and summarized below in **Reviewer Fig. 1** for convenience. Importantly, previously annotated detained introns do not map selectively to our long-lived class. Instead, they are spread across multiple categories in our dataset. From the two datasets, approximately 42-56% of annotated detained introns fall below our baseline nuclear PIR threshold and map to the no IR category, likely reflecting differences in cell type and event definition. Among those that are assigned to our kinetic classes, approximately 59-61% mapped to the collectively unstable groups, and 39-41% mapped to the stable group. Only a minority of our stable introns overlap previously annotated detained introns. This indicates that annotated detained introns and the long-lived IR class defined here are partially overlapping, but not synonymous, populations.

The distinction is further supported by quantitative differences in half-life, basal PIR, GC content, intron length, nuclear speckle enrichment, and machine-learning-based classification. Boutz *et al*. reported an average half-life of approximately 29 min for representative detained introns. Consistent with this, in our dataset, introns corresponding to annotated detained introns remain substantially shorter-lived (mean half-life 3.2-3.4 h) than the long-lived class defined here (mean half-life 9.99 h). Moreover, the long-lived class shows higher basal PIR compared to annotated detained introns (mean ~66% vs. ~35–42%, respectively), higher GC content (mean ~54% vs. ~46–47%, respectively), shorter length (mean $\log_{10}$(length) ~2.86 vs. ~3.07–3.21, respectively), and stronger nuclear speckle enrichment (mean $\log_2$FC 1.6 vs. 0.8, respectively) (**Suppl. Fig. 3, Suppl. Fig. 4N, Reviewer Fig. 1**). Functionally,

detained introns were enriched primarily in RNA processing pathways, stress response, DNA damage, and cell-cycle regulation, consistent with the overall enrichment of IR-containing genes reported in Fig. 1B. By contrast, the long-lived class identified here is more specifically enriched in genes linked to mitosis, DNA repair, and genome maintenance.

Finally, to assess whether previously defined detained introns more closely resemble long-lived or short-lived IR classes defined in our manuscript, we applied our machine learning model to annotated detained intron sequences and observed that they predominantly cluster with unstable (short-lived) retained introns (**Suppl. Fig. 4M, Reviewer Fig. 1E**), indicating shared sequence and structural features. This helps further differentiate the population of long-lived retained introns described here from the previously reported detained introns.

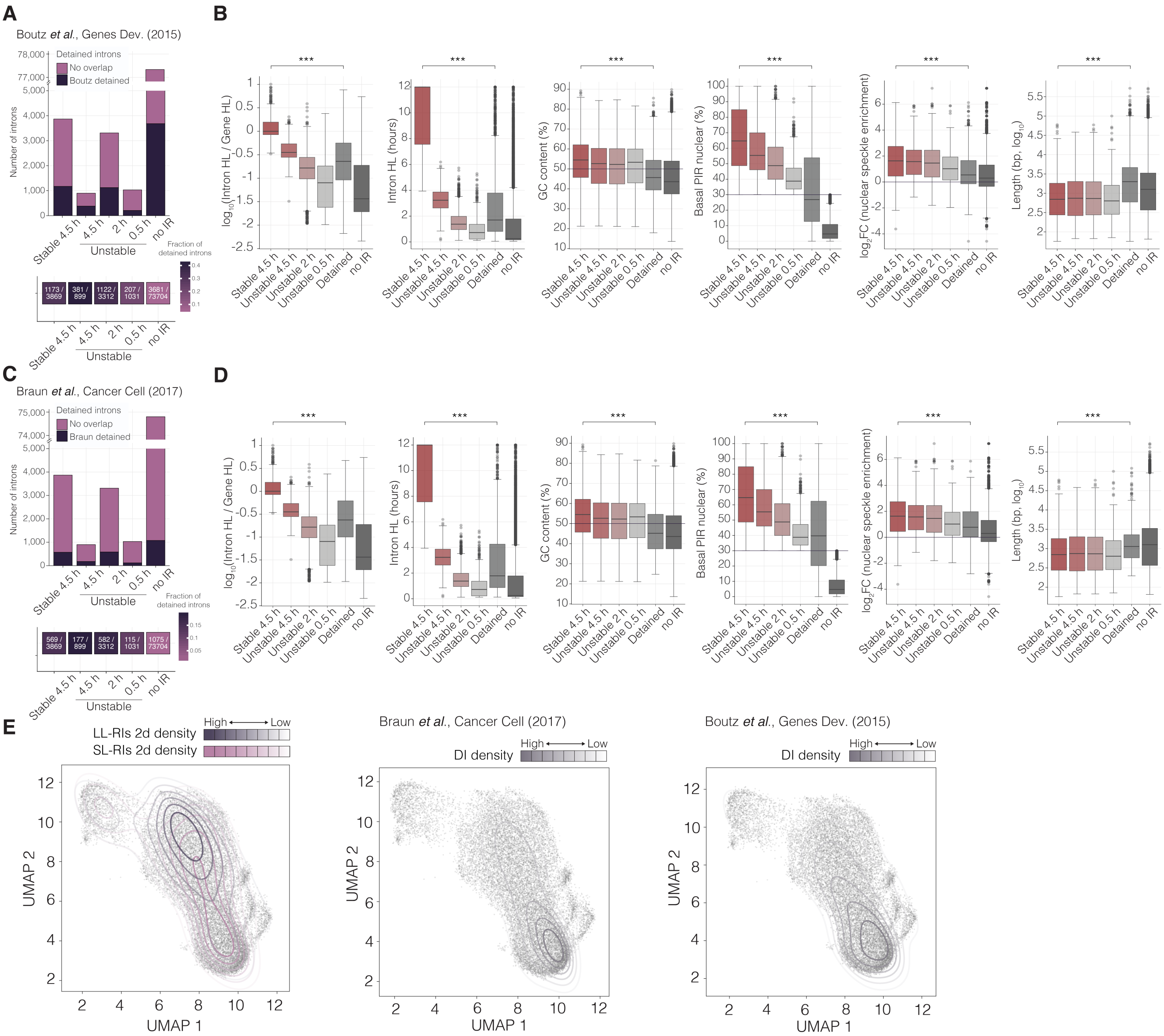

Reviewer Fig. 1 (from Suppl. Fig. 3 and Suppl. Fig. 4M,N). Comparison of detained introns with intron retention stability, sequence features, and nuclear speckle enrichment. (**A**, **C**) Number and fraction of introns overlapping previously defined detained introns (Boutz *et al.,* (2015) and Braun *et al.*, (2017)) across IR stability categories and non-retained introns (no IR). Stacked bar plots show the number of introns with (dark) or without (light) overlap. The y-axis is truncated to allow visualization of the no IR group. Below, the fraction of detained introns (overlapping/total) for each category. (**B**, **D**) Comparison of molecular features across categories. From left to right: intron retention half-life (HL) normalized with gene half-life ($\log_2$ intron HL / gene HL), intron half-life (HL; hours), GC content (%), basal nuclear percent intron retention (PIR; %), and nuclear speckle enrichment ($\log_2$ fold change derived from Wu et al., 2024). ***$P \leq 0.001$, Tukey's HSD test, Benjamini–Hochberg (FDR) correction. **E.** Left, UMAP projection of Parnet intron embeddings fine-tuned on IR HL. Each point, an intron; distances reflect similarity in the model's learned features. Contour lines, kernel density estimates for the intron classes labelled above, indicating where introns from each class are concentrated in the embedding space. Middle and right, contour lines, kernel density estimates for the detained introns.

These differences are consistent with the distinct ways in which the two groups were defined. Our stable class was defined in nuclear poly(A)$^+$ RNA by persistence after transcriptional inhibition, using a baseline PIR threshold together with a requirement that introns retain at least 50% of their initial IR level after 4.5 h of transcription inhibition, thereby specifically enriching for introns with prolonged nuclear persistence. In contrast, previously described detained introns were not defined by half-life, although they were shown to persist longer than efficiently spliced introns (Boutz *et al.*, 2015).

We also appreciate the Reviewer's point regarding terminology. Our intention in using LL-IR and SL-IR was to introduce an operational classification based strictly on persistence after transcriptional inhibition. We agree, however, that the relationship to detained introns should be made clearer. In the revised manuscript, we clearly state that the long-lived class identified here is best understood as a distinct population within a broader intron detention landscape characterized by longer persistence, with mitosis acting as a major splicing trigger. We made adjustments in the Introduction (**lines 62–79, 125-128**), Discussion (**lines 787-790, 863-868**), as well as added the quantitative comparison with previously annotated detained introns in the Results (**lines 300–324**, **386-389**, **701-705**, **760-764**, **Suppl. Fig. 3**, **Suppl. Fig. 4M,N**). These additions clarify both the points of overlap and of distinction.

We have extensively discussed among authors and with colleagues whether to change the terminology to intron-detained RNAs. We feel that retaining LL-IR / SL-IR as operational labels for subclasses defined uniformly by persistence after transcriptional shutoff is useful, without presuming that every event belongs to the classical detained-intron category. That said, we are fully open to follow the Editors' and Reviewers' preferred nomenclature to improve clarity.

R1.3. The authors use CLK1 and DYRK3 inhibitors to manipulate nuclear speckle remodeling during mitosis. However, these kinases are well-established regulators of splicing—including intron detention—through phosphorylation of SR proteins and SR-related factors. In particular, multiple reports have shown that CLK1 activity modulates splicing of intron-detained RNAs independently of the cell cycle. Because SRSFs also localize broadly within the nucleoplasm and some are enriched in nuclear bodies other than speckles, it remains unclear how the authors distinguish whether the observed effects result from inhibition of nuclear speckle remodeling rather than from altered SRSF phosphorylation outside of speckles. The authors should clarify this point and cite more appropriate, up-to-date studies on CLK1 and its role in splicing regulation, including intron detention.

The Reviewer raises an important point. We agree that CLK family kinases regulate nuclear speckle organization as well as phosphorylation of SR proteins, including in the nucleoplasm and other nuclear bodies. We therefore do not interpret the inhibitor experiments as perturbing nuclear speckle remodeling in isolation. This distinction is particularly relevant in mitosis, when nuclear speckles and other nuclear bodies are not organized as in interphase, but dissolve and their content becomes intermixed with nucleoplasmic splicing factors as part of a broader remodeling program. We have revised the manuscript to clarify this point and describe these perturbations as interfering more broadly with mitosis-associated remodeling, a process accompanied by changes in SR-protein phosphorylation state, condensate disassembly, and redistribution of splicing factors.

Importantly, within our dataset, the strongest effects of CLKi/DYRK3i are observed in mitosis across the LL-IR candidates examined, whereas interphase responses are limited. By contrast, canonical CLK-sensitive detained introns, such as those in SRSF7 from Boutz *et al*., show the expected interphase response to CLK inhibition (this analysis has been added to **Suppl. Fig. 10B** and **lines 760-764**). These observations suggest that the LL-IR class studied here is distinguished by a predominant dependence on mitosis-associated remodeling programs.

Finally, we have added the relevant literature on CLK-dependent regulation of intron detention, including work on nuclear stress bodies, to better frame these experiments in the context of prior studies in the Results (**lines 760-764**) and Discussion (**lines 863-868**).

R1.4. In the description of Figure 3, the authors should discuss previous findings that GC-rich introns and shorter introns are enriched in nuclear speckles. DOI: 10.1016/j.molcel.2021.12.010 (reference 33)

We have added a sentence in the Results section (**lines 373-375**) stating that our findings highlighted in Suppl. Fig. 4F showing, among others, a correlation between GC content, length and nuclear speckle

enrichment, are consistent with previous observations based on proximity-labeling analyses reported in reference 33 showing that GC-rich and short retained introns associate with nuclear speckle markers. Please note that the analyses in Fig. 3 and Suppl. Fig. 4-5 focus on the sequence and genomic properties of long- versus short-lived retained introns, in addition to retained versus non-retained introns. In this context, the novelty of our analysis lies in linking these sequence and structural features to intron persistence, and in integrating them with our spatial and kinetic data. We have revised the text accordingly to better acknowledge the prior study while clarifying what is new in our analysis.

R1.5. In Figures 6A and 6B, the authors present only the ratio of exon to intron detention. To exclude the possibility that intron-detained RNAs are simply degraded, they should also show quantitative changes in the levels of spliced mRNAs.

We thank the Reviewer for this important point. The original version of Figures 6A and 6B, in addition to PIR (%), included the absolute smFISH spot counts per cell corresponding to (i) intron-retained transcripts, (ii) spliced transcripts (Δ retained intron), and (iii) free intron for each gene. In the revised manuscript, this analysis has been moved to **Suppl. Fig. 9B**. These data show that the spliced mRNA signal increases in mitosis, accompanied by a proportional increase in the abundance of free intron, consistent with active splicing of intron-retained RNAs rather than degradation. The mean values are summarized in the corresponding Research section, **lines 640-655**. Additionally, in the revised manuscript, we synchronized the cells using a double thymidine block, and followed transcript states across interphase, mitosis, and early G1 (new **Fig. 6A,B;** please also see reply **R3.8** for more details). We performed the same quantitative analysis (total RNA count, spliced RNA count, IR-RNA count, cytoplasmic RNA count, free intron count), which confirms that spliced mRNA increases specifically in mitosis, whereas the intron-retained RNA decreases. Importantly, we observe a reciprocal increase in free intron count in mitosis, indicating splicing. Interestingly, the spliced RNA generated in mitosis is equally distributed to the cytoplasm of daughter cells without the loss of total RNA quantity. These data support that LL-IR-RNAs are spliced during mitosis and subsequently inherited, rather than being eliminated by decay. In the revised manuscript, we added the quantitative changes in spliced mRNAs and IR-RNAs, alongside PIR (%), to the kinase inhibition experiment, now in **Fig. 7B**.

R1.6. In the Materials and Methods section, statistical procedures are specified only for Figure 3 and Supplementary Figures 3D and 4A. According to other figure legends, the authors repeatedly used t-tests for multiple comparisons. If the reported p-values were adjusted for multiple testing (e.g., using FDR or Bonferroni correction), the correction procedures should be specified in each legend or in the Methods section. If no corrections were applied, the authors should re-evaluate significance using methods appropriate for multiple comparisons. Additionally, the type of statistical test used in Supplementary Figure 7 is not indicated.

We have now revised the statistical reporting across the manuscript to ensure that the statistical test used for each analysis, the sample size, and the handling of multiple comparisons are described consistently in the figure legends and/or in the Methods section under **Statistical reporting**. For analyses involving multiple comparisons, *P* values were adjusted using Benjamini–Hochberg procedure, which is now specified in the Methods section (**lines 1290-1303**) and documented in **Suppl. Table S5**. We have also added the missing statistical test information for Suppl. Fig. 7 (now **Suppl. Fig. 10**).

R1.7. The methods for the FISH experiments in Figures 5 and 6 are insufficiently described, making it difficult to understand how "nuclear RNA abundance" was quantified. It is also confusing that the terms "nuclear RNA abundance" and "nuclear RNA enrichment" are used interchangeably despite appearing to refer to the same measurement. The terminology should be unified and the quantification method clearly explained.

We have now unified the terminology throughout the manuscript and consistently use the term "nuclear transcript fraction". In the Methods section, we have added a clear definition of how this and other metrics were quantified from smRNA FISH experiments, **lines 1240-1248**: "*Transcription sites were identified based on overlap between efficiently spliced intron signals and exon signals (for Fig. 4J, Suppl. Fig. 8B) or by high-intensity nascent RNA foci characteristic of active transcription (for Suppl.*

Fig. 8B). Nuclear transcript fraction was quantified as the fraction of exon smRNA FISH spots detected in the nucleus relative to the total number of exon spots per cell, calculated as $N_{exon,nuc}$ / ($N_{exon,nuc}$ + $N_{exon,cyt}$), where $N_{exon,nuc}$ and $N_{exon,cyt}$ denote the numbers of exon spots detected in the nucleus and cytoplasm, respectively. Cytoplasmic transcript fraction was defined analogously as $N_{exon,cyt}$ / ($N_{exon,nuc}$ + $N_{exon,cyt}$). Nuclear PIR was calculated as $N_{IR,nuc}$ / $N_{exon,nuc}$, and total PIR as $N_{IR,nuc}$ / ($N_{exon,nuc}$ + $N_{exon,cyt}$), where $N_{IR,nuc}$ denotes the number of nuclear IR-RNA spots." We have also ensured consistent usage of this term across the Results, figure legends, and axis labels.

R1.8. The list of speckle-associated RBPs used for the analysis in Figure 3 is not provided. It should be included in the supplementary materials.

In the revised manuscript, we included the list of nuclear speckle-associated RBPs used for the analysis in Figure 3 as **Suppl. Table S3**. As described in the Methods section (**lines 1125-1128**) and under Data availability (**1317-1319**), this set was downloaded from the *RBP Image Database* resource from the Lecuyer lab (https://rnabiology.ircm.qc.ca/RBPImage/). RBPs with a quality score > 1 that were detected in nuclear speckles in either HepG2 or K562 cell lines were selected. Please note that the same annotation appears to have been used in the previous study (reference 33), based on their methods description and cited sources, although we could not find in this paper the full list of nuclear speckle RBP annotations to cross-check.

Minor Points

R1.9. In the smFISH protocol, the description "smRNA FISH exon/intron probes (1:100)" (line 977) is unclear because the stock concentration of the probes is not specified.

We thank the Reviewer for pointing this out. The smRNA FISH protocol in the Methods section has been expanded to include a detailed description of probe labeling and to specify both the probe stock concentration and the working dilution used in the assay.

R1.10. Some explanations are insufficient in the text and figures. The significance of the long arrow in Figure 1E is not described. The meaning of "UT" in Figure 2A is unclear. "HL" (line 298) should be defined.

We have revised Fig. 1F and associated legend to clarify that the long arrow indicates increasing cytoplasmic transcript enrichment across the three localization phenotypes.

In Fig. 2A, "UT" referred to the untreated condition. To ensure consistency with the terminology used throughout the manuscript, we have replaced "UT" with "NT" and clarified this condition in the figure legend.

In addition, we have defined "HL" as "half-life" at its first occurrence in the text (**line 312**) and clarified this term in the corresponding figure legend.

R1.11. Several terms should be standardized for consistency:

SC35-rich domain / SC35 domain / SC35-dense interior class / type "Exon RNA FISH spots" (Figure 1D) vs. "Exon nuclear" (Figure 5), if they refer to the same measurement.

We have standardized the terminology throughout the manuscript, main figures, and supplementary figures. All SC35-related terminology has been unified and is now referred to as the "SC35-positive nuclear speckle core" throughout the text and figure legends.

In addition, exon smRNA FISH quantifications have been harmonized across all figures. We refer to compartment-specific exon measurements as "nuclear exon spots" and "cytoplasmic exon spots," and specify the RNA species when applicable (e.g., "intron-retained RNA spots," "spliced exon RNA spots"). Axis labels and legends in both main and supplementary figures have been updated accordingly to ensure consistent terminology.

R1.13. Typographical errors:

"mature RNA mRNA" (line 58), "untread" (line 183), Duplicate sentence in Supplementary Figure 1 legend:

"Introns selected for smRNA FISH are marked with a purple asterisk."

We thank the Reviewer for pointing out these typographical errors. We have corrected the typographical mistakes and removed the duplicate sentence in the Suppl. Fig. 1 legend.

Reviewer #2

This is an impressive study with several elements of novelty. Although unspliced mRNA was found localized in nuclear speckles decades ago, the present findings extend this specifically to transcripts whose splicing is kinetically slow relative to the majority. The work is technically well executed, and the conclusions are strongly supported by the data. This paper reminds all of us in the nuclear organization and function field that we still have new things to learn.

We thank the Reviewers for their positive assessment of our manuscript and for their suggestions.

The authors should consider the following points.

R2.1. It would be good to emphasize that this peri-speckle localization is not at the nuclear bodies known as "paraspeckles". The authors could cite the fact that paraspeckles do not contain the SON protein and thus would not be captured by the designed proximity ligation method. They could also cite literature that pluripotent stems cells typically lack paraspeckles.

The Reviewer raises an excellent point, and we agree that it is important not to confuse the peri-nuclear speckle localization of IR-RNAs with paraspeckles. Following the Reviewer's recommendation, we have added the following sentences in the Discussion, **lines 800-805**: "*Importantly, the IR-RNA localization described here should not be confused with paraspeckles, a distinct nuclear body that is typically absent in pluripotent stem cells and becomes detectable upon differentiation[123,124]. Consistent with this, paraspeckles do not contain the nuclear speckle scaffold protein SON[125], and therefore SON-based proximity labeling approaches would not be expected to capture paraspeckle-associated RNAs.*"

R2.2. In the abstract, the phrase "and are released to the cytosol" could be rephrased to just "are released", since the nuclear envelope disassembles in mitosis (as do the speckles themselves) and thus everything that was intranuclear is "released".

We have revised the wording in the Abstract as suggested.

R2.3. Unless NCB allows speculation in the Abstract, the authors should eliminate the last sentence of the second paragraph. They are of course free to so speculate in the Discussion. The Abstract also seems much too long and could be shortened if the handling editor concurs.

We removed the final sentence of the second paragraph and shortened the Abstract.

R2.4. Where in the nucleus splicing occurs was a very controversial issue, with some groups claiming it is co-transcriptional* and others saying it requires that the pre-mRNA be released from the transcription site and arrive at a speckle. The authors need not write a minireview on this but should mention this more in their Introduction and Discussion. They might also cite an important early paper in the field that demonstrated that if the transcriptional intensity of a gene is very high then splicing factors are recruited there in high concentrations and thus a speckle forms de novo (Huang and Spector, Genes Dev. 5: 2288-2302, 1991.

We thank the Reviewer for this suggestion. We have added a brief discussion in the Introduction and Discussion acknowledging the longstanding debate regarding co-transcriptional versus post-transcriptional splicing. We cite early work demonstrating that highly active transcription sites can recruit splicing factors and form enlarged speckle-like SC35-positive domains at the locus (Huang and Spector, 1996), as well as earlier observations linking nascent transcripts to nuclear speckles (Huang and Spector, 1991).

Introduction, **lines 92-97**: "*Where and when splicing occurs within the nucleus has been examined extensively, with evidence supporting both co- and post-transcriptional splicing[51–60]. Early studies*

showed that nascent transcripts from strongly induced genes localize near nuclear speckles, supporting dynamic coupling between transcriptional activity and nuclear speckle domains[61]. Consistent with this, highly active transcription sites can recruit splicing factors and generate enlarged nuclear speckle-like domains at the locus[62]."

Discussion, **lines 798-800**: "*These findings further inform the longstanding models of splicing, supporting the view that splicing can occur both co- and post-transcriptionally, long after transcript release from the transcription site*."

R2.5. Line 528: "We hypothesize this mechanism ensures their inheritance by newly formed daughter cells and translation in early G1 without requiring new transcription, splicing and export, which can take tens of minutes to over an hour in mammalian cells." It would be good for the authors to see whether they can see long-lived IR spots in post-mitotic cells, e.g. neurons.

We thank the Reviewer for this suggestion and agree that testing whether long-lived IR-RNAs persist in post-mitotic cells (e.g., neurons, cardiomyocytes) is an important future direction. We applied our smRNA FISH pipeline to iPSC-derived cardiomyocytes; however, high intrinsic background/autofluorescence prevented reliable single-molecule detection under our current conditions. Establishing and optimizing post-mitotic differentiation models for quantitative IR-RNA imaging would require substantial additional time since each differentiation process takes between 3-4 weeks and is beyond the scope of the current revision. Importantly, we do not propose that LL-IR-RNAs are spliced exclusively during mitosis; rather, our data support mitosis as a major route for coordinated resolution of a large fraction of the long-lived nuclear IR-RNA pool, generating translation-competent transcripts for early G1. This clarification has been incorporated into the Discussion by adding a corresponding statement in **lines 855-857**. We agree with the Reviewer that it will be important to determine the behavior of long-lived IR-RNAs in post-mitotic cells, and we consider this to be an important direction for further investigation.

R2.6: Line 152-3 "Retained intron signals were frequently, but not necessarily, detected at or near transcription sites." One has to find the legend for Sup. Fig. 7 to see how transcription sites were detected in these experiments. This should be described in more detail in the methods.

We have added a detailed description in the Results and Methods section of how transcription sites were identified in smRNA FISH experiments, **lines 556-558**: "*Transcription sites were identified based on overlap between exon and efficiently spliced intron signals, with one to two punctate nuclear intron signals co-localizing with exon signal marking active loci (Suppl. Fig. 8A).*" and **lines 1240-1242**: "*Transcription sites were identified based on overlap between efficiently spliced intron signals and exon signals (for Fig. 4J, Suppl. Fig. 8B) or by high-intensity nascent RNA foci characteristic of active transcription (for Suppl. Fig. 8B).*"

* Although not a point the authors need to mention but something to always be borne in mind is that all three of the first pre-mRNAs to be identified (β-globin, ovalbumin and the adenovirus tripartite leader) involved Northerns of oligo(dT)-cellulose selected RNA and thus their subsequent splicing was post-transcriptional.

Thoru Pederson and Paul D. Kaufman, UMass Chan Medical School

Reviewer #3

This is a peer review of "Nuclear speckle periphery organizes intron-retained RNAs into a distinct nuclear retention compartment that resolves during mitosis" from Biayna et al. The authors begin by showing that intron retention is increased in the nucleus compared to the cytoplasm (fig 1). Using global transcriptional inhibition they show that a subset of retained introns in the nucleus are particularly stable (fig 2). They use a machine learning approach to link IR-RNAs with nuclear speckles through RBP motif enrichment (fig 3) which they then confirm by analzying TSA-seq and using smFISH (fig 4). However, they go on to show that nuclear speckles are not required to observe the intron-retention in that class of RNAs (fig 5). Interestingly, the authors demonstrate that mitosis is associated with the

eventual splicing of the those RNAs (fig 6). Finally, the authors attempt to associate specific mitotic kinase activities with the intron retention (fig 6).

We thank the Reviewer for the positive assessment of our manuscript and their suggestions.

Comments

R3.1. The manuscript contains two key findings. First, a subset of detained intron containing transcripts localize to the periphery of nuclear speckles. Second, mitosis is a trigger for splicing many of those transcripts.

R3.2. Stable intron-containing transcripts that remain in the nucleus have been known for quite a while (Boutz et al 2015). Given that these early studies were also done in stem cells, the transcripts identified in this study should be compared with those previously identified.

In the revised manuscript, we now performed a detailed quantitative comparison between our stability classes and previously reported detained introns from Boutz *et al*. (2015), doi: 10.1101/gad.247361.114 and Braun *et al*. (2018) doi: 10.1016/j.ccell.2017.08.018 (results of this comparison are summarized in **Suppl. Fig. 3, Suppl. Fig. 4M,N**; see also response **R1.2** and **Reviewer Fig. 1** below). By mapping gene and intron coordinates across datasets, we assigned previously annotated detained introns to our stability classes. In both cases, detained introns were distributed across multiple stability groups, with a substantial fraction mapping to unstable classes and a minority overlapping with the long-lived class. Thus, we show that the long-lived class identified here is not restricted to previously defined detained introns, but represents a distinct population within a broader intron detention landscape.

This distinction is further supported by different intron properties. Boutz *et al.* reported an average half-life of approximately 29 minutes for detained introns; consistent with this, mapped detained introns in our dataset show substantially shorter half-lives (3.2–3.4 h) compared to long-lived IRs (~9.99 h). In addition, the long-lived class displays higher basal PIR levels, higher GC content, shorter length, and stronger nuclear speckle enrichment (please see **lines 300-324**, and reply **R1.2**).

Finally, to assess whether previously defined detained introns more closely resemble long-lived or short-lived IR classes at the sequence level, we used our deep learning model trained to distinguish these classes. When embedding the sequences of detained introns, they predominantly clustered with unstable introns, occupying regions of the embedding space corresponding to this class (**Suppl. Fig. 4M**), with only a small fraction overlapping with long-lived IRs. Together, these analyses provide a comprehensive comparison with prior studies and refine the distinction between defined classes.

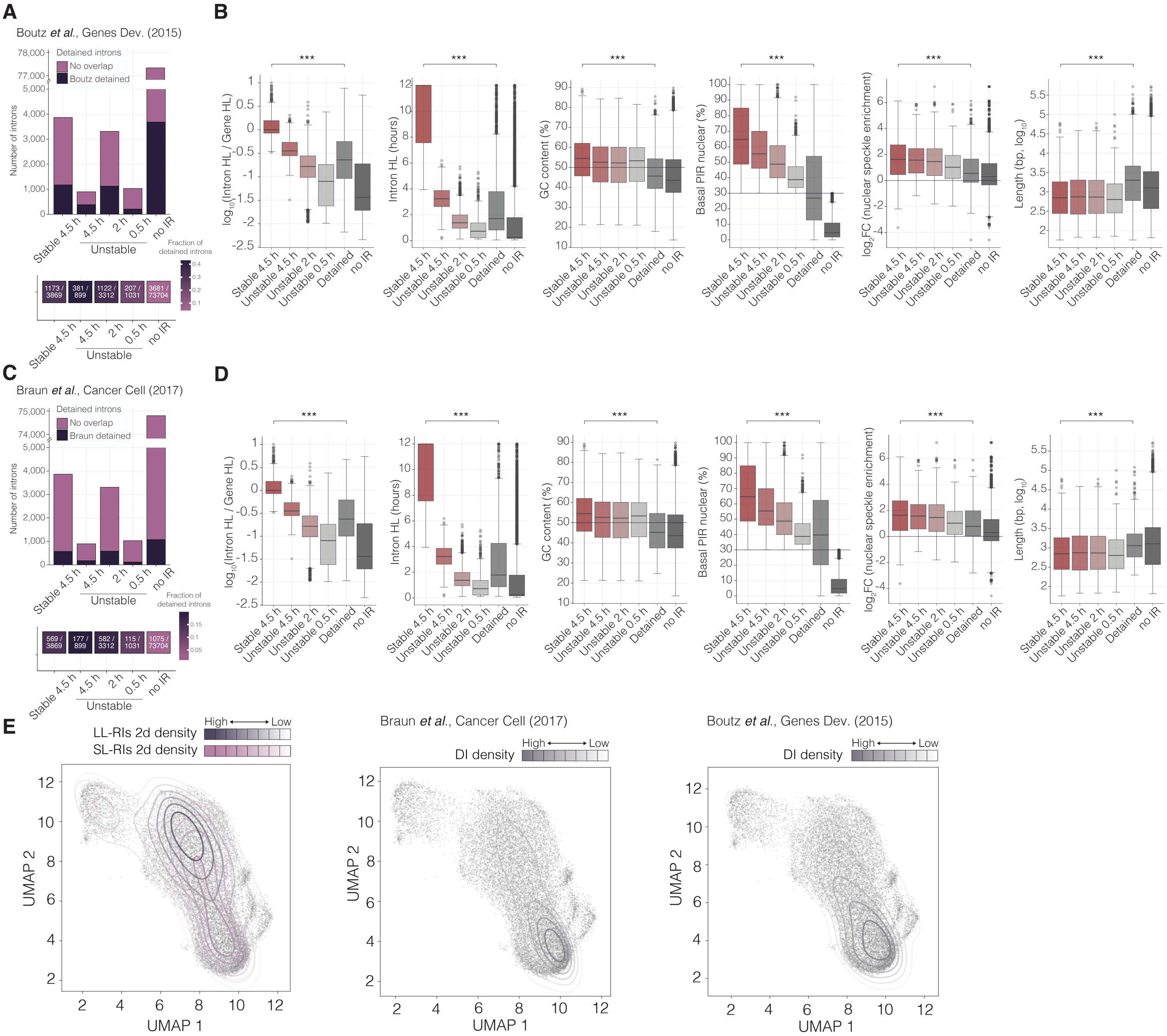

Reviewer Fig. 1 (from Suppl. Fig. 3 and Suppl. Fig. 4M,N). Comparison of detained introns with intron retention stability, sequence features, and nuclear speckle enrichment. (**A**, **C**) Number and fraction of introns overlapping previously defined detained introns (Boutz *et al.,* (2015) and Braun *et al.*, (2017)) across IR stability categories and non-retained introns (no IR). Stacked bar plots show the number of introns with (dark) or without (light) overlap. The y-axis is truncated to allow visualization of the no IR group. Below, the fraction of detained introns (overlapping/total) for each category. (**B**, **D**) Comparison of molecular features across categories. From left to right: intron retention half-life (HL) normalized with gene half-life (log_2 intron HL / gene HL), intron half-life (HL; hours), GC content (%), basal nuclear percent intron retention (PIR; %), and nuclear speckle enrichment (log_2 fold change derived from Wu et al., 2024). ****P* ≤ 0.001, Tukey's HSD test, Benjamini–Hochberg (FDR) correction. **E**. Left, UMAP projection of Parnet intron embeddings fine-tuned on IR HL. Each point, an intron; distances reflect similarity in the model's learned features. Contour lines, kernel density estimates for the intron classes labelled above, indicating where introns from each class are concentrated in the embedding space. Middle and right, contour lines, kernel density estimates for the detained introns.

R3.3. It seems the conclusion of figure 1 is that RNAs in the nucleus tend to have more introns. Wouldn't this be the expected result based on first principles, since nuclear RNAs were likely more recently produced and may still be undergoing processing?

We agree with the Reviewer that enrichment of intron-containing RNAs in the nucleus is expected in principle, because nuclear RNA pools include newly transcribed and incompletely processed transcripts. Nuclear enrichment alone therefore does not imply regulated or functional intron retention.

Our experimental design aimed to minimize detection of nascent transcripts by selecting poly(A)$^+$ RNA to enrich transcripts that underwent cleavage and polyadenylation and have therefore completed co-transcriptional splicing. In addition, we applied a PIR threshold to ensure that the introns analyzed

represent bona fide retained/detained introns within otherwise processed transcripts. Under these conditions, the main observation of Fig. 1 is the presence of spatially distinct distribution patterns of intron-retained RNAs at single-molecule resolution. Specifically, we observe (i) intron-retained signals that remain frequently localized near transcription sites; and (ii) intron-retained RNAs that are broadly distributed throughout the nucleoplasm, indicating transcripts that have been released from transcription sites and have resided in the nucleus long enough to redistribute spatially after release from their transcription sites. Therefore, Fig. 1 provides orthogonal single-molecule evidence that a behavior previously seen in isolated transcript-specific cases extends to an unbiased set of poly(A)$^+$ high-PIR RNAs, patterns that cannot be distinguished from compartment-resolved RNA-seq, since both appear as high-PIR introns. By establishing this, Fig. 1 provides the framework for the subsequent analyses of IR persistence, nuclear speckle proximity, and mitotic resolution presented in later figures.

We have added a new panel to **Fig. 1D** and revised the text to clarify this point, **lines 191-194**: "*Importantly, spatial distribution cannot be inferred from PIR values in compartment-resolved RNA-seq, since both transcription-site–proximal and broadly nucleoplasmic IR-RNAs can appear as high PIR events.*"

R3.4. Actinomycin D is a pretty stressful treatment for cells and is likely triggering stress response pathways. This is a minor issue, but metabolic labeling of RNAs, perhaps with a pulse of 4sU would likely have been a more subtle approach.

We thank the Reviewer for this helpful suggestion. We agree that Actinomycin D is a perturbation and that metabolic labeling approaches such as 4sU pulse-chase provide an important complementary way to study RNA dynamics. At the same time, metabolic labeling approaches also have technical limitations, and, depending on labeling conditions, can affect cellular physiology, including RNA processing, cell proliferation, and stress-responsive pathways (PMID: 28219744, 39548324, 24025460, 34898625, 38381903). In the present study, transcriptional shutoff was used to assess the persistence of the pre-existing nuclear intron-retained RNA pool after ongoing transcription had ceased. Importantly, our conclusions are supported by multiple orthogonal approaches, including single-molecule imaging and cell-cycle synchronization experiments.

R3.5. The path from a machine learning model based on eCLIP data to the proposal that these transcripts are speckle associated is not clearly described.

We agree with the Reviewer that this point was not sufficiently clear, likely due to the multiple orthogonal analyses related to nuclear speckle-associated RBPs. We provide a new panel figure (**Suppl. Fig. 4E**) showing the same foundation model embedding as in Fig. 3E, the representation of SL-RIs versus LL-RIs learned by the model, projected onto a two-dimensional space, but now displaying all data points and coloring them by the number of nuclear speckle RBP ENCODE peaks (same data used for model training).

While this does not directly probe individual embedding dimensions, which is computationally more challenging and is the task of future work on our foundation model, it provides an intuitive way to assess what the embedding captures. Notably, the color gradient reveals that LL-RIs are associated with higher speckle RBP binding, indicating that the model encodes, at least in part, intronic features related to nuclear speckle association and distinguishes stable from unstable retained introns accordingly.

Importantly, Fig. 3G further confirms this result using orthogonal data (where the gradient effect is even stronger), ARTR-seq data, a measure of enrichment (log fold change) of nuclear speckle markers from publicly available data (reference 80): when coloring the same embedding by this independent measurement, LL-RIs remain more strongly enriched for nuclear speckle markers (supported statistically by a significantly higher association of LL-IRs for speckle markers compared to SL introns, **Fig. 3H**). This demonstrates that the effect and association of LL RIs with nuclear speckles are robust, confirmed by orthogonal analysis, and captured by the model, and is not solely driven by ENCODE-based annotations. We have now clarified this in the **Results section 3**.

R3.6. The authors should clarify what negative distance to a speckle means (fig 4C and elsewhere). In addition, the peri-speckle assertion requires some sort of null distribution to compare to. I.E. If one

chose random spots in the nucleus, what would the expected distribution of distances to nearest speckles look like.

We have now clarified in the Fig. 4C-F legend and Methods that distances were computed relative to the segmented nuclear speckle surface (stained with SC35 or U2), such that positive values indicate RNA spots located outside the segmented speckle surface and negative values indicate spots located within the segmented surface (distance measured inward from the mask boundary).

To quantitatively assess the peri-nuclear speckle enrichment, we generated a null distribution by sampling random spots of the same size as smRNA FISH spots within the nuclear mask and computing their distances to the nearest nuclear speckle surface using the same analysis pipeline. Compared with this random distribution, IR-RNA distances were strongly shifted toward the nuclear speckle boundary (one-sided KS test; $P \leq 5.1\times10^{-36}$), supporting that IR-RNAs are enriched at the nuclear speckle periphery rather than being randomly positioned within the nucleus relative to nuclear speckles. These results have now been included in the Results section (**lines 454-492**), and corresponding Methods section has been added (**lines 1260-1270**).

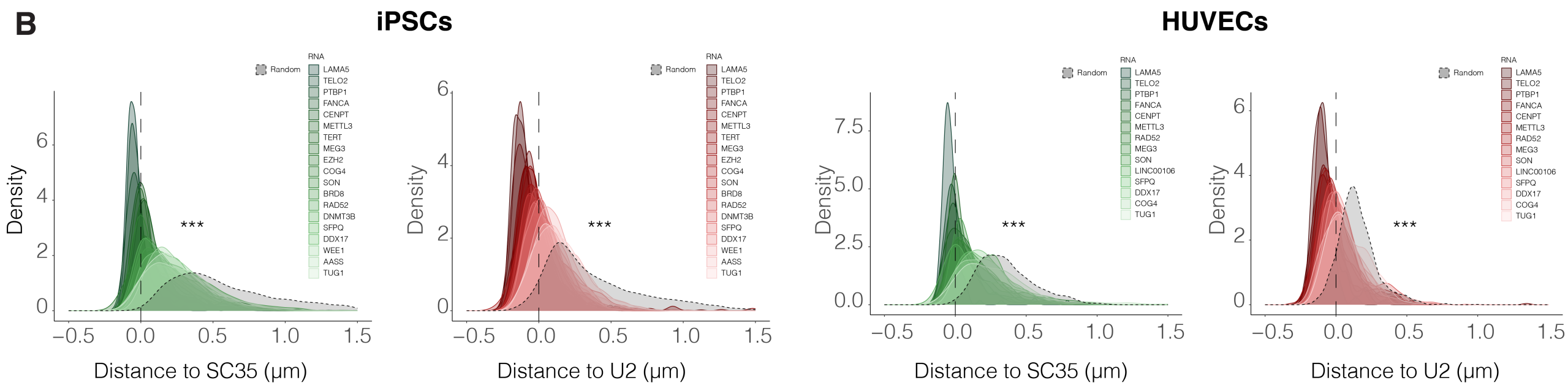

Supplementary Figure 6B. Subcellular localization of IR-RNAs relative to nuclear speckles in hiPSCs and HUVECs. B. Density plots of the distance (µm) of IR-RNA molecules and random spots to nuclear speckle surface. Distances were computed to the segmented U2 and SC35 boundary for iPSCs (left) and HUVEC (right). ***P ≤ 0.001, as evaluated by one-sided KS test random spots versus individual IR-RNAs.

R3.7. It is not clear from the images shown if SON has been depleted by it's shRNA. Removal of both SON and SRRM2 is required to prevent speckle formation.

We thank the Reviewer for raising this point. We have now performed SON immunofluorescence following individual and combined SON and SRRM2 knockdown to verify depletion and assess nuclear speckle integrity. The experiment showed reduced SON immunofluorescence signal upon siSON, confirming successful depletion of SON. These data have been added to the updated **Suppl. Fig. 8C** (panel on the right side), summarized below for convenience. We have also updated the Results and Methods sections accordingly (**lines 585** and **1224**).

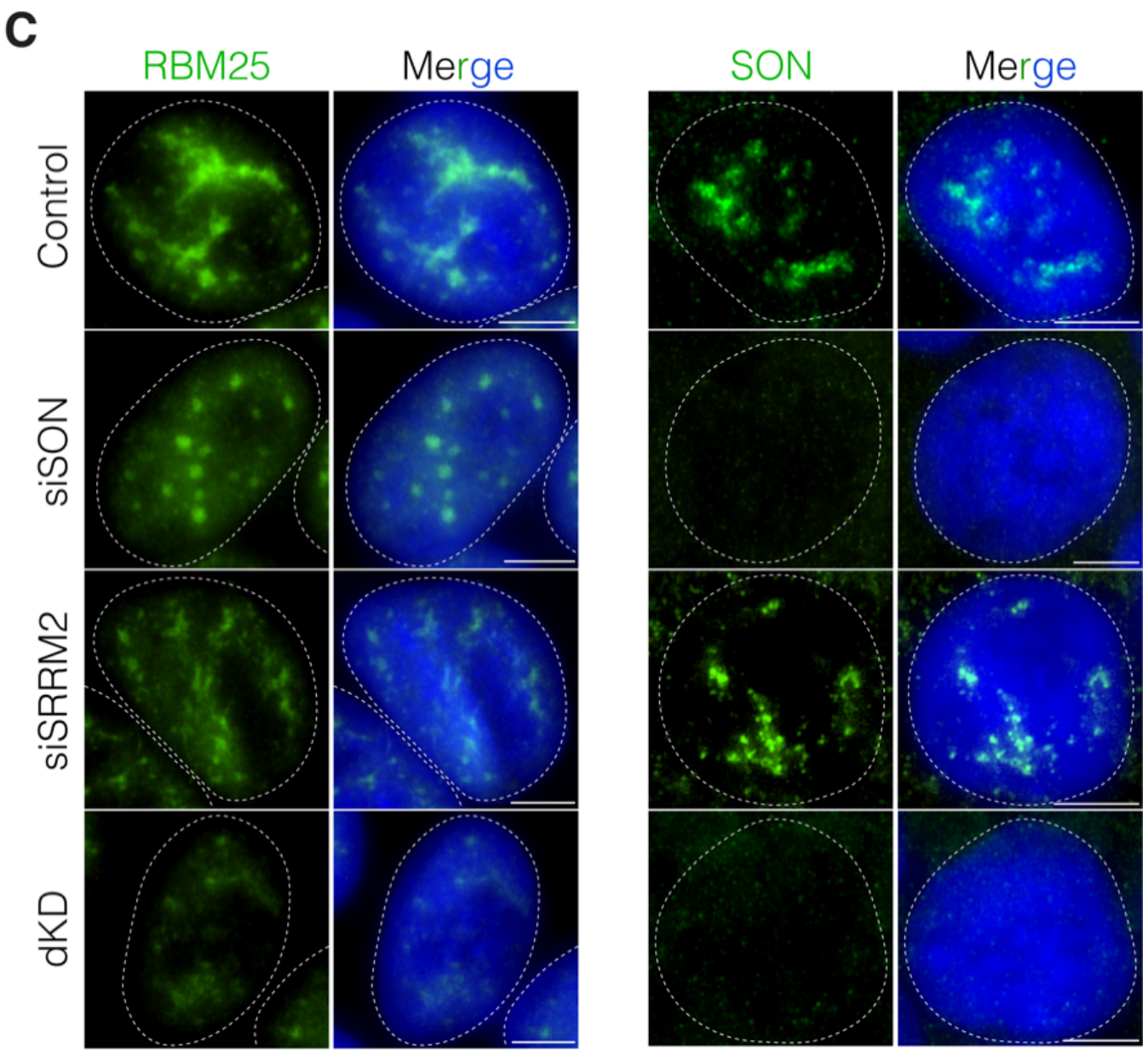

Supplementary Fig. 8C. RBM25 and SON IF (green) after SON, SRRM2 or SON/SRRM2 knockdown. Nucleus, blue. Scale, 2 µm.

R3.8. Mitosis acting as a trigger for the splicing of these detained introns is a very interesting observation. However, besides stating that they are using nocodazole, there is no description in the methods about how this is being done. Is this a nocodazole block for the cells labeled "M" and those labeled G2. Nocodazole is supposed to block the cell cycle at prometaphase, so I'm a bit confused about how the G2 cells are obtained.

It would be supportive to use another method of synchronizing cells like a thymidine block so that cells could be tracked following release from the block to see if splicing in coincident with entry into M phase. Another prediction would provide needed support for the authors assertions would be that time spent in the thymidine block would lead to more accumulation of the unspliced nuclear transcripts, since you are delaying M phase entry. Given the importance of this finding to the novelty of the work, additional support and details for the role of mitotic entry is critical.

We thank the Reviewer for these constructive suggestions. We agree that directly following IR-RNA localization and processing across the cell cycle substantially strengthens the conclusion that mitosis provides a major physiological window for LL-IR-RNA resolution. We have addressed the Reviewer's points as follows:

First, we expanded the Methods section to provide a detailed description of the nocodazole treatment (**lines 929-930**). In that experiment, nocodazole was used to enrich hiPSCs in late prometaphase. Because unsorted nocodazole-treated cultures are not completely homogeneous and retain a small fraction of interphase cells (PMID: 30595546), cell identity was assigned at single-cell resolution. Mitotic cells were identified by chromosome condensation together with phospho-Histone H3 positivity. A minority of cells (~20%) within the same population remained in interphase, which is consistent with prior reports (PMID: 30595546). These cells were phospho-Histone H3-negative, and lacked S-phase PCNA replication foci, corresponding to G2 phase based on these combined features. Since the revised manuscript now includes a cell cycle-resolved experiment after double thymidine block (see below), we have simplified the terminology and now refer to this fraction as interphase.

Second, and most importantly, we complemented the nocodazole experiment with an independent double-thymidine block-and-release strategy, as suggested by the Reviewer. This allowed us to follow IR-RNA dynamics through early and late S phase, G2, mitosis, and early G1 at single-cell resolution using PCNA-based staging. These new data are now included in **Fig. 6** and **Suppl. Fig. 9C,D,** with Fig. 6 pasted below for Reviewers' convenience. The results show that these RNAs remain intron-retained and predominantly nuclear throughout S and G2 phases, and then undergo a sharp and coordinated transition during mitosis characterized by a loss of intron-retained RNA signal, an increase in spliced transcripts, and a reciprocal increase of free intron signal, as we can also observe in nocodazole-synchronized and unsynchronized cells. Free intron signal is rarely detected during interphase, and its emergence in mitosis therefore provides strong evidence for fresh intron release from the retained RNA, consistent with active splicing at this stage. Importantly, total RNA abundance is preserved, arguing against degradation and supporting mitosis-coupled processing. After mitotic exit, daughter cells in early G1 are dominated by cytoplasmic spliced transcripts, the number of which corresponds to approximately half of the spliced transcripts generated in M phase, consistent with inheritance of the mitotically processed RNA pool. The Results (**lines 656-700**) and Methods (**lines 930-940**) have been updated accordingly. Together, these additions provide independent evidence that a major splicing wave of LL-IR-RNAs is tightly coupled to mitosis.

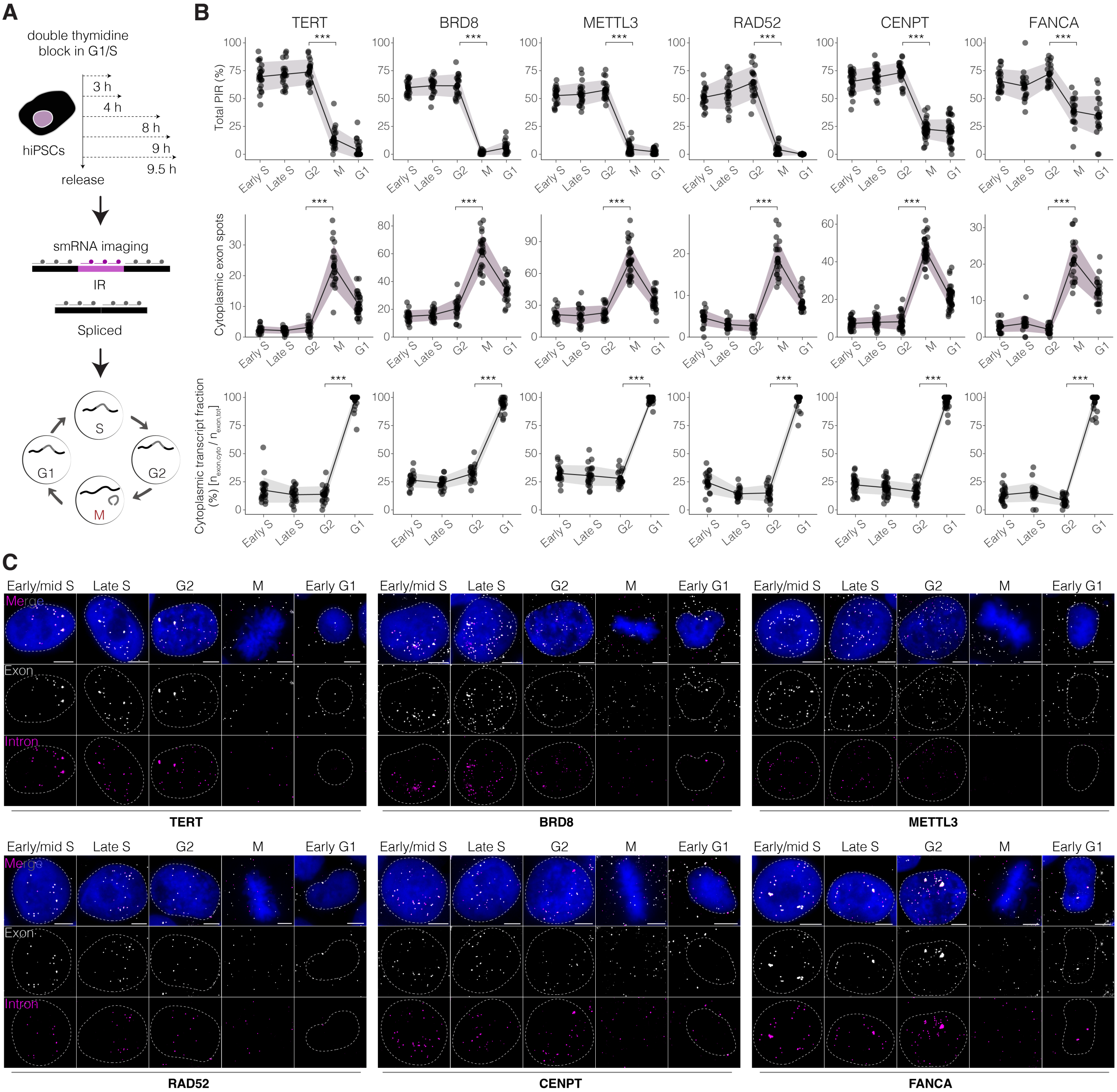

Fig. 6. **Mitosis drives splicing of stable IR-RNAs and cytoplasmic accumulation of their spliced mRNA. A.** Experimental design scheme. hiPSCs were synchronized by double thymidine block at the G1/S boundary, released, and analysed by smRNA FISH at the indicated time points corresponding to early/mid S, late S, G2, M and early G1. **B**. Quantification of total PIR (top), cytoplasmic exon spots (middle), and cytoplasmic transcript fraction [$n_{exon,cyto}/n_{exon,total}$] (bottom) for the indicated RNAs across synchronized cell-cycle stages. Each dot represents one cell. Black line, mean value; shadow, SD; n = 20-45 cells, three independent experiments. ***$P \leq 0.001$, evaluated by two-sided Wilcoxon rank-sum test versus G2. **C**. Representative smRNA FISH maximum intensity projections of TERT, BRD8, METTL3, RAD52, CENPT and FANCA in synchronized hiPSCs at the indicated cell-cycle stages. Exon, white; intron, magenta; nucleus, blue, outlined with a dashed line, scale, 2 µm.

We thank the Reviewers for their positive evaluation of our manuscript, "*The periphery of nuclear speckles defines a spatially and temporally regulated compartment of long-lived intron-retained RNAs that resolves during mitosis*."

Please find below our point-by-point responses to the Reviewers' comments.

REVIEWER COMMENTS

Reviewer #1:

The authors have responded to the reviewers' comments sincerely and have appropriately cited previous related studies. Through comparison with these prior reports, the novelty of the present work has become clearer. All the issues raised have been adequately addressed, and therefore the manuscript can be considered to be of a standard suitable for acceptance in Nature Cell Biology.

Minor point: Information regarding statistical analyses is scattered across the main text, figure legends, Methods, and Supplementary Table 5, making it difficult to follow. To avoid confusion for readers, the p-values after correction for multiple testing should be clearly indicated as "adjusted p-values" in the main text and figure legends as well.

We indicate adjusted *P* values as *P*adj in the main text and in the figure legends.

Reviewer #2:

We are fine with the authors' responses and revisions.

Thoru Pederson and Paul Kaufman

Reviewer #3:

I commend the authors for addressing my previous concerns. In particular, the added comparison to prior detained-intron datasets, the clarification and null-model analysis for peri-speckle localization, the added SON depletion validation, and the new double-thymidine release experiment significantly strengthen the manuscript. The cell-cycle-resolved smRNA FISH data provide important independent support for the conclusion that long-lived intron-retained RNAs remain nuclear through interphase and undergo coordinated processing during mitosis. I have no further major experimental concerns and feel the manuscript is ready for publication.
